# Photoactivatable Fluorogenic Labeling via Turn‐On “Click‐Like” Nitroso‐Diene Bioorthogonal Reaction

**DOI:** 10.1002/advs.201802039

**Published:** 2019-05-02

**Authors:** Bai Li, Xian‐Hao Zhou, Peng‐Yu Yang, Liping Zhu, Yuan Zhong, Zhengjun Cai, Biao Jiang, Xiaoqing Cai, Jia Liu, Xianxing Jiang

**Affiliations:** ^1^ Guangdong Key Laboratory of Chiral Molecule and Drug Discovery School of Pharmaceutical Sciences Sun Yat‐Sen University Guangzhou Guangdong 510006 China; ^2^ Shanghai Institute of Materia Medica Chinese Academy of Sciences Shanghai 201210 China; ^3^ Shanghai Institute for Advanced Immunochemical Studies ShanghaiTech University Shanghai 201210 China; ^4^ University of Chinese Academy of Sciences Beijing 100049 China

**Keywords:** fluorogenic labeling, nitroso‐diene cycloaddition, photoactivatable, protein imaging

## Abstract

Fluorogenic labeling enables imaging cellular molecules of interest with minimal background. This process is accompanied with the notable increase of the quantum yield of fluorophore, thus minimizing the background signals from unactivated profluorophores. Herein, the development of a highly efficient and bioorthogonal nitroso‐based Diels–Alder fluorogenic reaction is presented and its usefulness is validated as effective and controllable in fluorescent probes and live‐cell labeling strategies for dynamic cellular imaging. It is demonstrated that nitroso‐based cycloaddition is an efficient fluorogenic labeling tool through experiments of further UV‐activatable fluorescent labeling on proteins and live cells. The ability of tuning the fluorescence of labeled proteins by UV‐irradiation enables selective activation of proteins of interest in a particular cell compartment at a given time point, while leaving the remaining labeled molecules untouched.

## 1 Introduction

Molecular imaging is one of the most powerful tools in biomedical research that provides researchers with the possibility to visually understand the diverse and complex biological processes.^1^ Considerable efforts have been made to discover and expand the imaging tools, including fluorescent proteins (FPs),^2^ peptide‐based and protein‐based genetic tags^3^ as well as small molecule probes.^4^ Labeling of proteins of interest (POIs) with fluorescent probes via “bioorthogonal” chemistries^5^ allows to uncover their localization, trafficking, and interaction network in live cells.^6^ Despite these advantages, traditional fluorescent labeling often suffers from high background signals due to the excess fluorescent tags or probes and the incomplete removal of free dye molecules from cells, thereby complicating the interpretation of the native functions of POIs. In addition, most commonly used fluorescent dyes are often unstable and sensitive to light and moisture. Therefore, the development of efficient fluorogenic probes with zero background signals is highly desirable.

**Figure 1:**
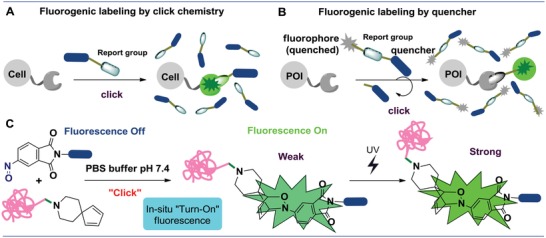
Different fluorogenic approach for protein labeling. A) Fluorogenic labeling based on ligation reactions. B) Fluorogenic labeling based on a fluorescence quenching strategy. C) In situ generated light‐activatable probes via fluorogenic nitroso‐diene pairs.

Fluorogenic labeling can be leveraged for a wide range of applications particularly in combination with bioorthogonal chemistries^7^ (**Figure** **1**A) such as Cu(I)‐catalyzed azide‐alkyne^8^ cycloaddition and strain‐promoted azide‐alkyne cycloadditions.^9^ In addition to azide‐alkyne chemistry, other bioorthogonal reactions have also been developed to facilitate activatable fluorogenic labeling, including Staudinger ligation^10^ and tetrazine‐alkyne,^11^ tetrazine‐alkene,^12^ or photoactivatable tetrazole‐alkene cycloadditions.^13^ Notably, Bertozzi and co‐workers developed azido‐fluorescein^14^ and azido‐rhodamine^7^ fluorogenic probes for wash‐free biological imaging in live cells. Alternatively, Wong and co‐workers described cell‐permeable borondipyrromethene (BODIPY)‐based fluorogenic probes.^15^ With a slightly different setup, Kikuchi and co‐workers^16^ developed fluorogenic probes, which were activated by the removal of a quencher moiety (Figure 1B). Despite the progress, it remains difficult to synthesize fluorogenic probes in high yield and purity, which makes the modification of their structures even more challenging. In addition, the dynamic range of fluorogenic labeling is restrained by the limited photoactivation efficiency of fluorogenic probes.

The hetero‐Diels–Alder cycloaddition (HDA)‐based “bioorthogonal” ligation has emerged as a powerful, chemoselective conjugation tool to facilitate biomolecular labeling.[qv: 11a,17] However, the discovery of new HDA‐based bioorthogonal reactions remains a major challenge because such reactions should meet the strict requirements of click chemistry, including high reactivity and selectivity of functional groups, good stability in aqueous solution, and excellent biocompatibility and high reaction rate under physiological conditions. Excitingly, we found that the nitroso‐Diels–Alder (nitroso‐DA) has several attractive properties for forming fluorescent entity. For example, nitroso‐DA can efficiently deliver the rigid complex polycyclic twisted electron “donor–acceptor–donor” conjugated structural system, a necessary component for fluorogenic probes. In addition, the reaction can proceed rapidly in aqueous phase system under mild conditions, which is appealing for biological and macromolecular settings. These advantages that make them ideal to address challenges associated with current success of nitroso‐based DA as a “bioorthogonal” ligation in this area. Herein, we first report the rational design and experimental validation of nitroso‐functionalized fluorogenic probes that enable effective and controllable biological imaging (Figure 1C). Most importantly, the nitroso‐diene probe could further be activated upon UV irradiation, thus enabling spatial and temporal control of the labeling process.

## 2 Results

### 2.1 Evaluation of Nitroso/Diene Precursors for Fluorogenic Nitroso‐DA

**Figure 2:**
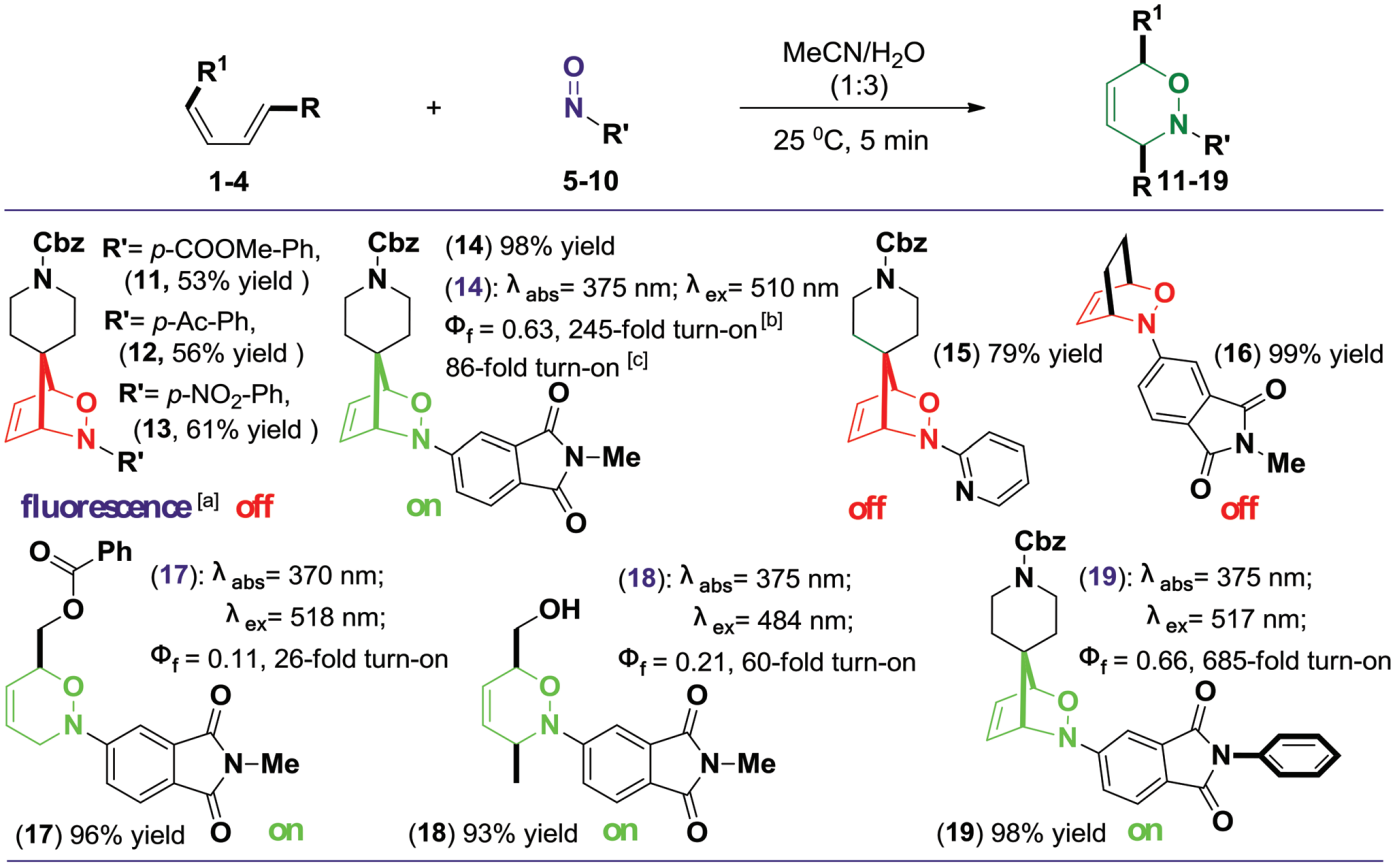
Evaluation of the cycloaddition reactivity of different diene/nitroso compounds. Unless noted otherwise the reaction was conducted with 0.22 mmol of dienes **1–4** and 0.20 mmol nitroso compounds **5–10** for 5 min at 25 °C. Yield of isolated product. ^a)^Fluorescence screening reactions of **11–19** by a UV lamp excitation (λ_ex_ = 365 nm). ^b)^Fluorescein (in 0.1 m NaOH) was used as the standard for quantum yield measurement, and **14** was tested after 2.0 min under visible‐light or UV illumination. ^c)^ **14** was tested directly after reaction.

We present herein the rational design and experimental validation of a novel fluorogenic probe labeling approach via nitroso‐DA cycloaddition. To begin our initial investigation, a variety of substituted nitroso compounds and dienes (**Figure** **2** and Figure S1, Supporting Information) were evaluated for both reaction rate and fluorescence activation under mild conditions (acetonitrile/H_2_O: 1:3 at 25 °C). These reactions generally underwent rapid and clean conversion, affording the desired products with isolated yields ranging from 73% to 99% within 5 min (Figure 2). Optimum reactivity was observed between *N‐*substituted phthalimide nitroso compounds (**8** or **9**) and diene (**1**), affording a fluorescent product **14** or **19** with 98% yield, respectively. Reaction between **1** and other nitroso compounds (**5**–**7** and **10**) gave lower yield and nonfluorescent cycloaddition products (**11**–**13** and **15**). In addition, reaction between **8** and other diene compounds (**2**–**4**) yielded products with no (**16**) or low (**17** and **18**) fluorescence, though the reaction yielding **18** proceeded with the fastest rate (Figure S2, Supporting Information). The second‐order rate constant for the reaction was determined by mixing 1.0 × 10^−3^ m **8** with an excess (10.0 × 10^−3^ m) of diene in 80% *N*,*N*‐dimethylformamide (DMF), 20% phosphate buffer saline (PBS) 1x pH 7.4 buffer at 25 °C. These results suggested that the reactions between *N*‐substituted phthalimide nitroso compounds and diene **1** could provide an efficient fluorogenic labeling approach. Since nitroso compound is typically deemed reactive, we sought to test the stability of **8** and found that it was stable under a variety of pH (Figure S4, Supporting Information) or under long‐term storage (Figure S5, Supporting Information). Notably, **14**, **17**, **18**, and **19** bearing a rigid *N*‐substituted phthalimide acceptor moiety and the imide skeleton as donor moiety exhibited not only excellent photoelectric properties but also strong electron‐withdrawing ability, which is promising for the design of novel and efficient like thermally activated delayed fluorescence (TADF) based photoactivatable probes with a twisted “donor–acceptor–donor” structure by further optimization.^18^

### 2.2 Characterization of Fluorescent Probes

**Figure 3:**
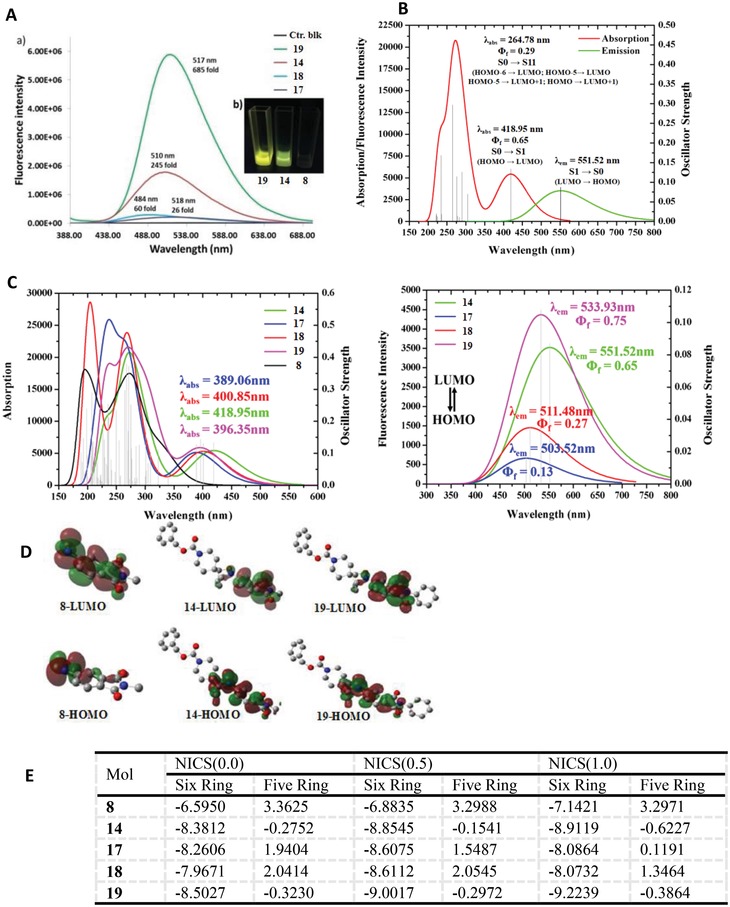
Fluorescence spectra of the cycloaddition products. A) Emission spectra of the cycloaddition products **14**, **17**, **18**, and **19**. Fluorescence emission spectra were tested in 10.0 × 10^−6^ m in 5% DMSO water; excitation at 370 nm. Compounds were tested directly after reaction under UV illumination. Equimolar solutions (10.0 × 10^−6^ m) of compounds under excitation by a handheld UV lamp. B) The absorption and fluorescence spectrum of compound **14**, generated by quantum calculation. **14** have two absorption peaks, one is 264.78 nm which leads to the fluorescence emission with a low quantum yield (Φ_f_ = 0.29), the rest one is 418.95 nm which produces a high quantum yield (Φ_f_ = 0.65). C) The absorption and fluorescence spectrum of compounds **8**, **14**, **17**, and **18**, generated by quantum calculation. The structure optimization as well as the final absorption and fluorescence spectrum were all performed at the B3LYP/6‐311++G (d, p) level with polarizable continuum model (PCM) solvent continuum models (ethanol). D) HOMO–LUMO diagrams of compounds **8** (left) and **14** (right) in the ethanol. E) The calculating NICS (nucleus independent chemical shifts) values of phthalimide. Six Ring, means the six‐membered ring of phthalimide; Five Ring, means the five‐membered ring of phthalimide. NICS(0.0), NICS(0.5), and NICS(1.0) represent the NICS of different positions, respectively.

The formation of fluorescent or nonfluorescent compounds could be easily monitored upon irradiation at 365 nm with a UV lamp, and the formed products **14**, **17**, **18**, and **19** exhibited the expected fluorescence in the preliminary fluorescence screening experiments. Characterization of the fluorescent cycloaddition products (**14**, **17**, **18**, and **19**) revealed a maximum absorption wavelength of around 375 nm (Figure S3, Supporting Information) and a peak emission wavelength ranging from 484 to 518 nm. The large stoke shift of more than 100 nm would allow flexible labeling strategies particularly in combination with other fluorescent dyes.^19^ Excitingly, *N‐*methylphthalimide substituted **14** displayed a strong fluorescence at 510 nm (Φ_f_ = 0.63, λ_abs_ = 375 nm), and the turn‐on with 254‐fold increase compared with its precursor **8** in fluorescence intensity by UV‐irradiation, rivaling most of the existing fluorogenic labeling approaches (**Figure** **3**A). The highest turn‐on of 685‐fold was observed in *N‐*phenylphthalimide substituted **19** at 517 nm. By contrast, **17** showed weak fluorescence with a low quantum yield (Φ_f_ = 0.11, 26‐fold), where as an appreciable fluorescence enhancement was observed upon **18** with removal of the benzoyl group (Φ_f_ = 0.21, 60‐fold) in 5% dimethyl sulfoxide (DMSO) water. As a result, probes bearing a phthalimide group (**14** and **19)** were found to be the most promising fluorescent architectures for further UV‐ activatable fluorogenic labeling investigation.

For further identifying the characterization and fluorescent mechanism of the above fluorescent probes, the density‐functional theory (DFT) and time‐dependent density functional theory (TD‐DFT) methods in Gaussian09 package^20^ were carried out with the structure optimization, frequency analysis, and fluorescence calculation. As shown in Figure 3B,C, the calculation results revealed that all the fluorescent products **14**, **17**, **18**, and **19** have a local maximum absorption wavelength of around 400 nm and a peak emission wavelength ranging from 500 to 550 nm. Notably, this structural type of probes, such as **14** has two absorption peaks, one is at 265 nm which leads to the fluorescence emission with a low quantum yield (Φ_f_ = 0.29), while the other one is at 419 nm which produces a high quantum yield (Φ_f_ = 0.65). Due to the absorption peak of 265 nm, the enhancement of fluorescence intensity of **14** could be observed in experiment when **14** was further irradiated by 254 nm. The calculating absorption and emission spectrum are well matched with the experimental results as shown in Figure 3A and Figure S14 in the Supporting Information. Especially, similar to the experimental fluorescence intensity, compounds **14** and **19** in computationally also exhibited a stronger fluorescence with a higher quantum yield (Φ_f_ = 0.65 and Φ_f_ = 0.75, respectively) than **17** and **18** with a lower quantum yield (Φ_f_ = 0.13 and Φ_f_ = 0.27, respectively).

On the basis of the experimental mechanism results described above and the Figure S16 in the Supporting Information, the results of identification experiments for product **14** by ^1^H NMR and liquid chromatograph‐mass spectrometer (LC‐MS) showed there were no structure changes after 254 nm UV‐irradiation. It means the fluorescence enhanced by UV radiation was not induced by structure changes. In Figure S14 in the Supporting Information, the DFT calculation results showed that the probe **14** has a absorption peak at 265 nm, it leads to the fluorescence emission with a low quantum yield (Φ_f_ = 0.29). Due to the one absorption peak of 265 nm, the fluorescence intensity of **14** (two absorption peaks) could be further enhanced by 254 nm irradiation. Combination of stability experimental evidences of **14** after 254 nm UV light irradiation and DFT calculation results, we deem the explanation for fluorescence enhancement phenomenon by 254 nm irradiation is different from traditional photoactivation, the “electron transition state change” is suggested as the reasonable mechanism. We consider that the fluorescence can be enhanced by UV radiation maybe invited by the ground state change: 1) There are several energy levels in the ground state and excited state, respectively. The electrons excited by 254 and 370 nm are in different ground state energy level, and those excited electrons are also in different excited state energy levels. As shown in Figure (the red solid arrow and blue solid arrow); 2) When electrons excited by 254 nm return to the ground state, they may return to the ground state energy level corresponding to 370 nm (as shown in blue dotted arrow). So after 254 nm UV light irradiation, fluorescence can be enhanced at 370 nm. It means after 254 nm UV light irradiation, the fluorescence is much stronger than which is directly excited at 370 nm.

Since these computation results were able to reproduce the experimental fluorescence results, the above DFT and TD‐DFT calculation methods were also employed to explain the principle of fluorescence emission. The highest occupied molecular orbital–lowest unoccupied molecular orbital (HOMO–LUMO) molecular orbitals of compounds **8, 14, 17, 18,** and **19** were compared, which were shown in Figure 3D and Figure S15 in the Supporting Information. The calculation results revealed that the HOMO–LUMO orbitals were mainly scattered among the phthalimide part of **14**, **17**, **18**, and **19**; however, for compound **8**, only LUMO orbital was dispersed in the phthalimide part, and its HOMO orbital was yet mostly focused on the nitroso group. Obviously, this phenomenon was caused by the electron‐withdrawing effect of nitroso group. Due to the delocalization of HOMO–LUMO on the phthalimide group, the electron of compound **14**, **17**, **18**, and **19** could jump from HOMO to LUMO. Therefore, a wavelength around 400 nm was absorbed by **14**, **17**, **18**, and **19** followed by a fluorescence emission. For compound **8**, the centralized HOMO leaded the electron hardly transiting to the delocalized LUMO. Thus, neither UV absorption around 400 nm nor any resulting fluorescence emission was found for compound **8** (see Figure 3B). The above analysis suggests that the electron delocalization degree of HOMO on the phthalimide determines the absorption and fluorescence spectrum. Based on this, nucleus independent chemical shifts (NICSs)^21^ values of phthalimide were calculated, which could quantificationally reflect the delocalization degree of electron from the HOMO of the phthalimide structure for compound **8**, **14**, **17**, **18**, and **19**. As shown in Figure 3E, the electron delocalization degree of phthalimide structure for **19** was topmost, **14** took the second place, while **8** displayed the lowest. The comparison results in Figure 3E are consistent with the fluorescence intensity difference among **14**, **17**, **18**, and **19** in Figure 3B. Therefore, the highest electron delocalization degree of the phthalimide structure for **19** determines the electron transition from HOMO to LUMO (absorption) and the electron return from LUMO to HOMO (fluorescence emission).

### 2.3 Fluorogenic Labeling of Aldolase Antibody 38C2

**Figure 4:**
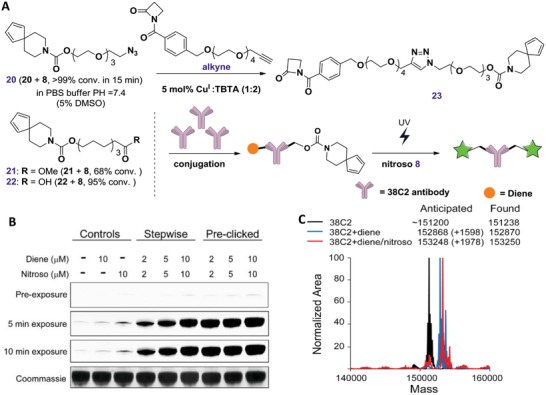
Labeling of purified 38C2. A) Synthesis of nitroso and diene compounds for 38C2 conjugation. B) UV‐induced fluorescence of labeled 38C2. Stepwise, 38C2 was labeled with β‐lactam containing diene compound first, followed by nitroso labeling; preclicked, diene, and nitroso compound was reacted prior to labeling with 38C2. C) Electrospray ionization‐mass spectrometry (ESI‐MS) characterization of labeled 38C2. The deconvoluted spectra were overlayed and plotted.

Having established the model reaction pairs for in situ generated photoactivatable fluorescence, we sought to evaluate the potential ability of this fluorogenic nitroso‐based probe for biological imaging. First, we capitalized on an aldolase catalytic antibody 38C2^22^ that can react with β‐lactam compounds^23^ to conduct selective protein labeling studies in vitro (**Figure** **4**). A bifunctional linker containing both β‐lactam and diene moieties **23** would allow selective labeling of 38C2 through a fluorogenic nitroso‐diene reaction. The synthesis was commenced by utilizing the readily available dienes with different linkers **20–22** to afford **23** (Figure 4A). Notably, when examined with nitroso **8** in PBS at 25 °C, more than 99% of **20** was converted within 15 min, indicating good solubility and reactivity of **20** under mild reaction conditions. We also found that the presence of a short polyethylene glycol (PEG) linker in dienes part is critical for the cycloaddition reactivity in PBS, as evidenced by the fact that substitution of the PEG linker with aliphatic chain (compounds **21** and **22**) diminished the reactivity (Figure S6, Supporting Information). Moreover, diene **20** was stable with different pH (Figure S7, Supporting Information) or under oxidative conditions (Figure S8, Supporting Information). Finally, **20** and β‐lactam alkyne were conjugated to obtain the designed **23** with 93% yield. The reactivity of **6** toward 38C2 antibody was validated by the competitive inhibition experiments as described,^23^ where aldolase antibody was preincubated with **6** to block the active site before the addition of the model substrate methodol (Figure S13, Supporting Information).

In order to examine the UV‐activated fluorogenic labeling of 38C2, we performed both stepwise and concerted labeling, where the former was conducted by the sequential addition of **23** (diene‐lactam pair) and **8** (nitroso pair) and the latter was performed through one single addition of the purified product from the bioorthogonal reaction between compounds **23** and **8**. We were delighted to find that fluorogenic labeling of diene‐modified 38C2 in both of these two labeling approaches proceeded effectively in an excellent UV‐exposure time‐dependent manner with very little background signals (Figure 4B). In addition, the final labeling product was also confirmed by mass spectrometry (Figure 4C). Taken together, our results suggested that this nitroso‐diene based probe was an efficient fluorogenic labeling approach with controllable fluorescence activation.

### 2.4 Fluorogenic Labeling of Herceptin Antibody and HER2‐Expressing Cells

**Figure 5:**
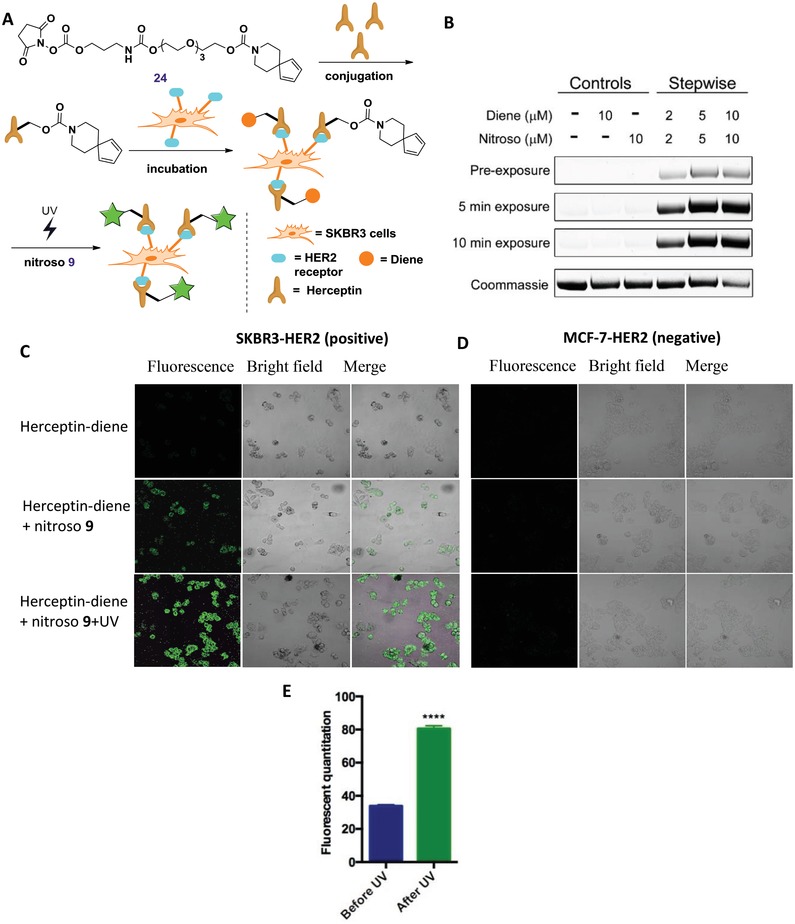
Fluorogenic labeling of HER2‐expressing cells. A) Schematic representation. B) UV‐induced fluorescence of labeled herceptin. Herceptin was labeled with diene compound first, followed by nitroso labeling. C,D) Labeling of HER2‐positive and HER2‐negative breast cancer cells with nitroso‐diene probe using HER2‐targeting antibody herceptin. E) The comparison of fluorescent quantitation for labeling SKBR3‐HER2 cell with probe before (left) and after UV (right) irradiation, **** showed before and after UV irradiation the fluorescent quantitation is significant difference (*p* < 0.0001).

Encouraged by these results, we then investigated the utility of nitroso‐diene probe for live‐cell imaging, thereby contributing to the development of new general fluorogenic labeling approach for proteins in live cells. To validate the orthogonality of these two reaction pairs in a biological setting, pretargeted SKBR‐3 (human breast cancer cell line, overexpression of HER2 receptor) and MCF‐7 (human breast cancer cell line, negative expression of HER2 receptor) labeling studies were performed (**Figure** **5**A).^24^ First, to further confirm the effectiveness of the proposed fluorogenic labeling proteins approach, the HER2 antibody Herceptin was labeled successfully by using the nitroso (**9**)–diene (**24**) probe pair according to the experimental stepwise process described above in labeling 38C2 antibody, and our obtained results were consistent with the observation in labeling 38C2 experiment (Figure 5B). Subsequently, in order to exploit the HER2 receptor to test the orthogonality of our nitroso‐diene probe pair, a selective labeling cells study was conducted. The diene (**24**)‐modified herceptin antibody was first incubated with SKBR‐3 and MCF‐7 cells respectively, and then the excess antibody was removed by wash. Subsequently, nitroso pair (**9**) was added to antibody‐labeled cells. Finally, the resulted cells were fixed and imaged (Figure 5C,D). As shown in Figure 5C, the bright cell‐surface fluorescence was clearly observed for the labeled SKBR‐3 cells, and its fluorescence could be enhanced markedly by UV‐irradiation, whereas no fluorescence was observed in MCF‐7 cells. The above results indicated that the nitroso tagging was highly specific for the cellular targets of interest. Notably, both of the diene and nitroso compounds were shown to be harmless to living cells. Thus this set of experiments confirmed the orthogonality of the diene and nitroso reaction pairs in a biological environment.

### 2.5 Fluorogenic Labeling of Scaffold Protein Using Phalloidin Derivatives

**Figure 6:**
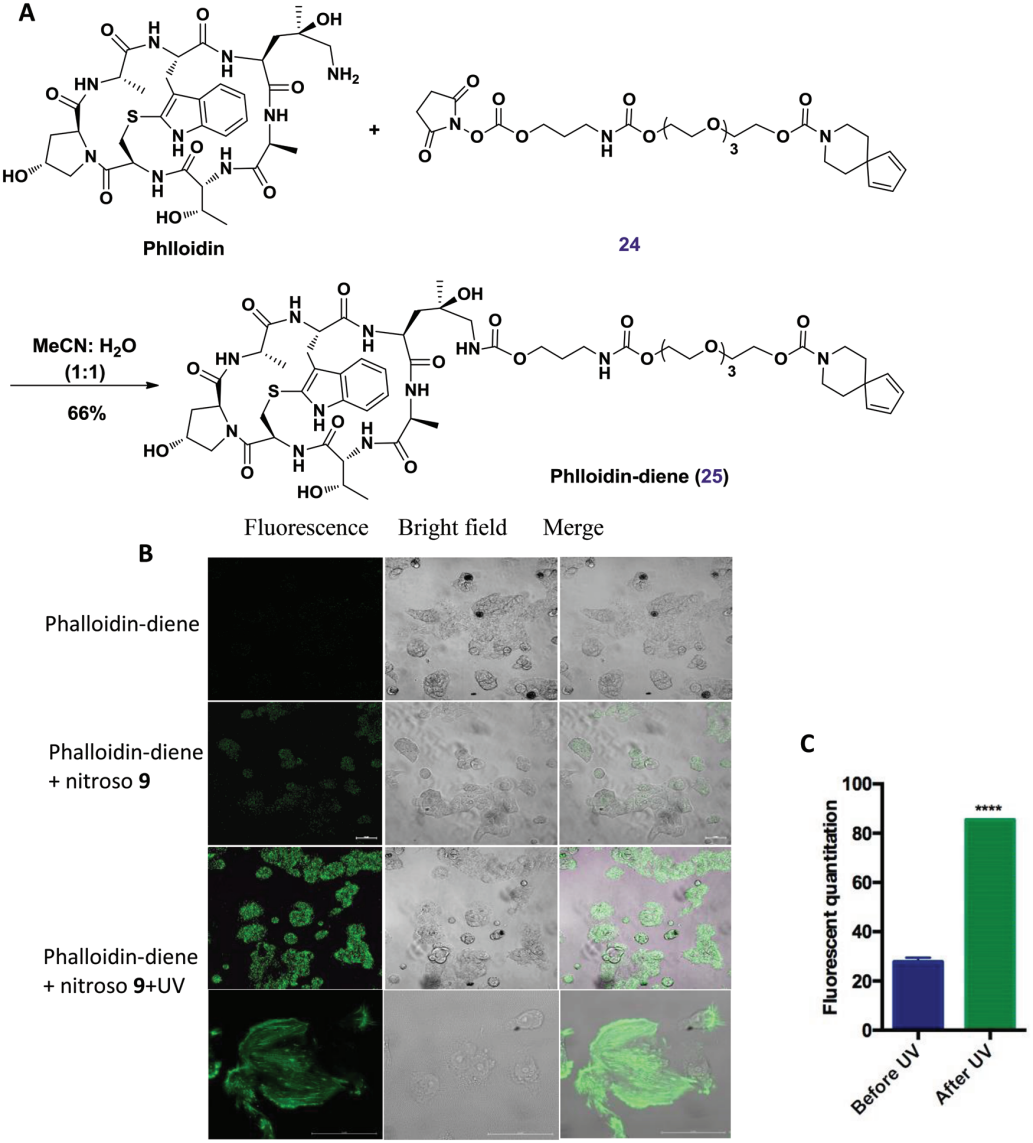
Fluorogenic labeling of scaffold protein. A) Synthesis of diene‐bearing phalloidin. B) Labeling of MCF‐7 cells with nitroso‐diene probe. C) The comparison of fluorescent quantitation for labeling MCF7 cells with probe before (left) and after UV (right) irradiation. **** showed before and after UV irradiation the fluorescent quantitation is significant difference (*p* < 0.0001).

These observations encouraged us to further investigate the feasibility of this optimized labeling pairs to image intracellular proteins in more extensive live cell studies. To gain a better understanding of the process of this conceptually new fluorogenic labeling system, the experiment of labeling F‐actin scaffold protein by using phalloidin derivatives was performed.^25^ As illustrated in **Figure** **6**A, the diene‐phalloidin derivative **25** was first synthesized and then subjected to incubation with MCF‐7 cells. Subsequently, the labeled cells were washed to remove excess compound, followed by the addition of the nitroso compound **9**. Consistent with the above results, the fluorescence was first observed upon the addition of **9**, and after further UV‐irradiation the bright green fluorescence was clearly distributed throughout the cytoplasm as shown in Figure 6B, again indicating our diene‐nitroso labeling was highly specific for the cellular targets of interest.

### 2.6 In Vivo Fluorescence Imaging

**Figure 7:**
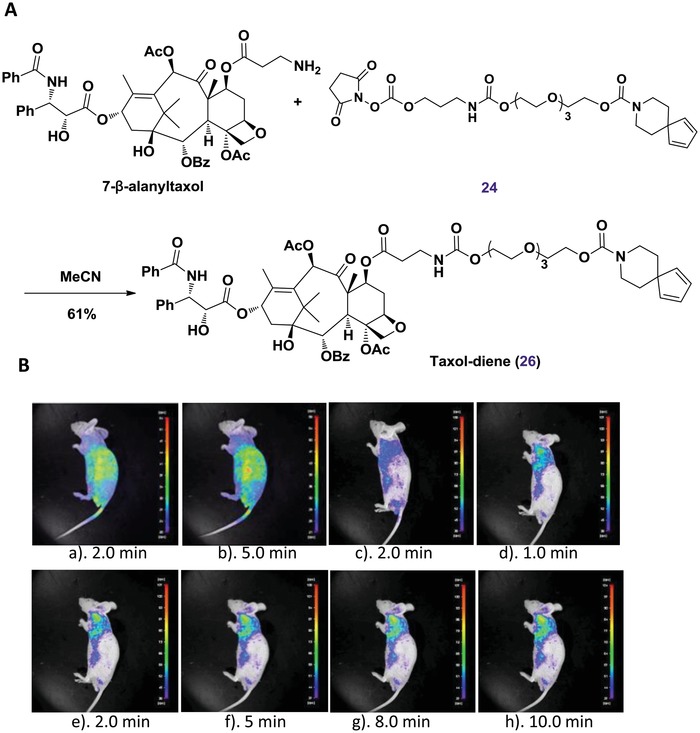
In vivo fluorescence imaging. A) Synthesis of diene‐bearing taxol. B) NIR images of living ZR‐75‐1 breast tumor model mice after tumor local injection of nitroso‐diene probe at different time points: a,b) Control fluorescence imaging for injection of fluorogenic probe pairs: the nontargeted diene (**20**) and **9** at 2.0 and 5.0 min points. c) Control fluorescence imaging for injection of taxol‐diene (**26**) at 2.0 min point. d–h) Fluorescence imaging for injection of fluorogenic probe pairs: taxol‐diene (**26**) and **9** at 1.0, 2.0, 5.0, 8.0, and 10.0 min points.

Effective imaging of bioactive small molecules in living animals could significantly facilitate further clinical diagnosis and treatment. Therefore, the effectiveness of in vivo fluorescence imaging based on nitroso‐diene probe was evaluated using a ZR‐75‐1 breast tumor model in BALB/c nu/nu mice. As a commonly used drug in cancer selective chemotherapy, taxol was chosen for this study.^26^ Taxol‐diene (**26**) was prepared smoothly through coupling **24** with taxol derivative 7‐β‐alanyltaxol^27^ (**Figure** **7**A). A chemical pretargeted approach would allow direct tagging and tracking of bioactive small molecules without severe perturbation of their in vivo properties. Subsequently, the fluorogenic probe pairs taxol‐diene (**26**, 0.15 µmol) and nitroso **9** (0.45 µmol) were injected locally into the tumors of mouse. Excitingly, compared with the control group for tumor local injection of fluorogenic probe pairs: the nontargeted diene (**20**) and **9** (a,b, Figure 7B), and taxol‐diene (c, Figure 7B), tumors were visibly distinct by fluorescence NIR‐imaging at 1.0, 2.0, 5.0, 8.0, and 10.0 min points in the absence of further UV‐irradiation (d–h, Figure 7B). The rapid blood clearance and excretion typical of most small molecules necessitates that the clickable fluorescence imaging occurs within minutes. The results also demonstrated that the potential of a nontargeted preclicked nitroso‐diene probe for the noninvasive in vivo rapid fluorescence imaging of low abundance targets.

## 3 Discussion

Fluorogenic labeling is advantageous over traditional fluorescent labeling due to the improved signal‐to‐noise ratios. Fluorogenic probes typically carry profluorophores with low quantum yield, which were then converted into activated form with increased quantum yield during “bioorthogonal” reaction. The enhancement of quantum yield may vary from a couple of folds[qv: 13b] to dozens of folds;^7^, ^14^ however, fluorescence increase over 100 fold is usually difficult to achieve. This limitation hampers the detection of target molecules with high contrast over background signals such as the autofluorescence of cells. In addition, synthesis of profluorophores and tags based on current structure units, such as coumarin, fluorescein, rhodamine, BODIPY, and others, can be expensive and labor intensive. These disadvantages promoted us to explore more “bioorthogonal” fluorogenic reactions for fluorescence labeling of cellular proteins in live cell imaging studies in mammalian cells.

In this study, we discovered and capitalized on the potential fluorogenic reactivity of nitroso‐based bioorthogonal DA reaction to develop an efficient fluorogenic nitroso probe. Notably, the reaction can efficiently deliver a twisted “donor–acceptor–donor” skeleton, which was critical to the effective separation of the HOMO/LUMO electron densities and thus led to like TADF properties for photoactivatable fluorogenic probes.^18^ This feature allows selective activation of POIs in a particular cell compartment at a given time point, while maintaining the rest labeled molecules untouched. The spatial and temporal control of fluorogenic labeling may find a wide range of applications in monitoring the dynamics of POIs in live cells. In addition, we have utilized the DFT and TD‐DFT calculation methods to further identify the characterization and fluorescent mechanism of probes. The calculating results were shown to match well with the experimental results (Figure 3).

To gain a better understanding of the scope of this conceptually new fluorogenic labeling system, we then expanded our studies beyond model protocols to develop an efficient “click‐like” fluorogenic nitroso‐diene pair probe for a new general, controllable fluorogenic protein labeling in live cells. As a proof‐of‐concept study, our results strongly indicate that the optimized nitroso‐diene probe can provide a powerful tool for fluorogenic proteins labeling in live cells (Figures 4, 5, 6). These results provided additional evidence that the fluorescence imaging was indeed generated by the specific nitroso‐DA cycloaddition, and this viable fluorogenic labeling approach that can be tuned by UV irradiation in an excellent UV‐exposure time‐dependent manner with virtually no background labeling signals. The highly efficient, specific, and bright protein fluorogenic labeling using this nitroso‐diene “click‐like” chemistry demonstrates the potential of this method for visualizing various cellular processes. Moreover, we also demonstrated the use of this nitroso‐diene bioorthogonal chemical reaction between two exogenous moieties in living mice for the noninvasive in vivo fluorescence imaging (Figure 7). We anticipate the method described herein will enable this new fluorogenic labeling to be used in a wide range of cellular studies in living systems.

## 4 Experimental Section

The ZR‐75‐1 breast tumor model in BALB/c nu/nu mice for the in vivo imaging experiment in this study was obtained from the Laboratory Animal Center of Sun Yat‐sen University, and was approved by the Laboratory Animals Use Committee of Sun Yat‐sen University (license: SYXK (Canton) 2016‐0112 and SCXK (Canton) 2016‐0029). The mice met the guidelines of the Care and Use of Laboratory Animals as adopted and promulgated by the U.S. National Institutes of Health Animal Care. The general procedures of the synthesis of nitroso and diene compounds, measurement of the spectral properties of cycloaddition products, cell culture experiments, antibodies labeling can be found in the Supplementary Information.

## Conflict of Interest

The authors declare no conflict of interest.

## Supporting information

Supplementary

## References

[1] a) M.‐N. Zhou , C.‐S. Delaveris , J.‐R. Kramer , J.‐A. Kenkel , E.‐G. Engleman , C. R. Bertozzi , Angew. Chem., Int. Ed. 2018, 57, 3137; 10.1002/anie.201713075 PMC5842139 29370452

[2] a) B. N. G. Giepmans , S. R. Adams , M. H. Ellisman , R. Y. Tsien , Science 2006, 312, 217; 16614209 10.1126/science.1124618

[3] a) G. V. Los , L. P. Encell , M. G. McDougall , D. D. Hartzell , N. Karassina , C. Zimprich , M. G. Wood , R. Learish , R. F. Ohana , M. Urh , D. Simpson , J. Mendez , K. Zimmerman , P. Otto , G. Vidugiris , J. Zhu , A. Darzins , D. H. Klaubert , R. F. Bulleit , K. V. Wood , ACS Chem. Biol. 2008, 3, 373; 18533659 10.1021/cb800025k

[4] W. P. Janzen , Chem. Biol. 2014, 21, 1162. 25237860 10.1016/j.chembiol.2014.07.015

[5] a) C. R. Bertozzi , Acc. Chem. Rev. 2011, 44, 651; 10.1021/ar200193f PMC4408923 21928847

[6] a) Z.‐Q. Li , D.‐Y. Wang , L. Li , S.‐J. Pan , Z.‐K. Na , C. Y. J. Tan , S.‐Q. Yao , J. Am. Chem. Soc. 2014, 136, 9990; 24972113 10.1021/ja502780z

[7] a) H. X. Wu , N. K. Devaraj , Acc. Chem. Res. 2018, 51, 1249; 29638113 10.1021/acs.accounts.8b00062 PMC6225996

[8] a) C. Le Droumaguet , C. Wang , Q. Wang , Chem. Soc. Rev. 2010, 39, 1233; 20309483 10.1039/b901975h

[9] a) K. Wang , F. Friscourt , C.‐F. Dai , L.‐F. Wang , Y.‐Q. Zheng , G.‐J. Boons , S. Wang , B.‐H. Wang , Bioorg. Med. Chem. Lett. 2016, 26, 1651; 26944613 10.1016/j.bmcl.2016.02.069 PMC4797929

[10] a) M. J. Hangauer , C. R. Bertozzi , Angew. Chem., Int. Ed. 2008, 47, 2394; 10.1002/anie.200704847 PMC2446402 18306205

[11] a) K. Lang , L. Davis , S. Wallace , M. Mahesh , D. J. Cox , M. L. Blackman , J. M. Fox , J. W. Chin , J. Am. Chem. Soc. 2012, 134, 10317; 22694658 10.1021/ja302832g PMC3687367

[12] a) A. Wieczorek , P. Werther , J. Euchner , R. Wombacher , Chem. Sci. 2017, 8, 1506; 28572909 10.1039/c6sc03879d PMC5452268

[13] a) Z. P. Yu , L. Y. Ho , Q. Lin , J. Am. Chem. Soc. 2011, 133, 11912; 21736329 10.1021/ja204758c PMC3150427

[14] P. Shieh , M. J. Hangauer , C. R. Bertozzi , J. Am. Chem. Soc. 2012, 134, 17428. 23025473 10.1021/ja308203h PMC3596100

[15] J.‐J. Shie , Y.‐C. Liu , Y.‐M. Lee , C. Lim , J.‐M. Fang , C.‐H. Wong , J. Am. Chem. Soc. 2014, 136, 9953. 24955871 10.1021/ja5010174

[16] a) S. Mizukami , S. Watanabe , Y. Akimoto , K. Kikuchi , J. Am. Chem. Soc. 2012, 134, 1623; 22224915 10.1021/ja208290f

[17] a) A. V. Samoshin , C. J. Hawker , J. R. de Alaniz , ACS Macro Lett. 2014, 3, 753; 10.1021/mz500348y 35590694

[18] M. Li , Y.‐W. Liu , R.‐H. Duan , X.‐F. Wei , Y.‐P. Yi , Y. Wang , C.‐F. Chen , Angew. Chem., Int. Ed. 2017, 56, 8818. 10.1002/anie.201704435 28557359

[19] Z. C. Xu , K.‐H. Baek , H. N. Kim , J.‐N. Cui , X.‐H. Qian , D. R. Spring , I. Shin , J. Y. Yoon , J. Am. Chem. Soc. 2010, 132, 601. 20000765 10.1021/ja907334j

[20] a) M. J. Frisch , G. W. Trucks , H. B. Schlegel , G. E. Scuseria , M. A. Robb , J. R. Cheeseman , G. Scalmani , V. Barone , B. Mennucci , G. A. Petersson , H. Nakatsuji , M. Caricato , X. Li , H. P. Hratchian , A. F. Izmaylov , J. Bloino , G. Zheng , J. L. Sonnenberg , M. Hada , M. Ehara , K. Toyota , R. Fukuda , J. Hasegawa , M. Ishida , T. Nakajima , Y. Honda , O. Kitao , H. Nakai , T. Vreven , J. A. Montgomery Jr. , J. E. Peralta , F. Ogliaro , M. Bearpark , J. J. Heyd , E. Brothers , K. N. Kudin , V. N. Staroverov , R. Kobayashi , J. Normand , K. Raghavachari , A. Rendell , J. C. Burant , S. S. Iyengar , J. Tomasi , M. Cossi , N. Rega , J. M. Millam , M. Klene , J. E. Knox , J. B. Cross , V. Bakken , C. Adamo , J. Jaramillo , R. Gomperts , R. E. Stratmann , O. Yazyev , A. J. Austin , R. Cammi , C. Pomelli , J. W. Ochterski , R. L. Martin , K. Morokuma , V. G. Zakrzewski , G. A. Voth , P. Salvador , J. J. Dannenberg , S. Dapprich , A. D. Daniels , Ö. Farkas , J. B. Foresman , J. V. Ortiz , J. Cioslowski , D. J. Fox , Gaussian 09, Revision A.02, Gaussian, Inc., Wallingford, CT 2009;

[21] a) Z. F. Chen , C. S. Wannere , C. Corminboeuf , R. Puchta , P. von Ragué Schleyer , Chem. Rev. 2005, 105, 3842; 16218569 10.1021/cr030088+

[22] J. Wagner , R. A. Lerner , C. F. Barbas III , Science 1995, 270, 1797. 8525368 10.1126/science.270.5243.1797

[23] J. I. Gavrilyuk , U. Wuellner , C. F. Barbas III , Bioorg. Med. Chem. Lett. 2009, 19, 1421. 19181522 10.1016/j.bmcl.2009.01.028 PMC2688461

[24] M. R. Karver , R. Weissleder , S. A. Hilderbrand , Angew. Chem., Int. Ed. 2012, 51, 920. 10.1002/anie.201104389 PMC3304098 22162316

[25] L.‐G. Milroy , S. Rizzo , A. Calderon , B. Ellinger , S. Erdmann , J. Mondry , P. Verveer , P. Bastiaens , H. Waldmann , L. Dehmelt , H.‐D. Arndt , J. Am. Chem. Soc. 2012, 134, 8480. 22475347 10.1021/ja211708z

[26] N. K. Devaraj , S. Hilderbrand , R. Upadhyay , R. Mazitschek , R. Weissleder , Angew. Chem., Int. Ed. 2010, 49, 2869. 10.1002/anie.200906120 PMC3433403 20306505

[27] R. K. Guy , Z. A. Scott , R. D. Sloboda , K. C. Nicolaou , Chem. Biol. 1996, 3, 1021. 9000007 10.1016/s1074-5521(96)90168-4

