# Supplementary material for: Photoactivatable Fluorogenic Labeling via Turn‐On “Click‐Like” Nitroso‐Diene Bioorthogonal Reaction
Source: Adv Sci (Weinh). 2019 May 2;6(13):1802039. doi: 10.1002/advs.201802039 (PMC6662066; doi:10.1002/advs.201802039)
Supplement: Supplementary file 1 — Supplementary [file ADVS-6-1802039-s001.pdf]

Copyright WILEY-VCH Verlag GmbH & Co. KGaA, 69469 Weinheim, Germany, 2019.

ADVANCED SCIENCE
Open Access

Supporting Information

for *Adv. Sci.,* DOI: 10.1002/advs.201802039

Photoactivatable Fluorogenic Labeling via Turn-On “Click-Like” Nitroso-Diene Bioorthogonal Reaction

Bai Li, Xian-Hao Zhou, Peng-Yu Yang, Liping Zhu, Yuan Zhong, Zhengjun Cai, Biao Jiang, Xiaoqing Cai, Jia Liu, and Xianxing Jiang**

Copyright WILEY-VCH Verlag GmbH & Co. KGaA, 69469 Weinheim, Germany, 2016

Supplementary Information

Photo-activatable Fluorogenic Labeling via Turn-On "Click-Like" Nitroso-Diene Bioorthogonal Reaction

Bai Li, *Xian-Hao Zhou*, *Peng-Yu Yang*, *Liping Zhu*, *Yuan Zhong*, *Zhengjun Cai*, *Biao Jiang*, *Xiaoqing Cai*, *Jia Liu**, and *Xianxing Jiang**

*To whom correspondence may be addressed. Email: jiangxx5@mail.sysu.edu.cn, and liujia@shanghaitech.edu.cn

Table of Contents

1. Materials and Methods for Labeling and Imaging

2. Supplementary Figures

Supplementary Figure S1. Structures of dienes and nitroso compounds

Supplementary Figure S2. Reaction for kinetics.

Supplementary Figure S3. Absorption spectrum of compound **14** in ethanol.

Supplementary Figure S4. pH stability of nitroso compound **8**.

Supplementary Figure S5. Long-term stability of nitroso compound **8**.

Supplementary Figure S6. Exploring the effectiveness and reactivity for the aliphatic chain linked dienes in aqueous media.

Supplementary Figure S7. PH stability of diene **20**.

Supplementary Figure S8. Stability of diene **20** under oxidative conditions.

Supplementary Figure S9. Stability and selectivity studies of **20** in the presence of reactive amino acids.

Supplementary Figure S10. Selectivity evaluation of the nitroso-diene fluorogenic reaction in the presence of amino acids.

Supplementary Figure S11. The stability of probe **14**.

Supplementary Figure S12. The cytotoxicity of nitroso **8** and probe **14**.

Supplementary Figure S13. Competitive inhibition experiment using compound **23**.

Supplementary Figure S14. The schematic diagram of absorption and emission spectrums for **14**.

Supplementary Figure S15. HOMO-LUMO diagrams of compounds **17** and **18**.

Supplementary Figure S16. Stability detection of product **14** by ^{1}H NMR and LC-MS after 254 nm UV light irradiation.

Supplementary Figure S17. Different power of UV lamp was used to irradiate compound **14** at 1 min, 2 min, 5 min and 10 min, respectively.

Supplementary Figure S18. The stability experiments in lysate of A431 cell.

3. Supplementary Methods for Synthesis and Measurements

3.1 General procedure for chemical synthesis

3.2 Synthesis of nitroso and diene compounds

3.3 Synthesis and characterization of 2-methyl-5-nitrosoisoindoline-1,3-dione ***(8)***

3.4 Characterization of cycloaddition products

3.5 Spectroscopic measurements

3.6 Reaction kinetic measurements

3.7 Preparation of diene for labeling

3.8 Calculation for fluorescent probes

References

4. NMR Spectra of Synthesized Compounds

1. Materials and Methods for Labeling and Imaging

1.1. Conjugation experiments with 38C2

For stepwise conjugation, 50 μM of purified murine 38C2 protein (Sigma-Aldrich) was incubated with excess diene compound **6** with molar ratios of 2:1, 5:1 and 10:1 in PBS at room temperature for 2 h. The product was desalted using Zeba desalting column (Thermo Fisher Scientific) to remove excess diene compound. In the second labeling step, nitroso compound **8** was added to 38C2 protein with molar excess of 2:1, 5:1 and 10:1 and the reaction was kept in PBS at room temperature for 2 h. For one-step labeling reaction, nitroso and diene compounds **23** and **8** were pre-clicked as described above and then incubated with 50 μM of 38C2 with molar excess of 2:1, 5:1 and 10:1 in PBS at room temperature for 2 h. The reaction products were analyzed using electrospray ionization Mass Spectrometry (ESI-MS).The obtained ion sets were deconvoluted and exported as Excel files. The ESI-MS figures were generated by plotting normalized area against apex mass.

For on-gel fluorescence, conjugation products were mixed with 2x SDS loading dye, resolved on 4-20% Tris-glycine gel and then imaged using Universal Hood II Gel Imager (BioRad). The auto-fluorescence of compounds and proteins were imaged without exposure to UV light. For UV-induced fluorescence, the gels were kept on the imager tray and exposed to UV light (400 nm wavelength) for 2.0 min and then the images were recorded.

1.2. Cell culture experiments

For labeling experiments using herceptin antibodies, HER2-positive cell line SKBR3 and HER2-negative cell line MCF-7 were used. For phalloidin labeling experiments, MCF-7 cells were used. Cells were seeded on to plates at 24 h prior to labeling. Cells were fixed with 4% paraformaldehyde. Herceptin-diene (10 μM) were incubated with cells for 1 h. Phalloidin-diene (10 μM) were cincubated with cells permeabilized with 0.5% triton X-100 for 1h. Excess diene compounds were removed by extensive wash. Nitroso compound **9** (10 μM) was added to cells. At 0.5 h after conjugation, excess **9** was removed by wash. For UV activation of fluorescence, cells were irradiated with

UV light (400 nm) for 2.0 min. Images were taken by Zeiss LSM-710 confocal microscope (excitation wavelength: 405nm, detection wavelength: 480-585).

1.3.In vivo NIR fluorescence imaging

Six weeks old BALB/c nu/nu mice were injected with 2 × 106 ZR-75-1 cells in a total volume of 100.0 μL PBS per day for 3 weeks. The cell preparations were given subcutaneously on the right flank of animals. NIR imaging was performed until tumor burden reached 1.0 cm at the longest dimension (totally 3 weeks). Before imaging, mice were anesthetized with pentobarbital sodium，60mg/kg. Different doses of drug were in situ injection. Fluorescence imaging for injection of fluorogenic probe pairs: taxol-diene (**26**) and **9** at 1.0 min, 2.0 min, 5.0 min, 8.0 min and 10.0 min points. The images were collected on an IVIS spectrum optical imaging system (Berthold Technologies). General settings for collecting epi-fluoroscent images were as follows: Epi-illumination, λ_{em}=520, λ_{ex}=475, Camera Gain: Low, Camera Readout: Fast, Illumination [%]:10, acquisition time = 0.1 s.

2. Supplementary Figures

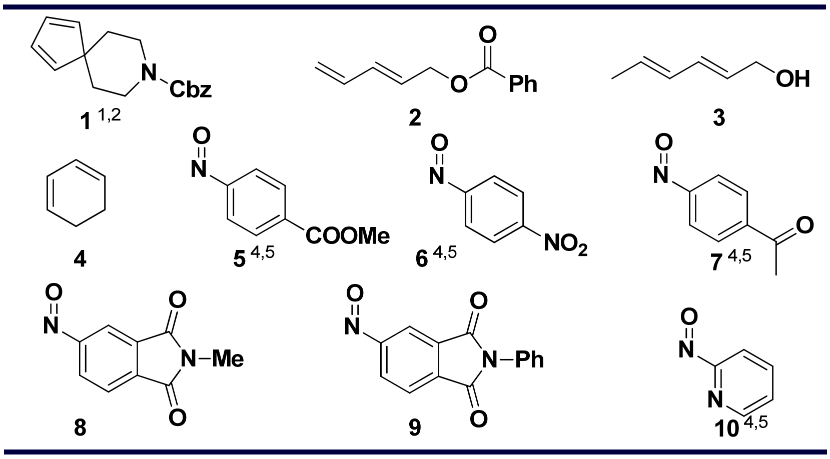

Supplementary Figure S1. **Structures of dienes and nitroso compounds.**

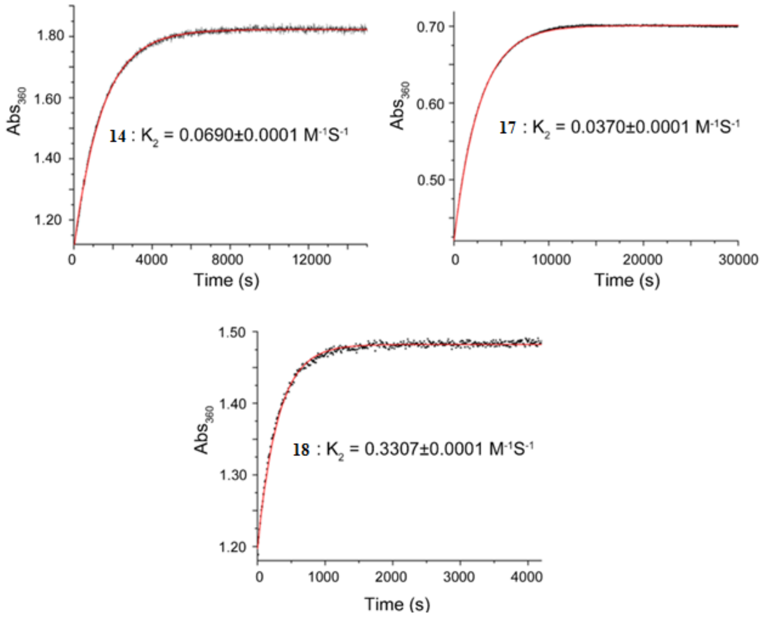

Supplementary Figure S2. **Reaction kinetics of nitroso 8 with dienes 1**, **2** and **3.** Reaction kinetics were determined by reacting 1.0 mM dienophile (**8**) with an excess of 10.0 mM dienes (**1**, **2** and **3**) in 80% DMF, 20% PBS (pH 7.4) at 25°C. Data was collected under pseudo first-order conditions (data points plotted) and fitted to a single exponential decay.

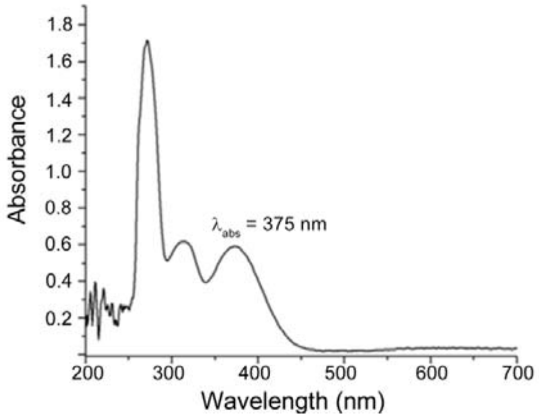

Supplementary Figure S3. **Absorption spectrum of compound 8 (100 μM) in ethanol.**

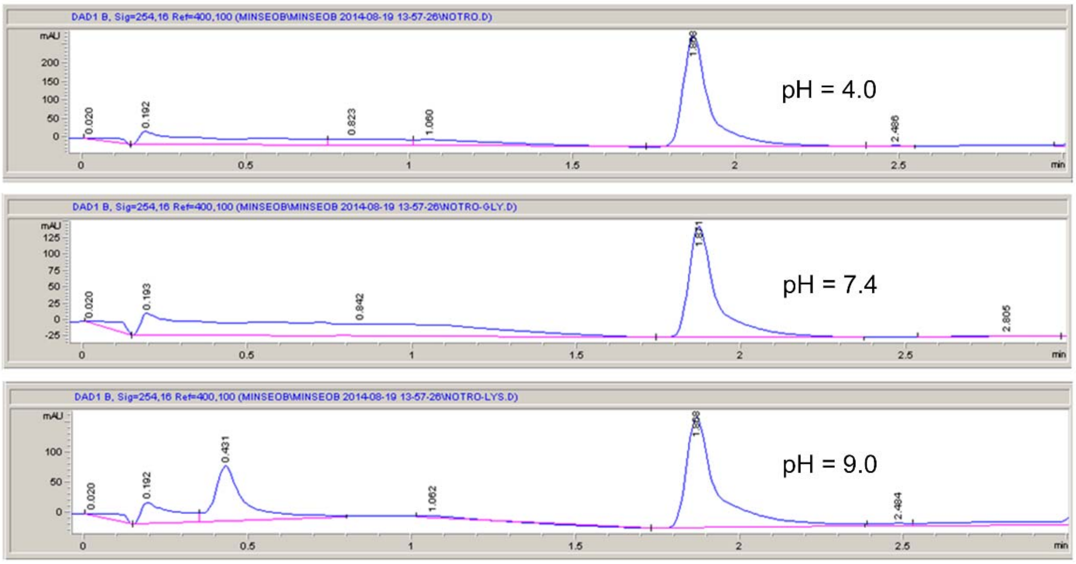

***Supplementary Figure S4*. The pH stability of nitroso 8**. Compound (100 μM) is incubated in pH 4.0, 7.4 and 8.0 sodium phosphate solution at 37 °C for 6 h and the retention of **8** is analyzed by HPLC. (HPLC condition: C_{18} column, 5 μm, 4.5*150 mm. Elution is water (0.1% TFA) /methanol = 5: 95, flow rate = 0.8 mL min^{-1}).

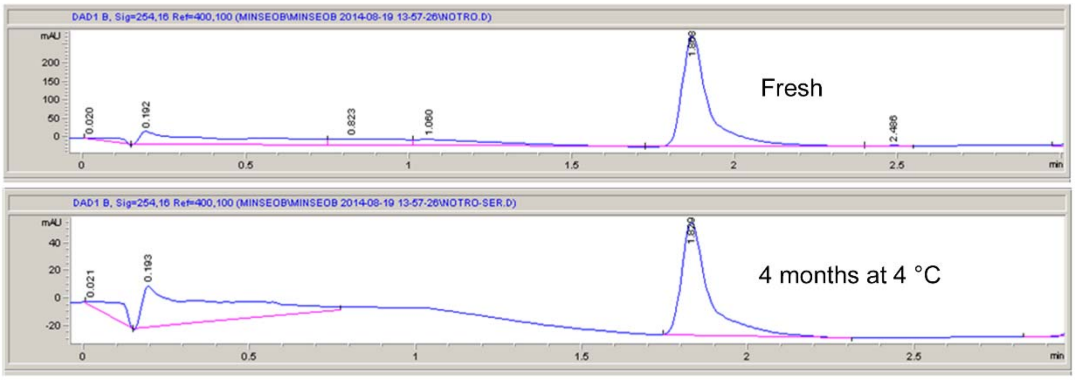

***Supplementary Figure S5*. Long-term stability of nitroso 8.** Compound is analyzed by HPLC by 4 month storage at 4 ºC as powder. (HPLC condition: C_{18} column, 5 μm, 4.5*150 mm. Elution is water (1% TFA) /methanol = 5: 95, flow rate = 0.8 mL min^{-1})

**Dienes**	**T**(min)	**Conv.**(%)
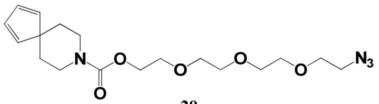 **20**	30	>99
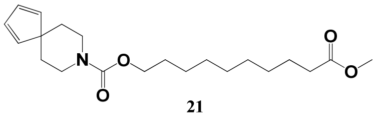 **21**	30	68
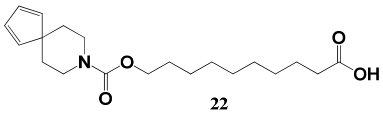 **22**	30	95

***Supplementary Figure S6*. The effect of aliphatic side chain of diene linker on the nitroso-DA reaction.** Dienes with different side chain moieties (1 mM) are reacted with nitroso compound **8** (2.0 mM) in pH 7.4 PBS buffer with 5% DMSO at room temperature. The progress of the reaction is monitored by LCMS at 30 min after initiation of the reaction.

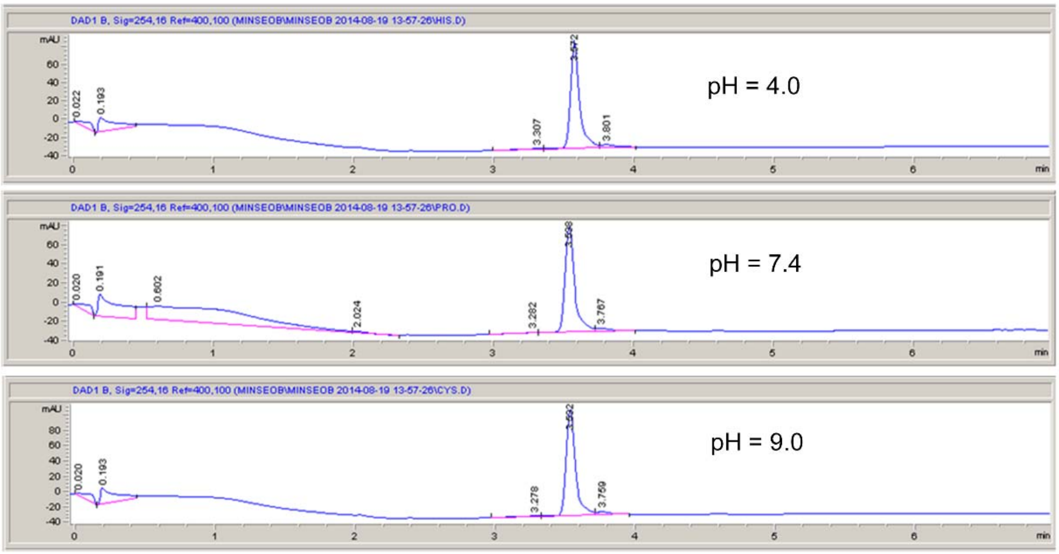

***Supplementary Figure S7*. pH stability of the diene linker.** Compound **20** is incubated in pH 4.0, 7.4 and 9.0 sodium phosphate solution at 37 °C for 24 h and the retention of **20** is analyzed by HPLC. (HPLC condition: C_{18} column, 5 µm, 4.5*150 mm. Elution is water (1% TFA) /methanol = 5: 95, flow rate = 0.8 mL min^{-1})

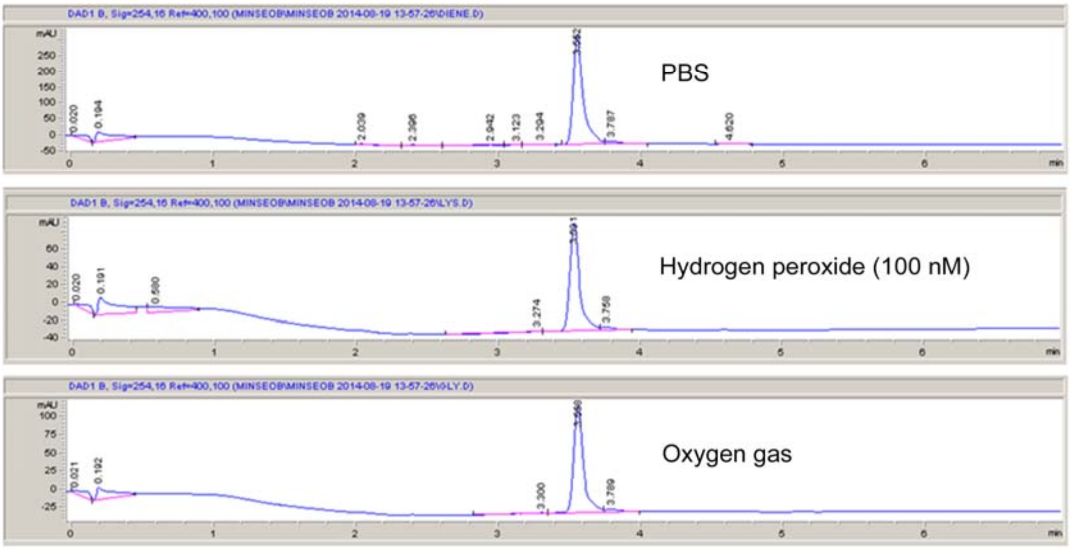

***Supplementary Figure S8*. Stability of the diene linker under oxidative conditions.** Compound **20** is incubated with 100 nM of hydrogen peroxide or pure oxygen in PBS (with 3% DMSO) at 37 °C for 6 h and the retention of **20** is analyzed by HPLC.

(HPLC condition: C_{18} column, 5 μm, 4.5*150 mm. Elution is water (1% TFA) /methanol = 5: 95, flow rate = 0.8 mL min^{-1})

#

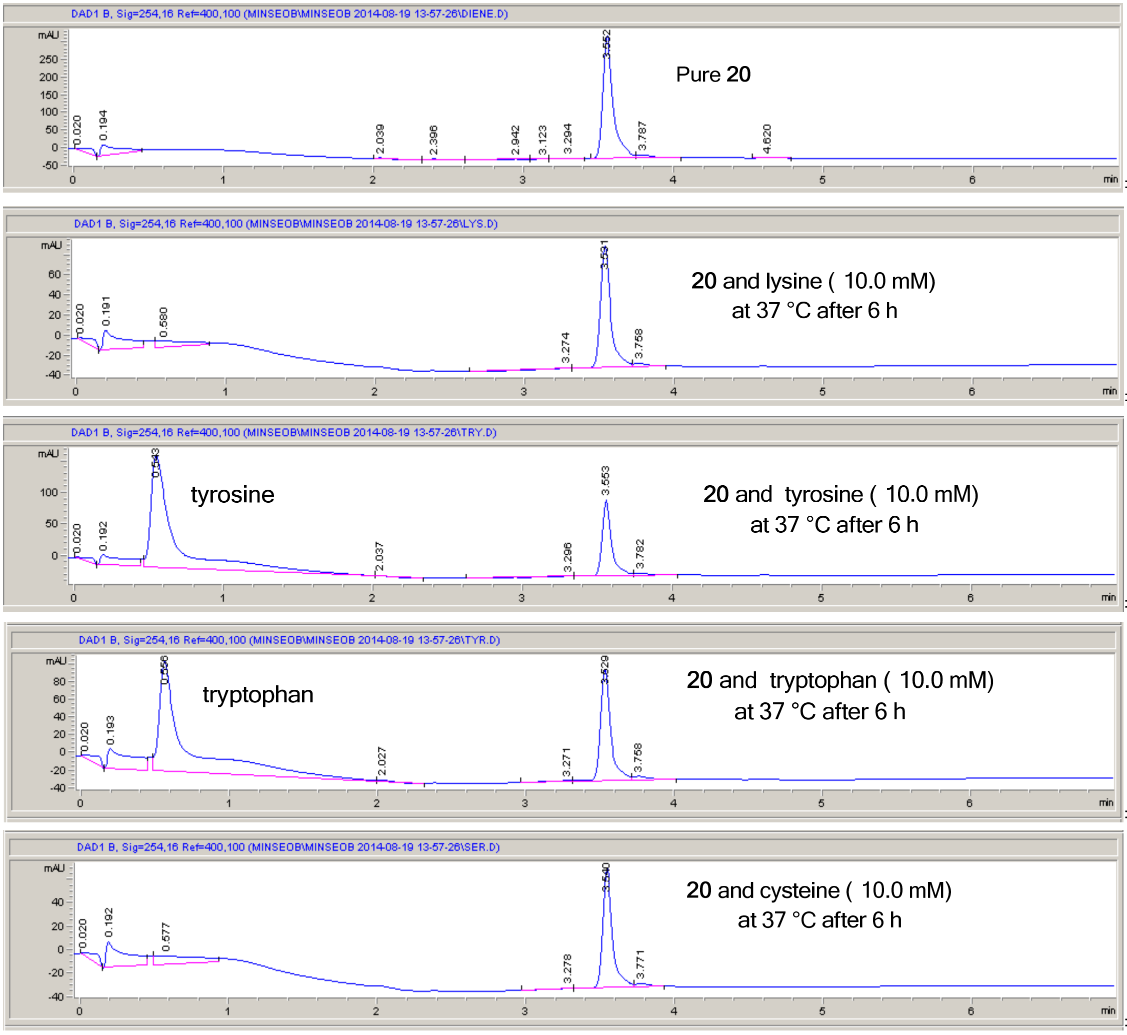

***Supplementary Figure S9*. Stability and selectivity studies of 20 in the presence of reactive amino acids.** To further analyze the stability and selectivity of diene **20** in the presence of reactive amino acids like lysine, tyrosine, tryptophan and cysteine, **20** (10.0 mM) was treated with amino acids (10.0 mM) at 37 °C in PBS buffer PH= 7.4 (3% DMSO). The resulting solutions were monitored via HPLC after 6 h. (HPLC condition: C_{18} column, 5 μm, 4.5*150 mm. Elution is water (1% TFA) /methanol = 5: 95, flow rate = 0.8 mL min^{-1})

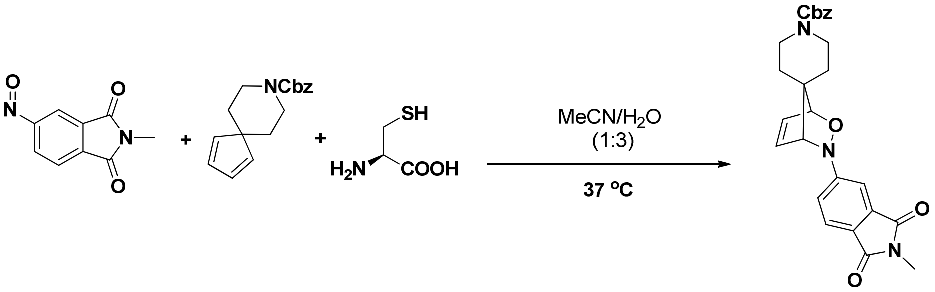

8 **1** **14**

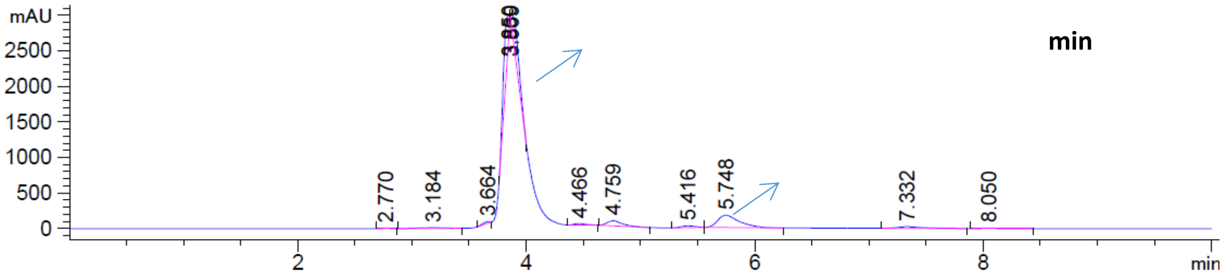
min
mAU
2.770
3.184
3.664
4.466
4.759
5.416
5.748
7.332
8.050
min

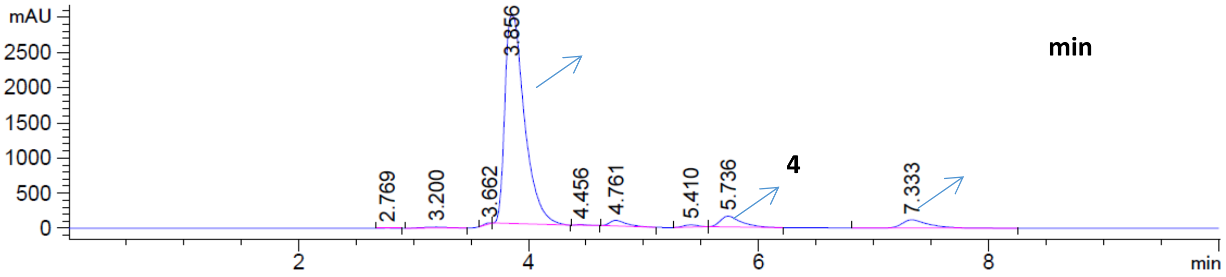
min
mAU
2.769
3.200
3.662
3.856
4.456
4.761
5.410
5.736
4
7.333
min

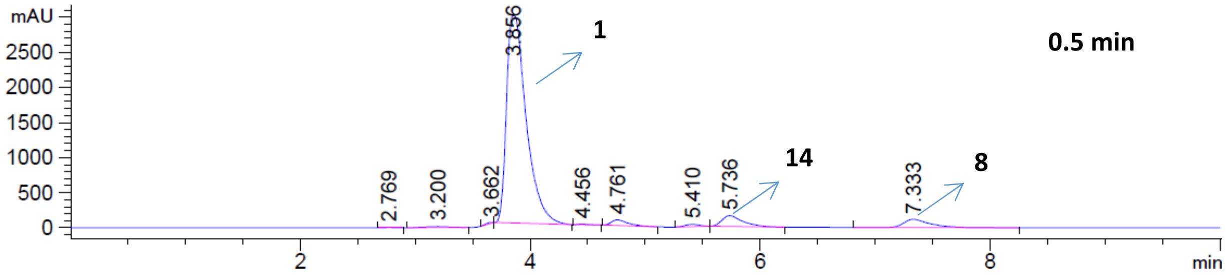
0.5 min
mAU
1
14
8
2.769
3.200
3.662
3.856
4.456
4.761
5.410
5.736
7.333
min

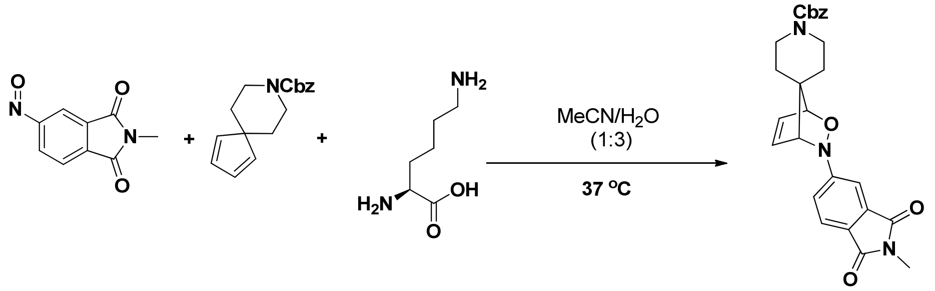
NCbz
NH2
OH
H2N
MeCN/H2O
(1:3)
37 ºC
Cbz
N
O

8 1 14

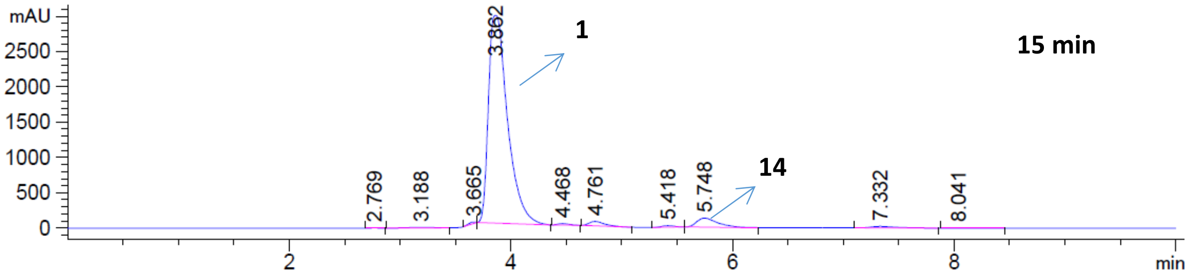
15 min
mAU
1
14
2.769
3.188
3.665
3.862
4.468
4.761
5.418
5.748
7.332
8.041
min

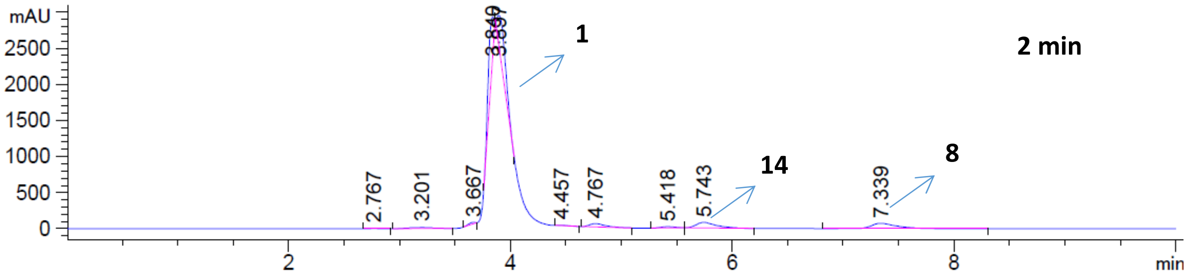
2 min
mAU
1
14
8
2.767
3.201
3.667
4.457
4.767
5.418
5.743
7.339
min

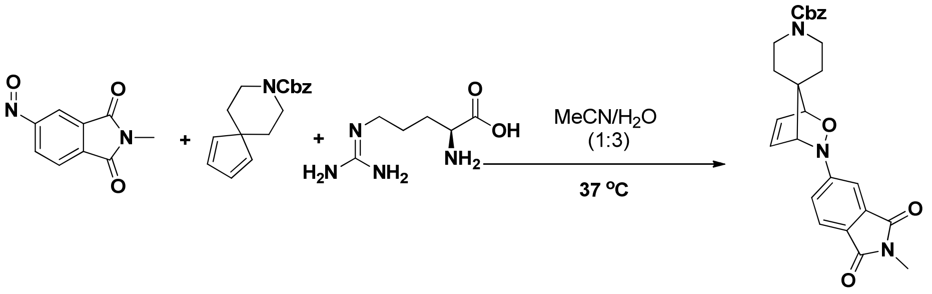
NCbz
Cbz
MeCN/H2O
(1:3)
37 °C
OH
NH2
H2N

8 1 14

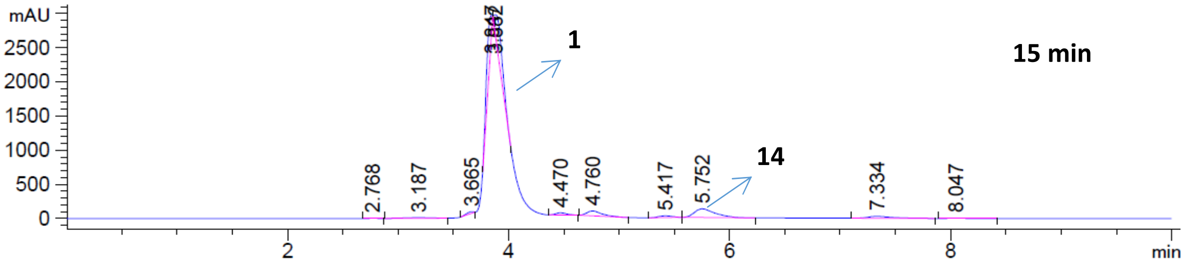
mAU
15 min
1
14
2.768
3.187
3.665
4.470
4.760
5.417
5.752
7.334
8.047
min

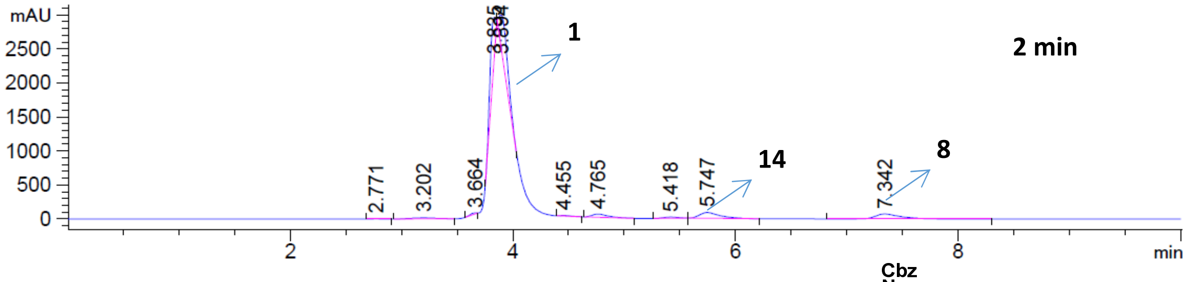
mAU
2 min
1
14
8
2.771
3.202
3.664
4.455
4.765
5.418
5.747
7.342
min

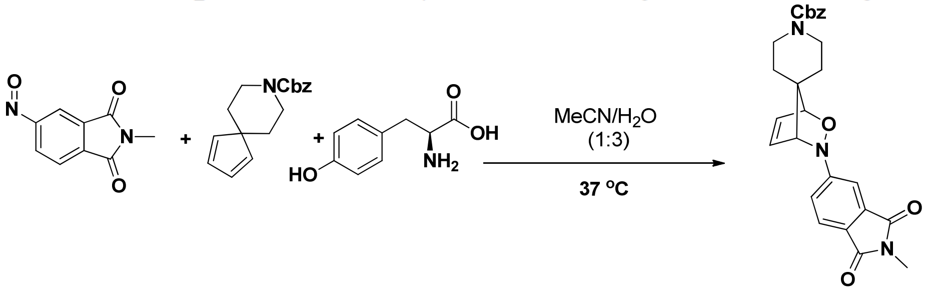
NCbz
Cbz
MeCN/H2O
(1:3)
37 °C
OH
NH2
HO

8 1 14

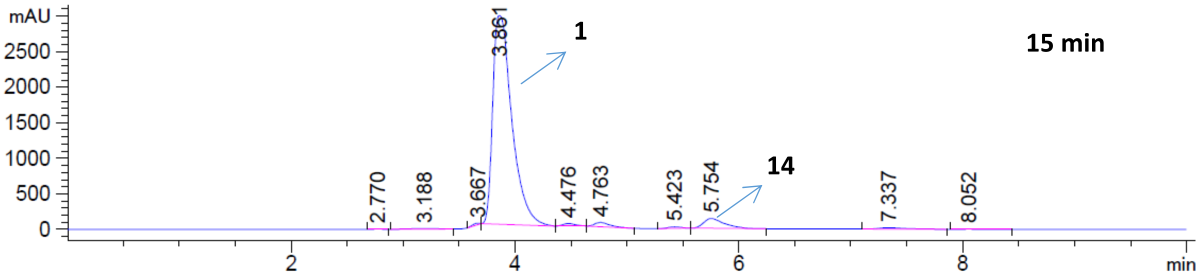
mAU
15 min
1
14
2.770
3.188
3.667
3.861
4.476
4.763
5.423
5.754
7.337
8.052
min

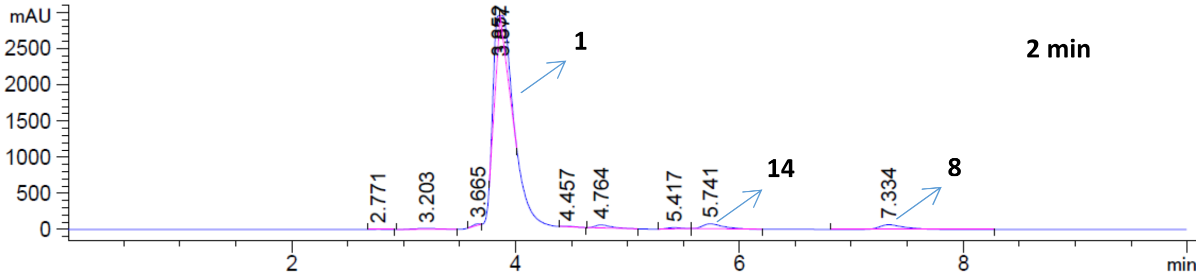
mAU
2 min
1
14
8
2.771
3.203
3.665
4.457
4.764
5.417
5.741
7.334
min

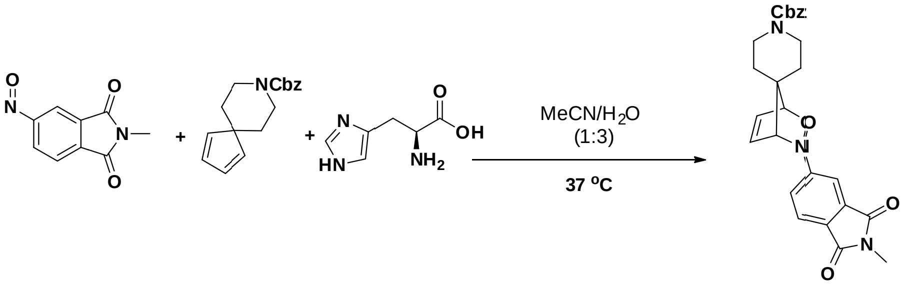

8 1 14

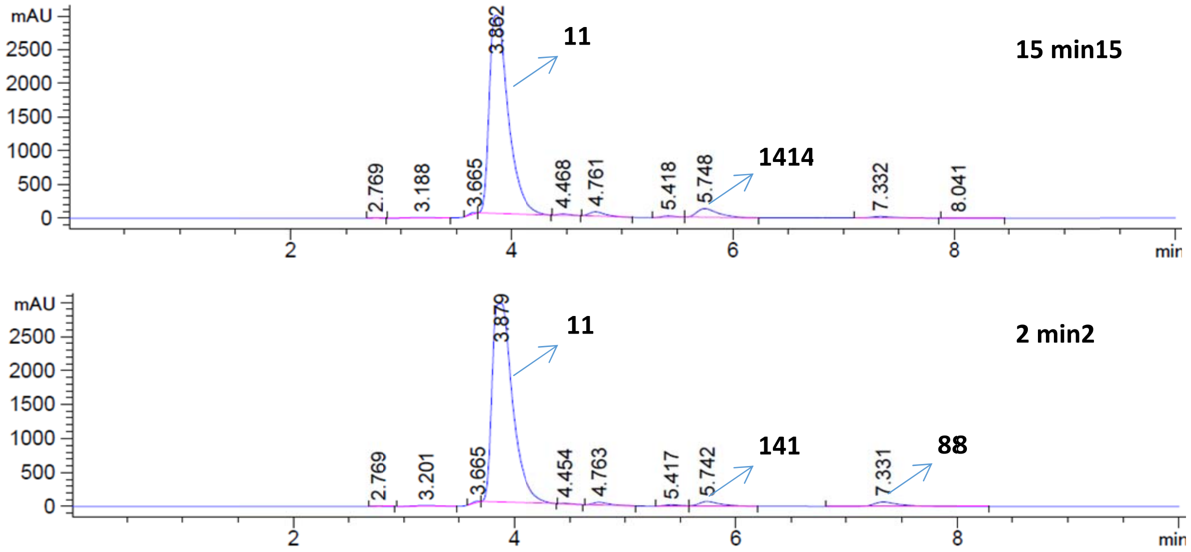

***Supplementary Figure S10*. Selectivity evaluation of the nitroso-diene fluorogenic reaction in the presence of amino acids. 1** (0.2 mM) and **8** (1.0 mM) were treated with amino acids (0.1 mM) in MeCN/H_2O (1:3) at 37 °C, and the resulting solutions were monitored via LC-MS over 15 min. (LC-MS condition: C_{18} column, 5 µm, 4.5*150 mm. Elution is water (1% TFA) /actonitrile = 20: 80, flow rate = 0.5 mL min^{-1})

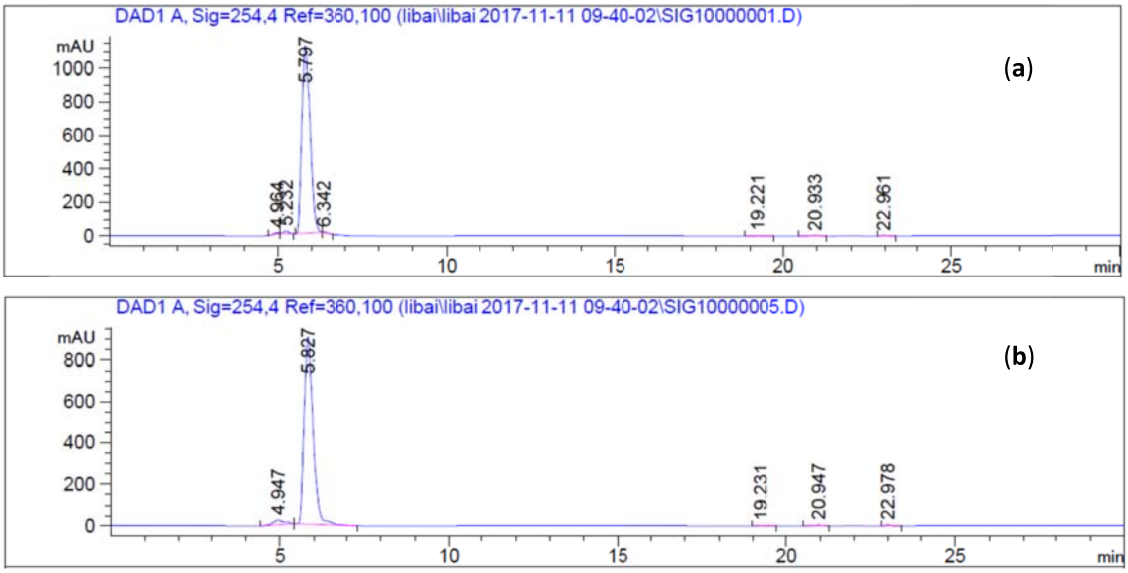

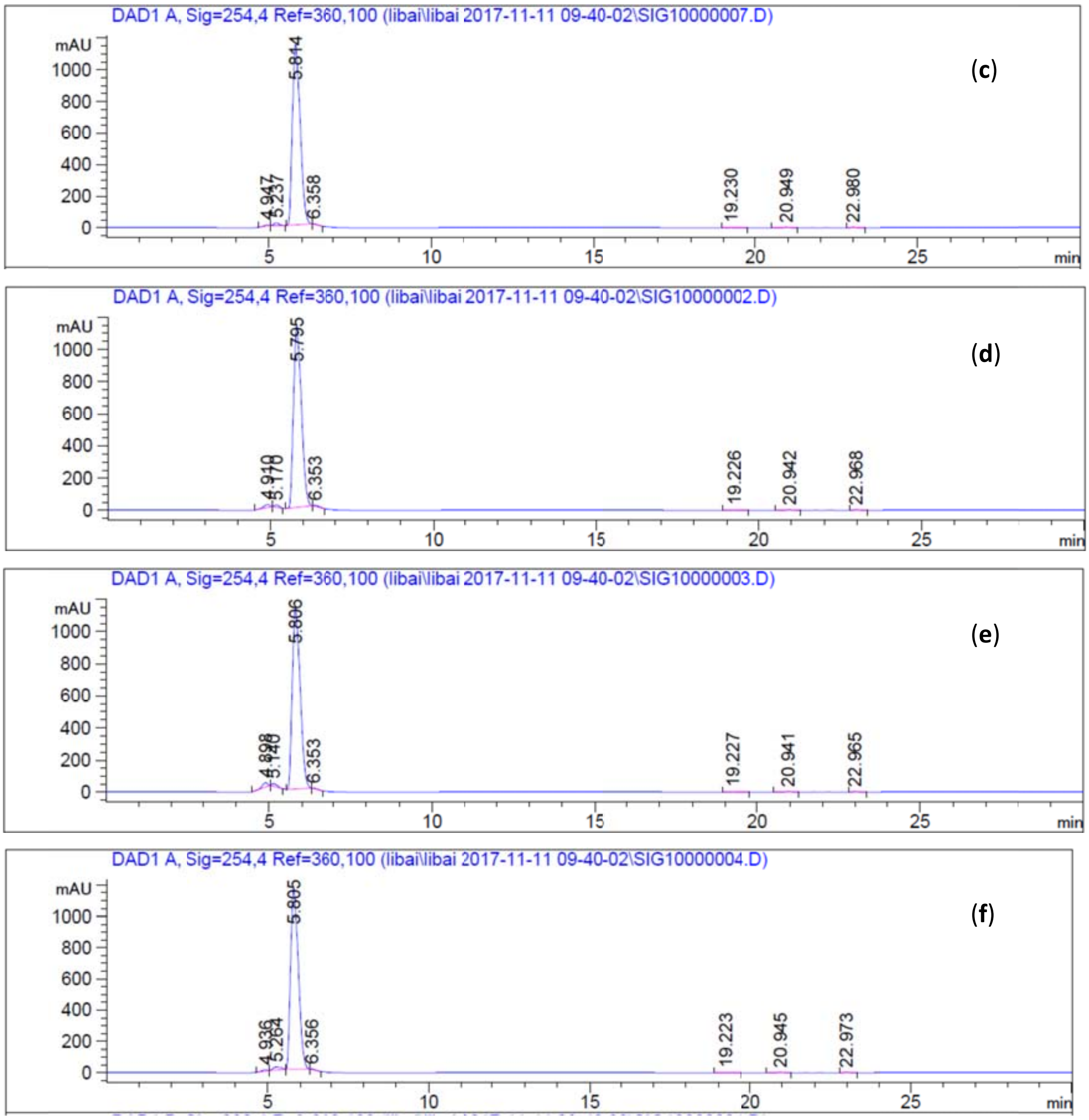

***Supplementary Figure S11*. The stability of probe 14**. Pure probe **14** (a) is treated with air (24 h, b), air (7 days, c), O_2 (24 h, d), H_2O_2 (100 nM, 24 h, e) and acid (pH~4.0, 24 h, f) at 37 ^{o}C, the retention is analyzed by LC-MS. (LC-MS condition: C_{18} column, 5 μm, 4.5*150 mm. Linear gradient elution, water (1% TFA) /actonitrile = 5: 95 to 95: 5 in 15 min, then the ratio is back to 5: 95 in 5 min, keep the ratio for 5 min.)

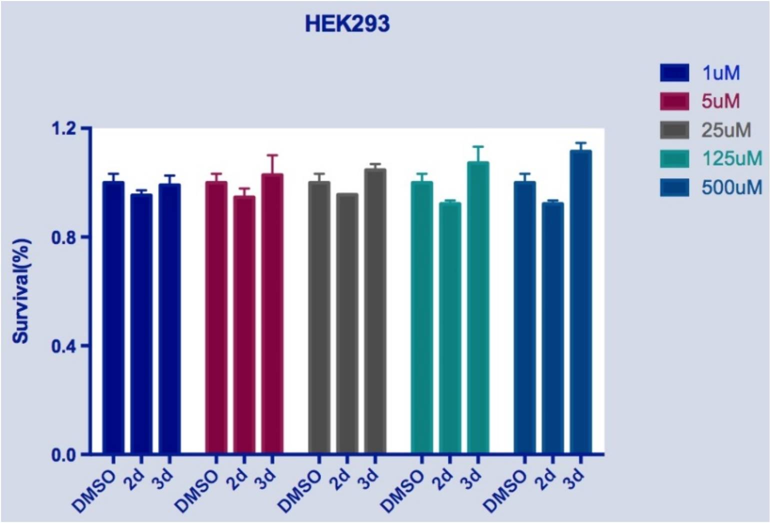

***Supplementary Figure S12*. The cytotoxicity of nitroso 8 and probe 14**. The *in vivo* cytotoxicity of **8** and **14** was tested by using CCK8 cell proliferation assay on 293 cell line. Cells were seeded in flat bottom 96-well plates (10000 per well) with 100.0 µL medium per well. **8** and **14** were added to each well to get desired final concentration (1.0 µM, 5.0 µM, 25.0 µM, 125.0 µM, 500.0 µM). It showed that there is no significant cytotoxicity of **8** and **14**.

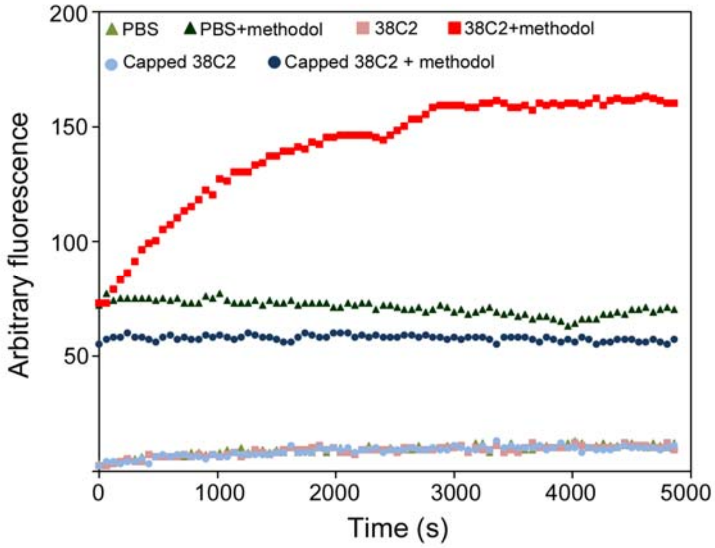

***Supplementary Figure S13*. Competitive inhibition experiment using compound 23**. 38C2 antibody (0.5 µM) was pre-treated with 5 µM compound **23** at room temperature for 2 h, followed by incubation with 5 µM methodol[8] at 37 °C for 3 h. All reactions were performed in PBS. The progress of the reaction was monitored by the change of fluorescence using TECAN 200 Pro plate reader (TECAN Systems). Fluorescence kinetics was recorded over a period of 3 h using an excitation wavelength of 330 nm and an emission wavelength of 452 nm.

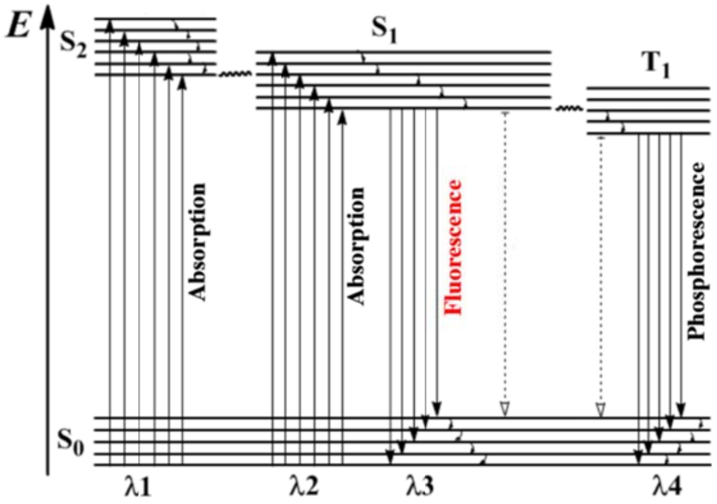

Supplementary Figure S14. The schematic diagram of absorption and emission spectrums for **14**.

For the matching problem between the calculated and measured absorption/emission wavelengths, we should point out: (1) Every compound exists the *ground state* (S_0) and *excited state* (such as *singlet excited state*, S_n, n=1, 2, 3...; and *triplet excited state*, T_n, n=1, 2, 3...), meanwhile, every state (including ground state and excited state) also exists several *vibration levels* (see Figure Sup3 as below); (2) When a compound has absorbed light radiation, the electron would jump from the *lowest vibration level* of the ground state (S_0) to the certain *vibration level* of the certain singlet excited state (S_n, n=1,2,3...), such as may jump to the lowest vibration level of S_2, or jump to the highest vibration level of S_1, or jump to the middle vibration level of S2, and so on; (3) Then, the jumped electron would relax to the lowest vibration level of S_1 via the vibration relaxation and internal energy conversion; (4) Final, similar to the absorption, the electron located in the lowest vibration level of S_1 would jump back to the certain vibration level of the ground state via the fluorescence emission, such as may jump back to the highest/middle/lowest vibration level of the ground state. Therefore, from the above four points, we can see that the measured absorption/emission wavelengths could accurate to the difference between the certain vibration levels, however, in the current computational level, it could only accurate to the difference between the certain states (S_0 and S_n, n=1,2,3...), could not accurate to the vibration level. So, the calculated absorption/emission wavelengths usually come out large deviations with the measured absorption/emission wavelengths due to the precision of the calculation. Based on these reasons, the value of stokes shift was often adopted to evaluate the fluorescence calculation, since the stoke shift is the difference value between the emission wavelength and absorption wavelength, and the difference value could counteract the error effect of the absorption/emission wavelengths. In our manuscript, the calculated λ_{abs} and λ_{em} of compound **14** were 418.98 nm and 551.52 nm, and the measured λ_{abs} and λ_{em} of compound **14** were 375 nm and 510 nm, respectively. The calculated wavelengths and the measured wavelengths indeed existed large deviations due to the precision of the calculation, but the calculated stokes shift (551.52 nm - 418.98 nm = 132.54 nm) and the

measured wavelengths (510 nm - 375 nm = 135 nm) were yet closed. Therefore, we considered that our fluorescence calculations were reasonable.

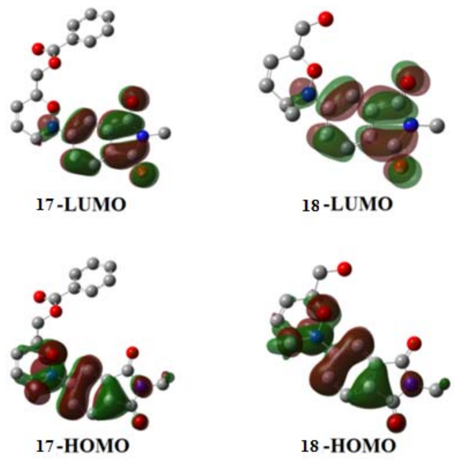

Supplementary Figure S15. HOMO-LUMO diagrams of compounds **17** *(left)* and **18** *(right)* in the ethanol.

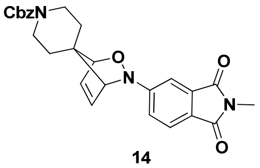

14

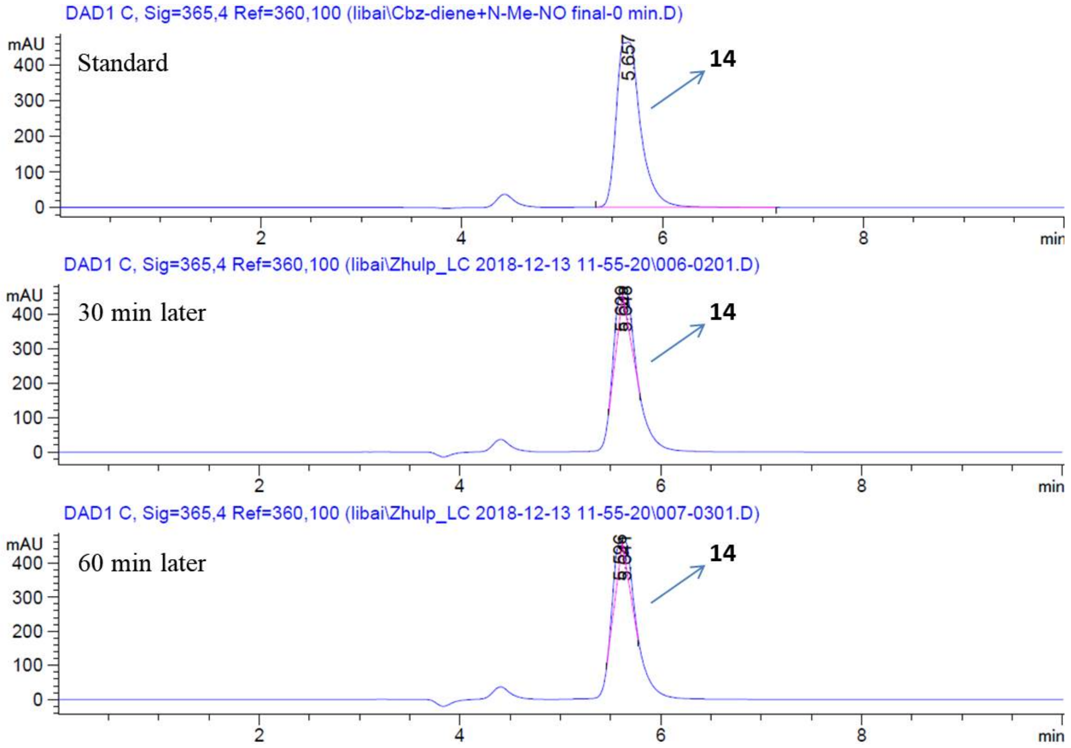

Compound **14** was dissolved in CH_3CN (10 μM), and the retention of **14** was analyzed by LC-MS. It showed that **14** is stable after 254 nm UV light irradiation for 30 and 60 min. (LC-MS condition: C_{18} column, 5 μm, 4.5*150 mm. Elution is water (1% TFA) /actonitrile = 20: 80, flow rate = 0.5 mL min^{-1}.)

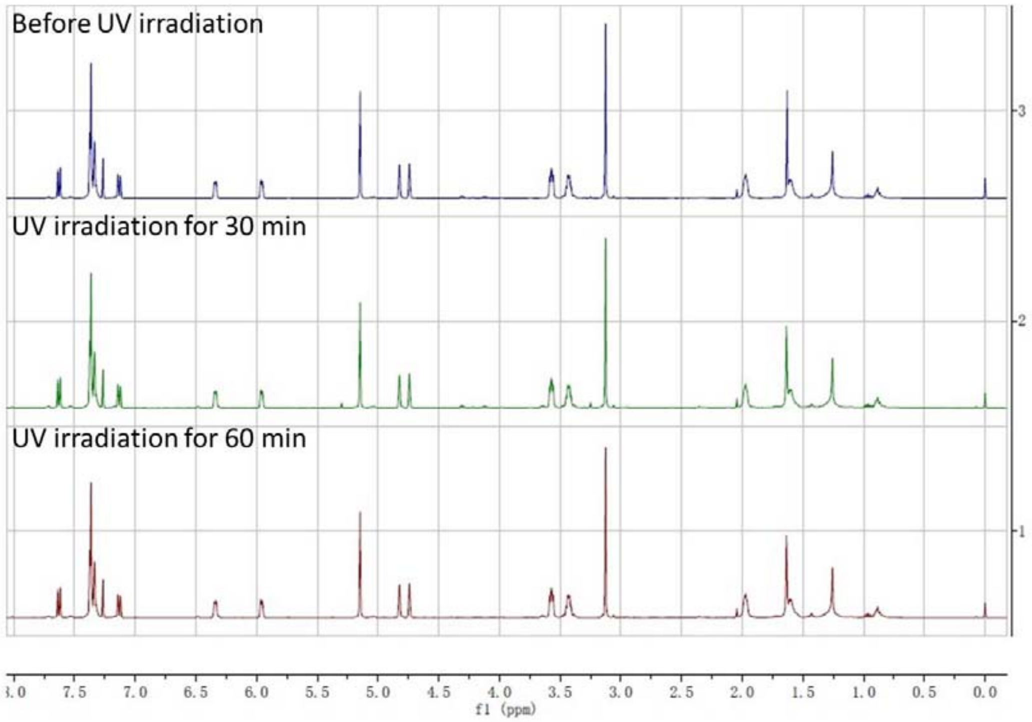

The stability of **14** was detected by 400 MHz ^{1}H NMR spectroscopy, the results proved that the structure of **14** had no change after UV light irradiation obviously.

Supplementary Figure S16. Stability detection of product **14** by ^{1}H NMR and LC-MS after 254 nm UV light irradiation.

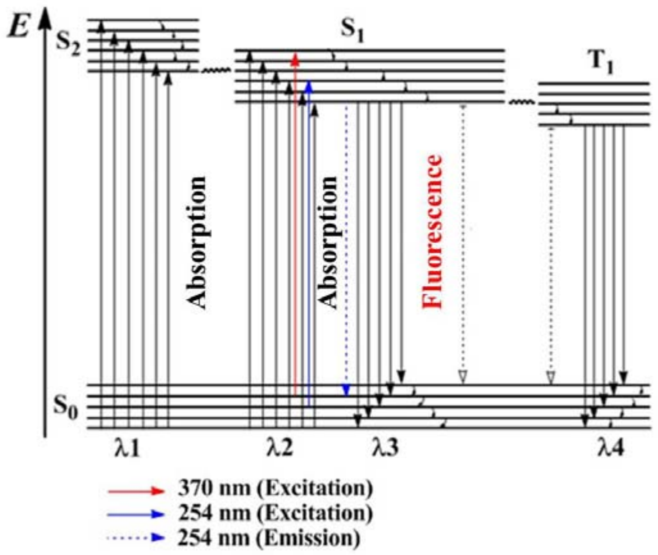

Explanation of mechanism: The results of ^{1}H NMR and LC-MS of product **14** showed there were no structure changes after 254 nm UV light irradiation. It means the fluorescence enhanced by UV radiation was not induced by structure changes. In *Supplementary Figure S14*, the DFT calculation results showed the probe **14** has a absorption peak at 264.8 nm, it lead to the fluorescence emission with a low quantum yield (Φ_f = 0.29). Combination of stability experimental results of **14** after 254 nm

UV light irradiation and DFT calculation results, we consider that the fluorescence can be enhanced by UV radiation maybe invited by the ground state change: (1) There are several energy levels in the ground state and excited state, respectively. The electrons excited by 254 nm and 370 nm are in different ground state energy level, and those excited electrons are also in different excited state energy levels. As shown in Figure (the red solid arrow and blue solid arrow). (2) When electrons excited by 254 nm return to the ground state, they may return to the ground state energy level corresponding to 370 nm (as shown in blue dotted arrow). So after 254 nm UV light irradiation, fluorescence can be enhanced at 370 nm. It means after 254 nm UV light irradiation, the fluorescence is much stronger than which is directly excited at 370 nm.

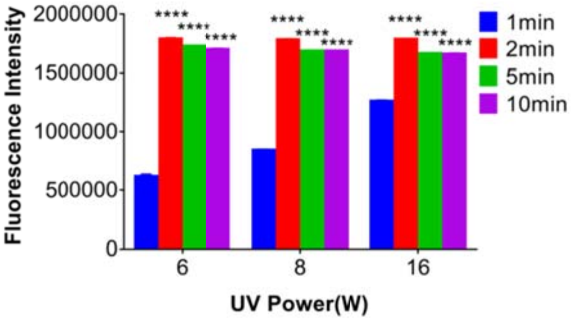

Supplementary Figure S17. Different power of UV lamp was used to irradiate compound **14** at 1 min, 2 min, 5 min and 10 min, respectively. **** showed after UV irradiation the fluorescent quantitation is significant difference at different time ($p < 0.0001$).The results showed that fluorescence intensity reached the max value for 2 min irradiation. But for 10 min irradiation, the fluorescence decreased slightly. This phenomenon maybe caused by photo-induced luminescence quenching.

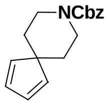

1

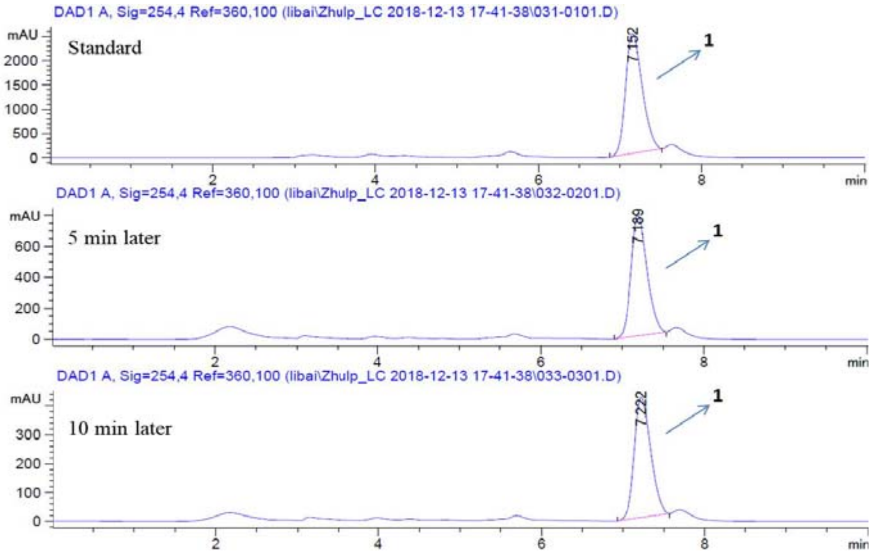

The stability of **1** (30 μM) in lysate of A431 cell (A431 cells, epidermoid carcinoma cell line, was performed to assess the stability of compound. 4×107 cells were digested with trypsin, harvested and exposed to 200 μl of RIPA lysis buffer (50 mM Tris-HCl (pH = 8.0), 150 mM NaCl, 0.02% NaN3, 0.1% SDS, 1% NP-40, 0.5% deoxysodium cholate, 1 mM EDTA, 1 μM RIPA). Then the upper liquid after centrifugation was obtained for further use) was analyzed by LC-MS, it showed that after 10 min later, compound 1 was consumed partly. (LC-MS condition: C_{18} column, 5 μm, 4.5*150 mm. Elution is water (1% TFA) /actonitrile = 20: 80, flow rate = 0.5 mL min^{-1}.)

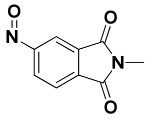

8

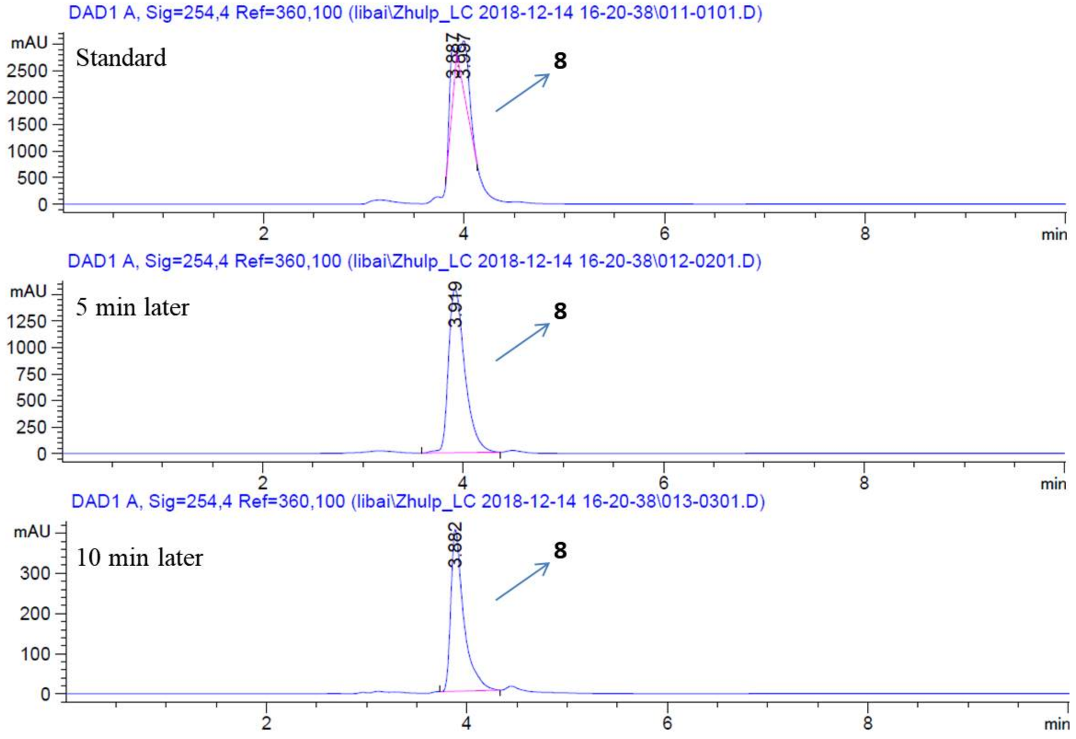

The stability of **8** (20 μM) in lysate of A431 cell was analyzed by LC-MS, it showed that after 10 min later, compound **8** was consumed partly. (LC-MS condition: C_{18} column, 5 μm, 4.5*150 mm. Elution is water (1% TFA) /actonitrile = 20: 80, flow rate = 0.5 mL min^{-1}.)

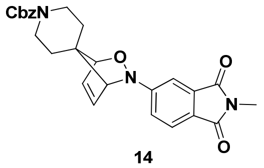

14

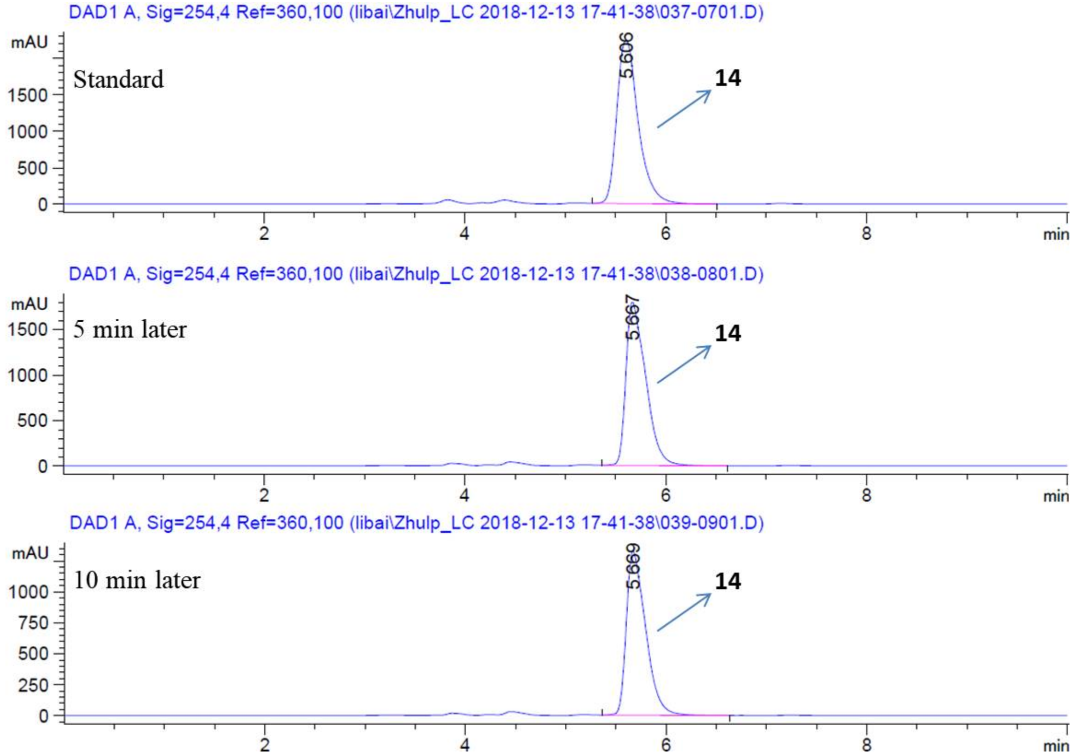

The stability of **14** (20 μM) in lysate of A431 cell was analyzed by LC-MS, it showed that after 10 min later, compound **14** had no change obviously. (LC-MS condition: C_{18} column, 5 μm, 4.5*150 mm. Elution is water (1% TFA) /actonitrile = 20: 80, flow rate = 0.5 mL min^{-1}.)

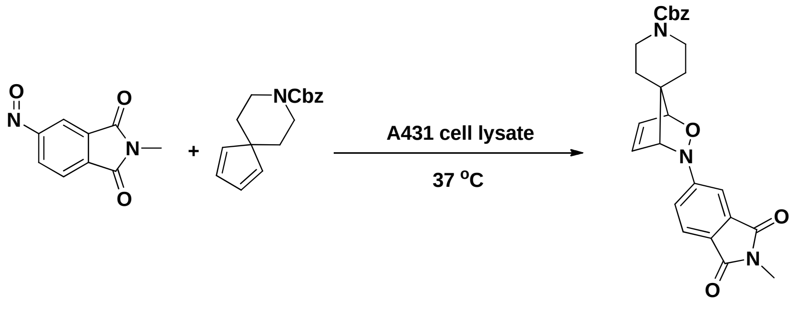

8 **1** **14**

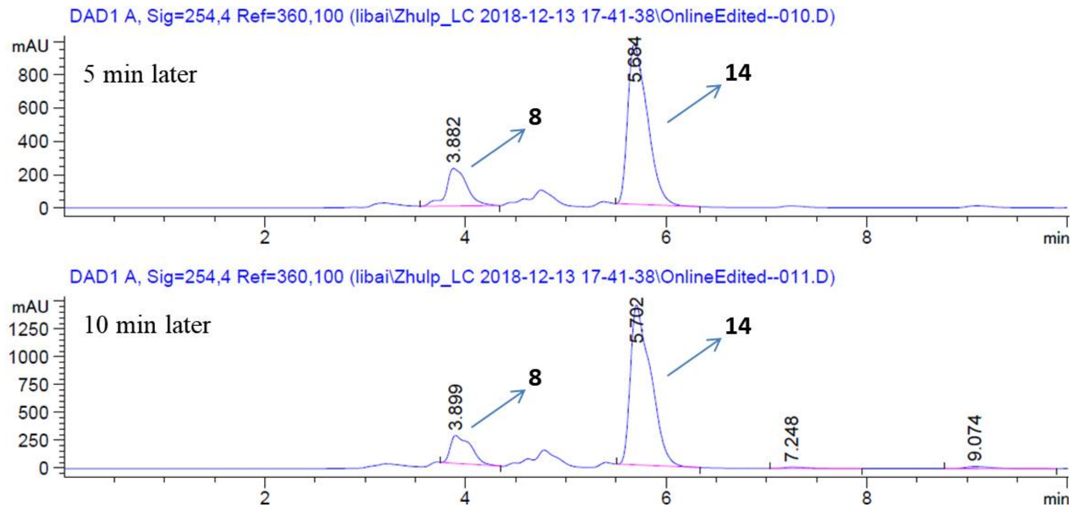

The competitive reactivity of compound **8** (30 μM) with compound **1**(20 μM) and A431 cell lysate was analyzed by LC-MS. The retention time (7.248) showed compound **1** is almost used up after 10 min. It means the reaction speed of **1** was better than lysate. (LC-MS condition: C_{18} column, 5 μm, 4.5*150 mm. Elution is water (1% TFA) /actonitrile = 20: 80, flow rate = 0.5 mL min^{-1}.)

Supplementary Figure S18. The stability experiments in lysate of A431 cell.

3. Supplementary Methods for Synthesis and Measurements

3.1 General procedure for chemical synthesis

All solvents were dried according to established procedures. Reactions were monitored by thin layer chromatography (TLC) and column chromatography purifications were carried out using silica gel GF254. Proton nuclear magnetic resonance(^{1}H NMR) spectra were recorded on Bruker 400 MHz spectrometer in $CDCl_3$ or $(CD_3)_2SO$, and carbon nuclear magnetic resonance(^{13}C NMR) spectra were recorded on 400 MHz spectrometer in $CDCl_3$ or $(CD_3)_2SO$ using tetramethylsilane (TMS) as internal standard unless otherwise noted. HR-MS was measured with an APEX II 47e mass spectrometer. Fluorescence measurements were performed using a Perkin Elmer LD-45 spectrophotometer equipped with a single cuvette reader. Ultraviolet absorption data was collected on a Thermo scientific NanoDrop 2000c UV-Vis Spectrophotometer.

3.2 Synthesis of nitroso and diene compounds

Diene compounds **1** and **2** were synthesized according to Lin *et al* [1,2] and Khan *et al* [3], respectively. (2*E*,4*E*)-hexa-2,4-dien-1-ol (**3**) and cyclohexa-1,3-diene (**4**) were commercially available from Acros. The aryl nitroso compounds (**5**, **6**, **7** and **9**) were synthesized according to Priewisch*et al* [4] and Molander*et al* [5].The nitroso compound **8** was synthesized as described below.

*3.3 Synthesis and characterization of 2-methyl-5-nitrosoisoindoline-1,3-dione (**8**)*

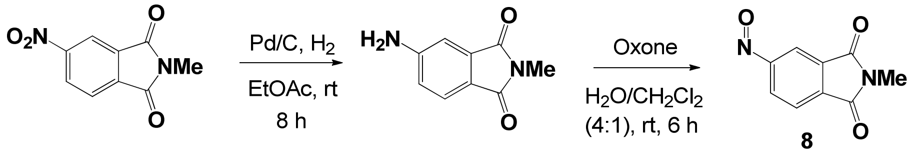

A few drops of concentrated acetic acid was added to a solution of 2-methyl-5-nitroisoindoline-1,3-dione (2.1 g, 10.0 mmol) in ethyl acetate (100.0 mL), and the reaction mixture was subjected to hydrogenation using palladium carbon (10%, ~ 300 mg) at room temperature with atmospheric pressure for 8 h. Progression of the reaction was monitored by TLC. Upon completion, the reaction was filtrated to remove palladium carbon and then concentrated by rotor evaporation to give 5-amino-2-methylisoindoline-1,3-dione (1.70 g) with a yield of 97%. The crude 5-amino-2-methylisoindoline-1,3-dione was dissolved in H_2O/CH_2Cl_2 (4:1 v/v) and oxone (21.5 g, 42.4 mmol) was added. The solution was stirred at room temperature until TLC indicated complete consumption of the starting materials. The aqueous layer was extracted with CH_2Cl_2 (3 x 30 mL) and washed successively with 1.0% HCl, 1.0% $NaHCO_3$ and brine.The combined organics were dried ($MgSO_4$), filtered, and concentrated. The crude product was purified over a pad of silica gel (4:1 hexanes/ethyl acetate), yielding **8** (1.2 g, 62%) as a pale yellow solid.

*2-methyl-5-nitrosoisoindoline-1,3-dione (**8**):*

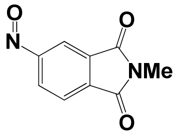

^{1}**H NMR** (400 MHz, $CDCl_3$) δ 8.54-8.56 (dd, J = 1.6 Hz, 8 Hz, 1 H), 8.19-8.22 (d, J = 7.6 Hz, 1 H), 8.03 (t, J = 4 Hz, 1H), 3.27 (m, 3H); ^{13}**C NMR** (100 MHz, $CDCl_3$) δ 166.9, 166.6, 164.5, 135.9, 133.7, 129.3, 125.0, 112.3, 24.6. **HRMS-ESI (*m/z*)**: calculated for $C_9H_6N_2O_3$+H^+: 191.0451; found: 191.0453.

3.4 Characterization of cycloaddition products

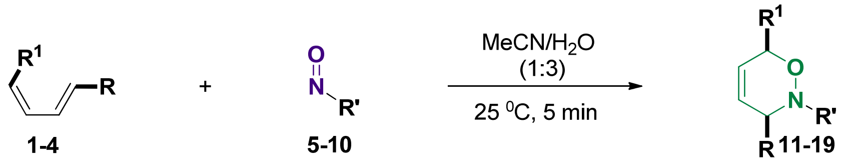

The nitroso compounds (0.22mmol) and dienes (0.20 mmol) were dissolved in MeCN/H_2O (1:3 v/v, 2.0 mL), and the reaction mixture was stirred at 25 ^{0}C for 5 min. The reaction was monitored by LCMS. Then, the resulting mixture was concentrated under reduced pressure and the residue was purified through column chromatography

on silica gel (1:1 to 4:1 hexanes/ethyl acetate) to give the corresponding product.

*Benzyl3-(4-(methoxycarbonyl)phenyl)-2-oxa-3-azaspiro[bicyclo[2.2.1]hept[5]ene-7,4'-piperidine]-1'-carboxylate (**11**):*

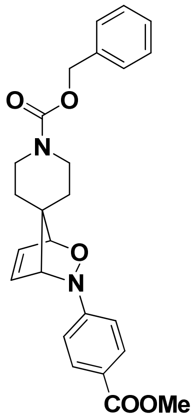

^{1}H NMR (400 MHz, $CDCl_3$) δ 7.86-7.89 (dd, *J* = 4 Hz, 8 Hz, 2 H), 7.35 (m, 5H), 6.92 (d, *J* = 8 Hz, 2H), 6.31 (m, 1H), 5.93 (m, 1H), 5.18 (s, 2H), 4.78 (s, 1H), 4.70 (s, 1H), 3.86 (s, 3H), 3.56-3.59 (m, 2H), 3.41-3.52 (m, 2H), 1.99 (m, 2H), 1.59-1.61 (m, 2H); **^{13}C NMR** (100 MHz, $CDCl_3$) δ 166.92, 154.87, 136.77, 132.73, 131.31, 130.42, 128.52, 128.04, 127.91, 122.89, 115.76, 85.51, 71.61, 67.13, 60.79, 51.80, 42.17, 41.62. **HRMS-ESI (*m*/*z*)**: calculated for $C_{25}H_{26}N_2O_5+H^+$: 435.1914; found: 435.1914.

*Benzyl 3-(4-acetylphenyl)-2-oxa-3-azaspiro[bicyclo[2.2.1]hept[5]ene-7,4'-piperidine]-1'-carboxylate (**12**):*

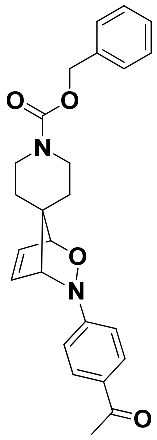

^{1}H NMR (400 MHz, $CDCl_3$) δ 7.88-7.90 (d, *J*= 8 Hz, 2H), 7.40-7.45 (m, 5H), 7.00-7.02 (d, *J* = 8 Hz, 2H), 6.39-6.41 (d, *J* = 8 Hz, 1H), 6.02-6.04 (d, *J* = 8Hz, 1H), 5.23 (s, 2H), 4.86 (s, 1H), 4.78 (s, 1H), 3.47-3.66 (m, 4H), 2.60 (s, 3H), 1.70 (m, 2H), 1.68 (m, 2H); **^{13}C NMR** (100 MHz, $CDCl_3$) δ 197.21, 155.42, 137.18, 133.16, 131.74, 131.01, 129.87, 128.92, 128.45, 128.33, 128.19, 116.14, 85.97, 71.99, 67.55, 61.23, 42.57, 42.03, 26.65. **HRMS-ESI (*m*/*z*)**: calculated for $C_{25}H_{26}N_2O_4+H^+$: 419.1965; found: 419.1963.

*Benzyl 3-(4-nitrophenyl)-2-oxa-3-azaspiro[bicyclo[2.2.1]hept[5]ene-7,4'-piperidine]-1'-carboxylate (**13**):*

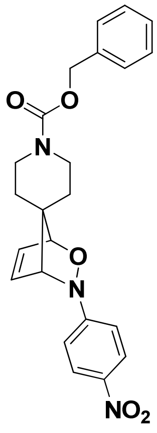

^{1}H NMR (400 MHz, $CDCl_3$) δ 8.14-8.17 (dd, *J* = 4Hz, 8Hz, 2H), 7.40-7.45 (m, 5H), 7.02-7.04 (dd, *J* = 4 Hz, 8 Hz, 2H), 6.41-6.43 (dd, *J* = 4 Hz, 8Hz, 1H), 6.07-6.09 (dd, *J* =4 Hz, 8 Hz, 1H), 5.23 (s, 2H), 4.91 (s, 1H), 4.81 (s, 1H), 3.45-3.70 (m, 4H), 2.04 (m, 2H), 1.69 (m, 2H);

^{13}C NMR (100 MHz, $CDCl_3$) δ 156.69, 141.99, 137.13, 133.08, 132.06, 128.93, 128.49, 125.28, 116.07, 86.32, 72.13, 67.59, 61.52, 42.52, 41.97. **HRMS-ESI (*m*/*z*)**: calculated for $C_{23}H_{23}N_3O_5$+H^+: 422.171; found: 422.1711.

*Benzyl 3-(2-methyl-1,3-dioxoisoindolin-5-yl)-2-oxa-3-azaspiro[bicyclo[2.2.1]hept[5]ene-7,4'-piperidine]-1'-carboxylate (**14**):*

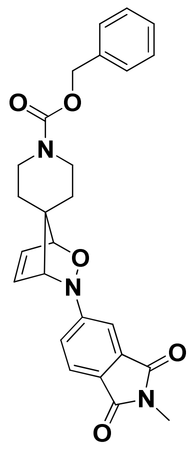

^{1}H NMR (400 MHz, $CDCl_3$) δ7.61-7.63 (d, *J* = 8 Hz, 1 H), 7.32-7.37 (m, 6H), 7.11-7.14 (dd, *J* = 4 Hz, 8 Hz, 1H), 6.32-6.35 (m, 1H), 5.94-5.97 (m, 1H), 5.140 (s, 2H), 4.81-4.83 (m, 1H), 4.73-4.75 (m, 1H), 3.56-3.58 (m, 2H), 3.40-3.44 (m, 2H), 3.12 (s, 3H), 1.86-1.98 (m, 2H), 1.58-1.60 (m, 2H); **^{13}C NMR** (100 MHz, $CDCl_3$) δ 168.55, 168.34, 156.37, 136.73, 133.67, 132.52, 131.71, 128.53, 128.07, 124.21, 123.96, 110.85, 99.98, 85.93, 72.03, 67.19, 61.14, 42.14, 41.58, 23.88. **HRMS-ESI (*m*/*z*)**: calculated for $C_{26}H_{25}N_3O_5$+H^+: 460.1867; found: 460.1869.

*Benzyl 3-(pyridin-2-yl)-2-oxa-3-azaspiro[bicyclo[2.2.1]hept[5]ene-7,4'-piperidine]-1'-carboxylate (**15**):*

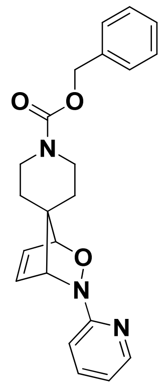

^{1}H NMR (400 MHz, $CDCl_3$) δ 8.19-8.20 (d, *J* = 4 Hz, 1H), 7.48-7.50 (t, *J* = 4 Hz, 1H), 7.34-7.38 (m, 5H), 6.78-6.81 (m, 2H), 6.23-6.25 (m, 1H), 6.04-6.06 (m, 1H), 5.15 (s, 3H), 4.75 (s, 1H), 3.64-3.70 (m, 1H), 3.47-3.56 (m, 2H), 3.31-3.38 (m, 1H), 2.03-2.10 (m, 1H), 1.80-1.93 (m, 1H), 1.56-1.63 (m, 2H); **^{13}C NMR** (100 MHz, $CDCl_3$) δ 163.41, 147.38, 137.51, 136.83, 134.39, 130.47, 128.50, 128.00, 127.89, 116.98, 111.91, 86.49, 70.10, 67.08, 60.26, 42.26, 41.66. **HRMS-ESI (*m*/*z*)**: calculated for $C_{22}H_{23}N_3O_3$+H^+: 378.1812; found: 378.1815.

*5-(2-oxa-3-azabicyclo[2.2.2]oct-5-en-3-yl)-2-methylisoindoline-1,3-dione (**16**):*

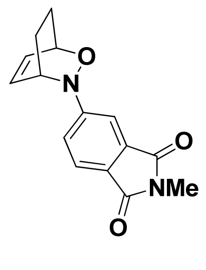

^{1}H NMR (400 MHz, $CDCl_3$) δ 7.64-7.66 (d, *J* = 8 Hz, 1H), 7.42-7.43 (d, *J* = 2 Hz, 1H), 7.21-7.23 (dd, *J* = 2.4 Hz, 8 Hz, 1H), 6.54-6.59 (m, 2H), 6.19-6.23 (m, 1H), 4.78-4.81 (dd, *J* = 4 Hz, 6 Hz, 1H), 4.62-4.64 (dd, *J* = 4 Hz, 5.6 Hz, 1H), 3.13 (s, 3H), 2.22-2.33 (m,

2H), 1.59-1.66 (m, 1H), 1.38-1.46 (m, 1H); **^{13}C NMR** (100 MHz, $CDCl_3$) δ 168.75, 168.43, 157.91, 133.68, 131.77, 129.99, 124.26, 123.90, 120.69, 111.18, 70.09, 55.93, 23.87, 23.81, 20.97. **HRMS-ESI (*m/z*)**: calculated for $C_{15}H_{14}N_2O_3+H^+$: 271.1077; found: 271.1084.

*(2-(2-methyl-1,3-dioxoisoindolin-5-yl)-3,6-dihydro-2H-1,2-oxazin-6-yl)methyl benzoate (**17**):*

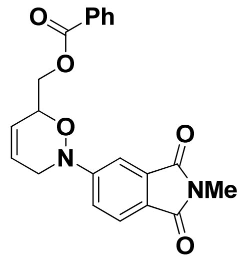

^{1}H NMR (400 MHz, $CDCl_3$) δ 8.16-8.19(m, 2H), 7.54-7.75(m, 5H), 7.29-7.36(m, 1H), 6.03-6.19(m, 2H), 4.93-4.97(m, 1H), 4.49-4.75(m, 2H), 3.96-4.12(m, 2H), 3.18 (s, 2.5H), 3.10 (s, 0.5H); **^{13}C NMR** (100 MHz, $CDCl_3$) δ 168.42, 166.37, 154.69, 134.11, 133.36, 129.76, 129.52, 128.62, 128.11, 125.09, 124.96, 124.27, 124.13, 118.14, 108.97, 76.89, 63.86, 49.75, 23.92. **HRMS-ESI (*m/z*)**: calculated for $C_{21}H_{18}N_2O_5+H^+$: 379.1288; found: 379.1292.

*5-(6-(hydroxymethyl)-3-methyl-3,6-dihydro-2H-1,2-oxazin-2-yl)-2-methylisoindoline-1,3-dione (**18**):*

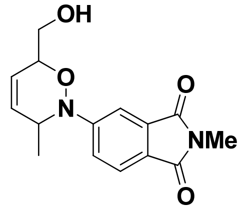

^{1}H NMR (400 MHz, $CDCl_3$) δ 7.66-7.71(m, 1H), 7.38-7.39(m, 1H), 7.16-7.22(m, 1H), 5.79-5.94(m, 2H), 4.74-4.79(m, 0.7H), 4.61-4.64(m, 0.3H), 4.31-4.39(m, 1H), 3.13 (s, 3H), 1.32 (d, J = 6.8 Hz, 1H), 1.19 (d, J =6.8 Hz, 2H); **^{13}C NMR** (100 MHz, $CDCl_3$) δ 168.74, 168.48, 152.05, 152.95, 134.48, 134.25, 131.99, 130.58, 124.87, 124.62, 124.44, 123.05, 122.64, 117.88, 116.97, 108.58, 107.85, 79.24, 71.69, 63.72, 62.71, 58.56, 53.88, 23.89, 23.88, 18.73, 14.79. **HRMS-ESI (*m/z*)**: calculated for $C_{15}H_{16}N_2O_4$ +H^+: 289.1183; found: 289.1188.

*Benzyl 3-(1,3-dioxo-2-phenylisoindolin-5-yl)-2-oxa-3-azaspiro[bicyclo[2.2.1]hept[5] ene-7,4'-piperidine]-1'-carboxylate (**19**):*

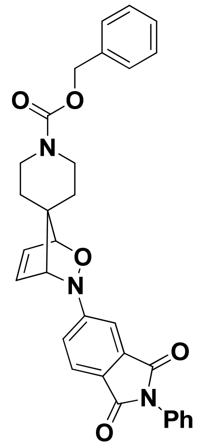

^{1}H NMR (500 MHz, $CDCl_3$) δ 7.75-7.73 (d, J = 10 Hz, 1H), 7.50-7.47 (m, 2H), 7.44-7.32 (m, 9H), 7.23-7.21 (dd, J = 1.6, 8 Hz, 1 H), 6.37-6.35 (m, 1H), 6.01-6.00 (m, 1H), 5.15 (s, 2H), 4.85 (s, 1H), 4.78 (s, 1H), 3.59-3.57 (m, 2H), 3.45-3.42 (m, 2H), 1.99 (s, 2H), 1.61

(s, 2H); ^{13}**C NMR** (125 MHz, $CDCl_3$) δ 167.35, 167.10, 156.83, 136.71, 133.23, 132.60, 131.89, 131.78, 129.07, 128.54, 128.09, 127.95, 126.55, 124.58, 123.61, 121.02, 111.13, 86.01, 72.05, 67.20, 61.21, 42.14, 41.59. **HRMS-ESI (*m/z*)**: calculated for $C_{15}H_{16}N_2O_4$ +Na^+: 544.1843; found: 544.1838.

3.5 Spectroscopic Measurements

All spectroscopic measurements of **14**, **17** and **18** were performed in ethanol using a cuvette with 1-cm path length at 25 ± 0.1°C. All solutions were degassed under argon for several minutes prior to measurements. For each experiment, the slit width was 2.0 nm for both excitation and emission. The absorbance spectra were measured within an absorbance range of 0.07 to 0.7 (l = 10 cm). Fluorescence quantum yield measurements were performed on a fluorometer and UV-Vis instrument. Relative quantum efficiencies were obtained by comparing the areas under corrected emission spectrum. The reported quantum yield was calculated as an average of 4 points according to the following equation:

$$\Phi_{sample} = \Phi_{standard}\,(A_{standard} / A_{sample})\,(F_{sample} / F_{standard})\,(n_{sample} / n_{standard})^2$$

where "Φ" is the quantum yield, "A" is the absorbance at the excitation frequency, "F" is the integrated area under the emission curve, and "n" is the refractive index of the solvent used. Fluorescein (Φ_f = 0.85) [8] in 0.1 M aqueous NaOH in ethanol are fluorescence standards.

3.6 Reaction kinetic measurements

A Nanodrop 2000c (Thermo Scientific) equipped with a cuvette reader and a stirrer was used in the kinetic measurements, and the increasment of the in-situ fluorescent HDA products peak absorption around 365 nm was measured over time. The dienophile (**8**) at 1.0 mM were reacted with an excess 10.0 mM dienes (**1**, **2** and **3**) with the measurements commencing immediately upon dienes (**1**, **2** and **3**) (Figure S4 and S5). Solution conditions were maintained at 4:1 DMF : PBS pH 7.4 buffer (Sigma Life Science), and all the measurements were done at 25 °C room temperature.

Product peak intensity at each time point was background-adjusted by subtracting an extrapolated straight line between the intensities preceding and following the peak. Product peak increasement is shown as points in Figure S5, with the connecting lines resulting from the Exponential Growth 1 fits made with Origin. Resulting observed pseudo-first-order k_{obs} values were converted to the reported k_2 second-order rate constants as k_2=k_{obs}/[diene]$_0$.

3.7 Preparation of dienes for labeling

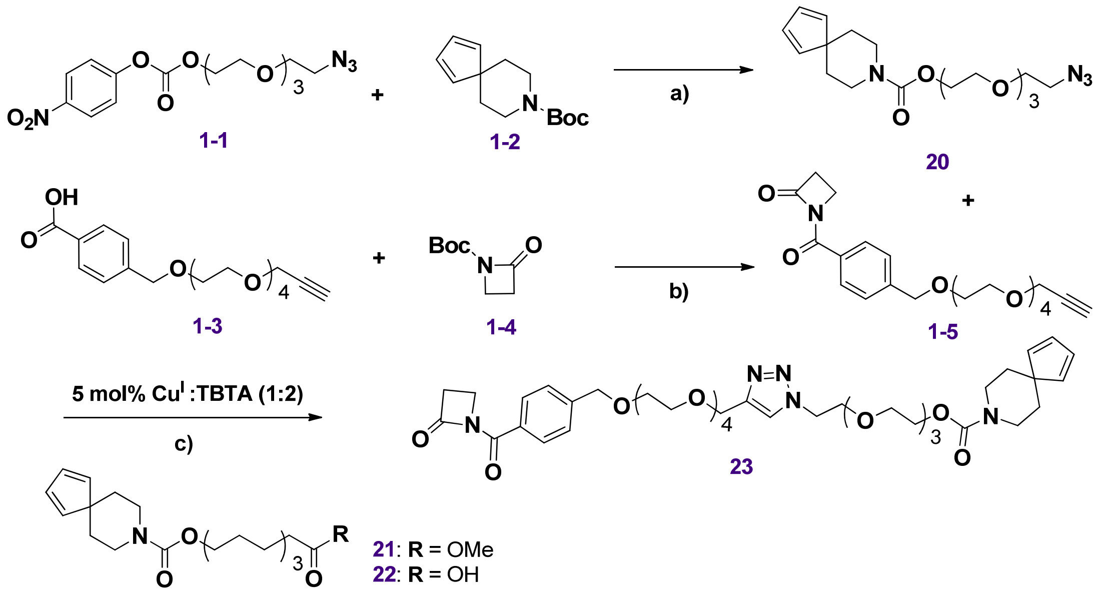

Reagents and conditions: a) TFA in CH_2Cl_2, ice bath, 0.5 h; DIEA, rt, 6 h, 75% for **20**; the same synthetic strategy for **21** and **22**. b) $SOCl_2$, 3 h; 2.5M *n*-BuLi in THF, -78 ^{0}C, 4.5 h, 55% over two steps. c) 5 mol% CuBr, 10 mol% TBTA, 5 mol% Ascorbic acid, THF, rt, 6 h, 93%.

*2-(2-(2-(2-azidoethoxy)ethoxy)ethoxy)ethyl (4-nitrophenyl) carbonate (**1-1**):*

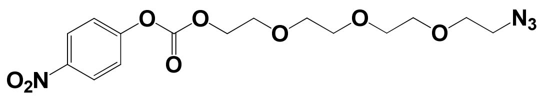

The 2-(2-(2-(2-azidoethoxy)ethoxy)ethoxy) ethyl (4-nitrophenyl) carbonate was synthesized according to reported literature procedures (31% yield).[6] **^{1}H NMR** (400 MHz, $CDCl_3$) δ 8.23-8.24 (d, *J* = 3.6 Hz, 2H), 7.35-7.36 (d, *J* = 3.6 Hz, 2H),

4.01-4.08(m, 2H), 3.77-3.78(m, 2H), 3.65-3.66(m, 10H), 3.54(m, 2H); ^{13}**C NMR** (100 MHz, $CDCl_3$) δ 155.51, 152.44, 145.34, 125.25, 121.78, 70.63, 70.00, 68.57, 68.30, 50.63. **HRMS-ESI (*m/z*)**: calculated for $C_{15}H_{20}N_4O_8+H^+$: 385.1354; found: 385.1358.

*tert-butyl 8-azaspiro[4.5]deca-1,3-diene-8-carboxylate (**1-2**)*[1,2]*:*

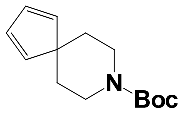

Sodium hydride (3.0 g, 62 mmol, 60% in the mineral oil) was added to a 250-mL two-necked round-bottomed flask, and then anhydrous dimethyl sulfoxide (30 mL) was injected by syringe. The solution was warmed to 75 ºC for 45 min under N2 atmosphere to give the grey suspension. The suspension was cooled to 0 ºC, and then anhydrous tetrahydrofuran (75 mL) was added. 10 min later, freshly distilled cyclopentadiene (3.8 mL, 45 mmol, in a 20 mL of tetrahydrofuran) was added dropwise under 0 ºC. A large amount of white precipitate formed and visgorous stirring was possibly required. After stirring for 15 min, *N*-Boc-*N*, *N*-bis(2-chloroethyl)amine (4.8 g, 20 mmol, in 20 mL of tetrahydrofuran) was added by syringe and reaction mixture keeps stirring at room temperature overnight. The solution was quenched with saturated aqueous ammonium chloride (40 mL) and extracted with diethyl ether (3*60 mL). The combined organic layers were washed with brine (100 mL) and dried with Na_2SO_4. The resulting liquid was filtered and concentrated under reduced pressure. The residue was chromatographed on silica (petroleum ether:EtOAc = 30:1) to yield 2.58 g (55%) of 1-2 as a yellow oil.

The *tert*-butyl 8-azaspiro[4.5]deca-1,3-diene-8-carboxylate was synthesized according to reported literature procedures (55% yield).[1,2] ^{1}**H NMR** (400 MHz, $CDCl_3$) δ 6.47-6.49 (dd, *J* = 2.4 Hz, 5.2 Hz, 2H), 6.29-6.31 (dd, *J* = 2.4 Hz, 5.2 Hz, 2H), 3.54-3.57(m, 4H), 1.51-1.54(m, 4H), 1.47(s, 9H); ^{13}**C NMR** (100 MHz, $CDCl_3$) δ 154.97, 129.47, 79.46, 79.44, 55.40, 39.45, 30.89, 28.55, 28.48. **HRMS-ESI (*m/z*)**: calculated for $C_{14}H_{21}NO_2$ $+H^+$: 236.1645; found: 236.1651.

2-(2-(2-(2-azidoethoxy)ethoxy)ethoxy)ethyl 8-azaspiro[4.5]deca-1,3-diene-8-carboxy

late (20):

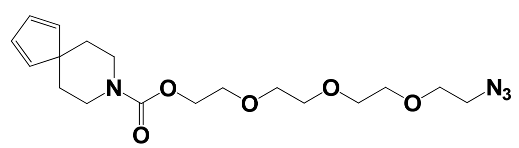

To a solution of **1-2** (0.47 g, 2.0 mmol) in CH_2Cl_2 (10 mL) was added TFA (0.8 mL) portionwise at ice bath, and the reaction was stirred at room temperature for 0.5 h. The reaction was monitored by TLC. Then, the resulting mixture was concentrated under reduced pressure and the 8-azaspiro[4.5]deca-1,3-diene trifluoroacetate was obtained. In the round bottom flask (20 mL CH_2Cl_2) of 8-azaspiro[4.5]deca-1,3-diene trifluoroacetate, *N*,*N*-Diisopropylethylamine (0.2 mL) was added at ice bath. Then, a solution of **1-1** (0.77 g, 2.0 mmol) in CH_2Cl_2 (10 mL) was added, and the reaction was stirred at ice bath to room temperature for 6 h. After reaction, the solvent was removed in vacuo, the residue was purified by column chromatography on silica gel to afford compound **20** as a colorless oil (0.57 g, 75%). **^{1}H NMR** (400 MHz, $CDCl_3$) δ 6.55-6.56 (dd, *J* = 2 Hz, 4.4 Hz, 2H), 6.38-6.39 (dd, *J* = 2 Hz, 4.4 Hz, 2H), 4.32-4.34(m, 2H), 3.82-3.84(m, 2H), 3.78-3.80(m, 2H), 3.68-3.75(m, 12H), 3.45-3.47(m, 2H), 1.61-1.63(m, 4H); **^{13}C NMR** (100 MHz, $CDCl_3$) δ 155.87, 142.81, 130.03, 71.12, 71.08, 70.96, 70.07, 64.92, 62.11, 55.67, 51.09, 43.77, 43.29. **HRMS-ESI (*m*/*z*)**: calculated for $C_{18}H_{28}N_4O_5+H^+$: 381.2132; found: 381.2134.

*Methyl 10-(((4-nitrophenoxy)carbonyl)oxy)decanoate (**1-1b**):*

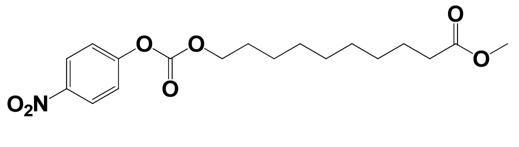

Compound **1-1b** was prepared from 4-nitrophenyl chloroformate and methyl 10-hydroxydecanoate in a manner similar to that described for compound **1-1** with a yield of 75% as a colorless solid. **^{1}H NMR** (400 MHz, $CDCl_3$): δ (ppm) 8.36 (d, *J* = 7.6 Hz, 2H), 7.47 (d, *J* = 7.6 Hz, 2H), 4.37 (t, *J* = 5.4 Hz, 2H), 3.75 (s, 3H), 2.39 (t, *J* = 6.0 Hz, 2H), 1.87-1.81 (m, 2H), 1.71 (t, *J* = 5.8 Hz, 2H), 1.50-1.40 (m, 10H); **^{13}C NMR** (100 MHz, $CDCl_3$): δ (ppm) 174.66, 156.03, 152.95, 145.05, 125.68, 125.42, 122.65, 122.20, 70.04, 51.84, 49.02, 34.46, 29.62, 29.52, 29.47, 28.88, 26.00, 25.30.

HRMS-ESI (*m/z*): calculated for $C_{18}H_{25}NO_7$+Na^+: 390.1523; found: 390.1529.

*10-methoxy-10-oxodecyl 8-azaspiro[4.5]deca-1,3-diene-8-carboxylate (**21**):*

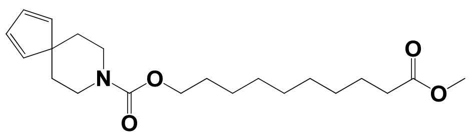

Compound **21** was prepared from **1-2** and **1-1b** in a manner similar to that described for compound **20** with a yield of 61% as a colorless solid. **^{1}H NMR** (400 MHz, $CDCl_3$): δ (ppm) 6.57 (d, *J* = 4.0 Hz, 2H), 6.39 (d, *J* = 8.0 Hz, 2H), 4.16 (t, *J* = 8.0 Hz, 2H), 3.74 (s, 3H), 3.68 (t, *J* = 4.0 Hz, 4H), 2.38 (t, *J* = 6.0 Hz, 2H), 1.74-1.68 (m, 4H), 1.62 (t, *J* = 4.0 Hz, 4H), 1.53-1.33 (m, 10H); **^{13}C NMR** (100 MHz, $CDCl_3$): δ (ppm) 174.72, 156.20, 142.87, 129.99, 65.93, 55.73, 51.85, 43.68, 34.50, 29.72, 29.62, 29.57, 29.50, 29.44, 26.35, 25.33. **HRMS-ESI (*m/z*)**: calculated for $C_{21}H_{33}NO_4$+H^+: 364.2482; found: 364.2486.

*10-((8-azaspiro[4.5]deca-1,3-diene-8-carbonyl)oxy)decanoic acid (**22**):*

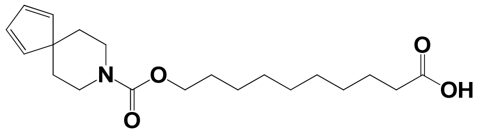

Compound **22** was prepared from **1-2** and 10-hydroxydecanoic acid in a manner similar to that described for compound **20** with a yield of 55% as a colorless solid. **^{1}H NMR** (400 MHz, $(CD_3)_2SO$): δ (ppm) 6.67 (dd, *J* = 4.0, 2.0 Hz, 2H), 6.37 (dd, *J* = 4.4, 2.4 Hz, 2H), 4.06 (t, *J* = 5.2 Hz, 2H), 3.60 (t, *J* = 4.4 Hz, 4H), 2.25 (t, *J* = 5.8 Hz, 2H), 1.63 (t, *J* = 5.6 Hz, 2H), 1.56-1.48 (m, 6H), 1.37-1.32 (m, 10H); **^{13}C NMR** (100 MHz, $(CD_3)_2SO$): δ (ppm) 175.36, 155.67, 143.81, 130.14, 65.57, 56.11, 43.57, 34.52, 31.09, 29.65, 29.50, 29.45, 29.37, 26.26, 25.35. **HRMS-ESI (*m/z*)**: calculated for $C_{20}H_{31}NO_4$+H^+: 350.2326; found: 350.2328.

*1-(4-(2,5,8,11,14-pentaoxaheptadec-16-yn-1-yl)benzoyl)azetidin-2-one (**1-5**):*

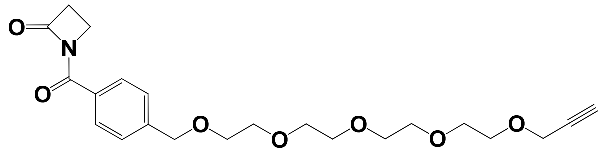

Compound **1-3**[7] (0.73 g, 2.0 mmol) was dissolved in $SOCl_2$ (8 mL), and the reaction was stirred at 0 °C to room temperature for 3 h. After reaction, the reaction mixture was concentrated under reduced pressure, and the residue was diluted with dichloromethane. The solution was washed with saturated sodium bicarbonate solution and brine, dried over anhydrous sodium sulfate. The solvent was removed in vacuo to afford 4-(2,5,8,11,14-pentaoxaheptadec-16-yn-1-yl)benzoyl chloride as a colorless oil (0.75 g, 98%). To a solution of 2-azetidinone from **1-4** (0.16 g, 2.3 mmol) in THF (8 mL) was added 2.5M *n*-BuLi/hexane solution (0.91 mL, 2.3 mmol) dropwise at -78 °C, and the reaction was stirred at -78 °C for 1 h. Then, a solution of benzoyl chloride (0.75 g) in THF (5.0 mL) was added to the reaction at -78 °C, and the reaction was stirred for 3.5 h. After reaction, the reaction was added by adding 10% citric acid solution, and the mixture was extracted with ethyl acetate (5*30 mL), washed with saturated sodium bicarbonate solution and brine, dried over anhydrous sodium sulfate. The solvent was removed in vacuo, the residue was purified by column chromatography on silica gel to afford compound **1-5** as a colorless oil (0.46 g, 55%). **^{1}H NMR** (400 MHz, $CDCl_3$) δ 7.96-7.98 (d, *J* = 8 Hz, 2H), 7.44-7.46 (d, *J* = 8 Hz, 2H), 4.62-4.66(t, *J* = 8 Hz, 2H), 4.18-4.21 (dd, *J* = 4 Hz, 8 Hz, 2H), 3.67-3.69(m, 18H), 3.10-3.13 (d, *J* = 8 Hz, 2H), 2.41-2.44 (t, *J* = 4 Hz, 1H); **^{13}C NMR** (100 MHz, $CDCl_3$) δ 165.99, 163.93, 144.09, 130.97, 129.91, 126.92, 79.68, 74.50, 72.56, 70.69, 70.62, 70.40, 69.90, 69.12, 58.39, 36.77, 35.02. **HRMS-ESI (*m/z*)**: calculated for $C_{22}H_{29}NO_7+H^+$: 420.2017; found: 420.2018.

*2-(2-(2-(2-(4-(15-(4-(2-oxoazetidine-1-carbonyl)phenyl)-2,5,8,11,14-pentaoxapentadecyl)-1H-1,2,3-triazol-1-yl)ethoxy)ethoxy)ethoxy)ethyl 8-azaspiro[4.5]deca-1,3-diene-8-carboxylate (**23**):*

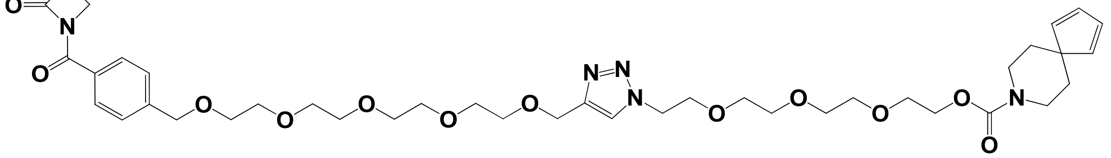

To a solution of compound **1-5** (209 mg, 0.5 mmol) and compound **20** (209 mg, 0.55 mmol) in THF (20 mL) was added 5.0 mol% CuBr (3.5 mg, 0.025 mmol), 5.0 mol%

Ascorbic acid (4.4 mg, 0.025 mmol) and 10.0 mol% Tris[(1-benzyl-1H-1,2,3-triazol-4-yl)methyl] amine (26.5 mg, 0.05 mmol). The reaction was monitored by LCMS, and was stirred at room temperature for 6 h. Then, the resulting mixture was concentrated under reduced pressure and the residue was purified through column chromatography on silica gel (1:2 to 1:1 hexanes/ethyl acetate) to give the product **23** (371.5 mg, 93% yield). **^{1}H NMR** (400 MHz, $CDCl_3$) δ 7.94-7.97 (d, J = 8.4 Hz, 2H), 7.73 (s, 1H), 7.43-7.45 (d, J = 8 Hz, 2H), 6.47-6.48 (dd, J = 2.4 Hz, 5.6 Hz, 2H), 6.30-6.32 (dd, J = 2.4 Hz, 5.6 Hz, 2H), 4.68 (s, 2H), 4.62 (s, 2H), 4.51-4.53 (dd, J = 4.8 Hz, 5.6 Hz, 2H), 4.24-4.26 (dd, J = 4.8 Hz, 6 Hz, 2H), 3.85-3.87 (dd, J = 4.4 Hz, 5.2 Hz, 2H), 3.77-3.79(m, 2H), 3.59-3.72(m, 30H), 3.09-3.13 (dd, J = 5.2 Hz, 5.6 Hz, 2H), 1.69(m, 4H); **^{13}C NMR** (100 MHz, $CDCl_3$) δ 166.39, 164.34, 155.83, 145.36, 144.49, 142.79, 130.31, 130.07, 127.32, 124.17, 72.96, 71.08, 71.06, 71.02, 70.98, 70.93, 70.91, 70.31, 70.08, 70.06, 69.88, 65.00, 64.84, 55.65, 50.61, 43.77, 37.19, 35.43, 30.09. **HRMS-ESI (*m/z*)**: calculated for $C_{40}H_{57}N_5O_{12}+H^+$: 800.4076; found: 800.4076.

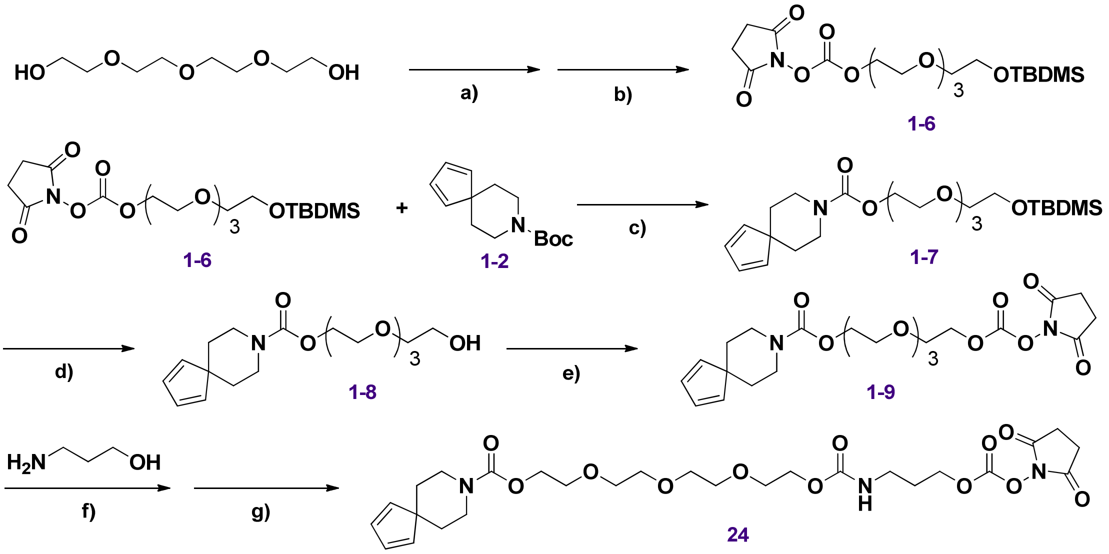

Reagents and conditions: a) TBDMSCl, imidazole, DMF, rt, overnight, 35%. b) *N*,*N*-disuccinimidyl carbonate (DSC), Et_3N, CH_3CN, rt, 12 h, 34%. c) TFA in CH_2Cl_2, ice bath, 0.5 h; DIEA, rt, 2 h, 48%. d) HF-pyridine, CH_2Cl_2, 30 min, 65%. e) DSC, Et_3N, CH_3CN, rt, 12 h, 70%. f) 3-Amino-1-propanol, DIEA, CH_2Cl_2, rt, 77%. g) DSC,

Et_3N, CH_3CN, rt, 12 h, 77%.

2,5-dioxopyrrolidin-1-yl

*(2,2,3,3-tetramethyl-4,7,10,13-tetraoxa-3-silapentadecan-15-yl) carbonate (**1-6**)*

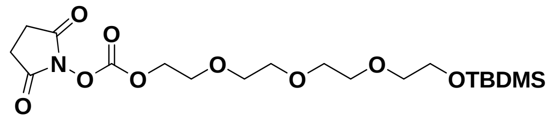

Compound **1-6** was synthesized in two steps according to reported literature procedures as below [9,10]: To a solution of tetraethylene glycol (TEG) (7.8 g, 40.0 mmol) in DMF (16 mL), TBDMSCl (2.0 g, 13.4 mmol) and imidazole (1.8 g, 26.6 mmol) were added and stirred overnight. The reaction was quenched with saturated $NaHCO_3$ and partitioned between ethyl acetate and water. The organic layer was washed with brine and dried with Na_2SO_4. The product was concentrated under reduced pressure and the residuals were purified through column chromatography on silica gel (2:1 to 1:1 petroleum/ethyl acetate) to give the mono-protected TEG as a colorless oil (1.45 g, 35%). To a solution of mono-protected TEG (1.23 g, 4 mmol) and *N*,*N*-disuccinimidyl carbonate (DSC) (2.31 g, 6 mmol) in CH_3CN (16 mL) was added Et_3N (1.68 mL, 12 mmol). The resulting solution was stirred overnight at room temperature and then monitored by TLC. Upon completion of the reaction, 100 mL CH_2Cl_2 was added and the mixture was washed twice with 50 mL of 5% aqueous $NaHCO_3$ and water. The organic layer was dried (Na_2SO_4) and filtered, and the solvent was removed under reduced pressure. The crude product was purified through column chromatography on silica gel (2:1 petroleum/ethyl acetate) to afford compound **1-6** as colorless oil (0.61 g, 34%). **^{1}H NMR** (400 MHz, $CDCl_3$) δ 4.47-4.45 (t, *J* = 4.8 Hz, 2H), 3.80-3.75 (m, 4H), 3.67-3.66 (d, *J* = 4 Hz, 8H), 3.57-3.54 (t, *J* = 5.6 Hz, 2H), 2.83 (s, 4H), 0.89 (s, 9H), 0.06 (s, 6H); **^{13}C NMR** (100 MHz, $CDCl_3$) δ 168.49, 72.67, 70.89, 70.73, 70.62, 70.24, 68.34, 62.72, 60.37, 25.93, 25.45, 21.01, 18.36, 14.18, -5.27. **HRMS-ESI (*m/z*)**: calculated for $C_{19}H_{35}NO_9Si+Na^+$: 472.1973; found: 472.1978.

2,2,3,3-tetramethyl-4,7,10,13-tetraoxa-3-silapentadecan-15-yl

*8-azaspiro[4.5]deca-1,3-diene-8-carboxylate (**1-7**)*

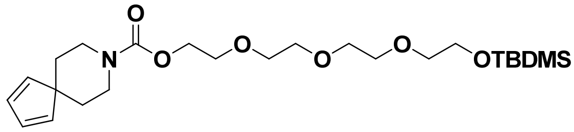

To a solution of **1-2** (0.47 g, 2 mmol) in CH_2Cl_2 (10 mL) was added TFA (0.8 mL) dropwise on ice bath, and the reaction was stirred at room temperature for 0.5 h. The reaction was monitored by TLC. Then, the resulting mixture was concentrated under reduced pressure and 8-azaspiro[4.5]deca-1,3-diene trifluoroacetate was obtained. In the round bottom flask, 0.2 mL of 8-azaspiro[4.5]deca-1,3-diene trifluoroacetate, *N*,*N*-Diisopropylethylamine was added to5 mL CH_2Cl_2) on ice bath. Then, a solution of **1-6** (0.60 g, 1.33 mmol) in CH_2Cl_2 (5 mL) was added, and the reaction was stirred on ice bath at room temperature for 2 h. After reaction, the solvent was removed by vacuum and the residual was purified through column chromatography on silica gel (2:1 petroleum/ethyl acetate) to afford compound **1-7** as yellow oil (300 mg, 48%). **^{1}H NMR** (400 MHz, $CDCl_3$) δ 6.49-6.47 (m, 2H), 6.32-6.31 (m, 2H), 4.27-4.25 (m, 2H), 3.77-3.71 (m, 6H), 3.66-3.65 (d, J = 4 Hz, 9H), 3.57-3.54 (t, J = 5.6 Hz, 3H), 1.56-1.53 (t, J = 5.6 Hz, 4H), 0.89 (s, 9H), 0.06 (s, 6H); **^{13}C NMR** (100 MHz, $CDCl_3$) δ 142.42, 129.62, 72.70, 70.75, 70.71, 70.64, 70.57, 69.68, 64.52, 62.71, 55.28, 43.37, 30.82, 25.93, 18.36, -5.27. **HRMS-ESI (*m/z*)**: calculated for $C_{24}H_{43}NO_6Si{+}Na^+$: 492.2752; found: 492.2760.

2-(2-(2-(2-hydroxyethoxy)ethoxy)ethoxy)ethyl

*8-azaspiro[4.5]deca-1,3-diene-8-carboxylate (**1-8**)*

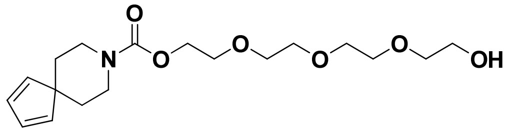

Compound **1-7** (0.25 g, 0.54 mmol) in CH_2Cl_2 (3 mL) was treated with HF-pyridine (0.093 mL, ~0.92 mmol). The reaction was stirred for 30 min at room temperature and then partitioned between ethyl acetate and water. The ethyl acetate layer was dried with Na_2SO_4 and filtered, and the solvent was removed in vacuo. The residue was purified through column chromatography on silica gel (15:1 CH_2Cl_2/MeOH) to afford

compound **1-8** as light yellow oil (124 mg, 65%). **^{1}H NMR** (400 MHz, $CDCl_3$) δ 6.49-6.48 (d, *J* = 4 Hz, 2H), 6.32-6.31 (d, *J* = 4 Hz, 2H), 4.28-4.26 (t, *J* = 4.4 Hz, 2H), 3.74-3.61 (m, 19H), 1.56-1.54 (t, *J* = 5.2 Hz, 4H); **^{13}C NMR** (100 MHz, $CDCl_3$) δ 155.47, 142.42, 129.64, 72.59, 70.66, 70.60, 70.52, 70.35, 69.72, 64.48, 61.75, 55.27, 43.38, 30.83. **HRMS-ESI (*m/z*)**: calculated for $C_{18}H_{29}NO_6+Na^+$: 378.1887; found: 378.1879.

*1-((2,5-dioxopyrrolidin-1-yl)oxy)-1-oxo-2,5,8,11-tetraoxatridecan-13-yl 8-azaspiro[4.5]deca-1,3-diene-8-carboxylate (**1-9**)*

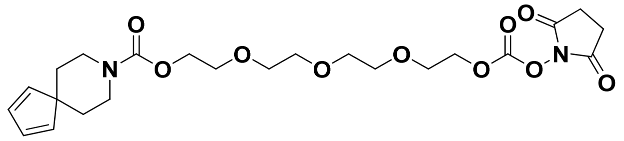

To a solution of **1-8** (0.11g, 0.32 mmol) and *N,N*-disuccinimidyl carbonate (DSC) in CH_3CN (5 mL) was added Et_3N (0.089 mL, 0.64 mmol). The resulting solution was stirred overnight at room temperature. The reaction was monitored by TLC. Upon completion of the reaction, 50 mL CH_2Cl_2 was added and then the mixture was washed twice with 25 mL of 5% aqueous $NaHCO_3$ and water. The organic layer was dried with Na_2SO_4 and filtered, and the solvent was removed under reduced pressure. The crude product was purified through column chromatography on silica gel (50:1 CH_2Cl_2/MeOH) to afford compound **1-9** as colorless oil (0.11 g, 70%). **^{1}H NMR** (400 MHz, $CDCl_3$) δ 6.49-6.48 (d, *J* = 4 Hz, 2H), 6.32-6.31 (d, *J* = 4 Hz, 2H), 4.47-4.52 (t, *J* = 4.4 Hz, 2H), 4.27-4.25 (t, *J* = 4.4 Hz, 2H), 3.80-3.78 (t, *J* = 5.6 Hz, 2H), 3.74-3.71 (t, *J* = 4.4 Hz, 2H), 3.67 (s, 8H), 3.63-3.60 (m, 4H), 2.84 (s, 4H), 1.56-1.53 (t, *J* = 4.8 Hz, 4H); **^{13}C NMR** (100 MHz, $CDCl_3$) δ 168.54, 155.48, 151.65, 142.45, 129.61, 70.92, 70.71, 70.62, 70.54, 70.23, 69.66, 68.35, 64.53, 55.29, 43.37, 30.81, 25.47. **HRMS-ESI (*m/z*)**: calculated for $C_{23}H_{32}N_2O_{10}+Na^+$: 519.1949; found: 519.1956.

*1-((2,5-dioxopyrrolidin-1-yl)oxy)-1,7-dioxo-2,8,11,14,17-pentaoxa-6-azanonadecan-19-yl 8-azaspiro[4.5]deca-1,3-diene-8-carboxylate (**24**)*

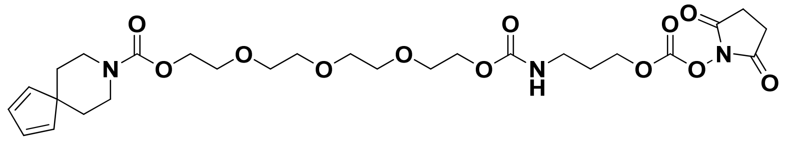

Compound **1-9** (0.05 g, 0.1 mmol) in CH_2Cl_2 (2 mL) was added *N*,*N*-Diisopropylethylamine (0.009 mL, 0.05 mmol). Upon addition of 3-Amino-1-propanol (0.011 g, 0.15 mmol), the solution became turbid immediately and the reaction was stirred for 30 min at room temperature. The reaction was monitored by TLC. Then, 5 mL water was added, and the mixture was extracted twice with 10 mL of CH_2Cl_2. The organic layer was dried with Na_2SO_4 and filtered, and the solvent was removed under reduced pressure. The crude product was purified through column chromatography on silica gel (30:1 CH_2Cl_2/MeOH) to obtain 17-hydroxy-13-oxo-3,6,9,12-tetraoxa-14-azaheptadecyl 8-azaspiro[4.5]deca-1,3-diene-8-carboxylate as a colorless oil (0.035 g, 77%). In the round bottom flask with 2 mL CH_3CN), 17-hydroxy-13-oxo-3,6,9,12-tetraoxa-14-azaheptadecyl 8-azaspiro [4.5] deca-1,3-diene-8-carboxylate (0.035g, 0.08 mmol) and *N*,*N*-disuccinimidyl carbonate (DSC) (0.031 g, 0.12 mmol) was mixed with Et_3N (0.022 mL, 0.16 mmol). The resulting mixture was stirred overnight at room temperature. The reaction was monitored by TLC. Upon completion of the reaction, 10 mL CH_2Cl_2 was added and then the mixture was washed twice with 10 mL of 5% aqueous $NaHCO_3$ and water. The organic layer was dried with Na_2SO_4 and filtered, and the solvent was removed under reduced pressure. The crude product was purified through column chromatography on silica gel (30:1 CH_2Cl_2/MeOH) to afford compound **7** as colorless oil (0.037 g, 77%). **^{1}H NMR** (400 MHz, $CDCl_3$) δ 6.49-6.48 (m, 2H), 6.32-6.31 (m, 2H), 4.42-4.38 (t, *J* = 6 Hz, 2H), 4.27-4.20 (m, 4H), 3.74-3.70 (m, 2H), 3.68-3.57 (m, 15H), 3.32-3.27 (m, 2H), 2.84 (s, 4H), 2.01-1.94 (m, 2H), 1.56-1.53 (m, 4H); **^{13}C NMR** (100 MHz, $CDCl_3$) δ 168.59, 156.48, 155.49, 151.58, 142.42, 129.61, 70.66, 70.59, 70.53, 70.51, 69.66, 69.57, 68.86, 64.53, 64.02, 55.27, 43.37, 37.27, 30.82, 28.83, 25.46. **HRMS-ESI (*m/z*)**: calculated for $C_{27}H_{39}N_3O_{12}+Na^+$: 620.2426; found: 620.2431.

*Phalloidin-diene (**25**)*

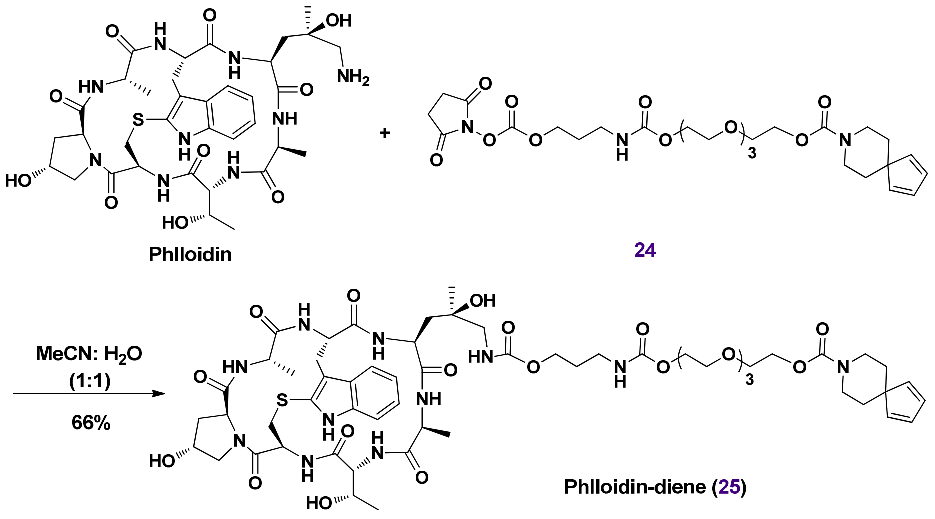

Phalloidin (4 mg, 0.005 mmol) in a mixture (2 mL, MeCN:H_2O = 1:1) was added DIPEA (0.009 mL, 0.05 mmol), and then compound ***24*** (6 mg, 0.01 mmol) was added to the solution. The reaction was monitored by LC-MS, after 15 min, phalloidin was disappeared. The reaction mixture was purified by Semi-preparative liquid chromatography to get the product ***25*** (4.2 mg, 66%).

Conditions: solvent A: H_2O+0.1% TFA; solvent B: CH_3CN; gradient: 5% B to 95% B in 25 min, then 5 min 95% B back to 5% B, Column: C18 column, 10μ, 250*21.2, 19 mL/min flow rate.

HRMS-ESI (*m/z*): calculated for $C_{58}H_{83}N_{11}O_{19}S+H^+$: 1270.5666; found: 1271.0126;

*Taxol-diene (**26**)*

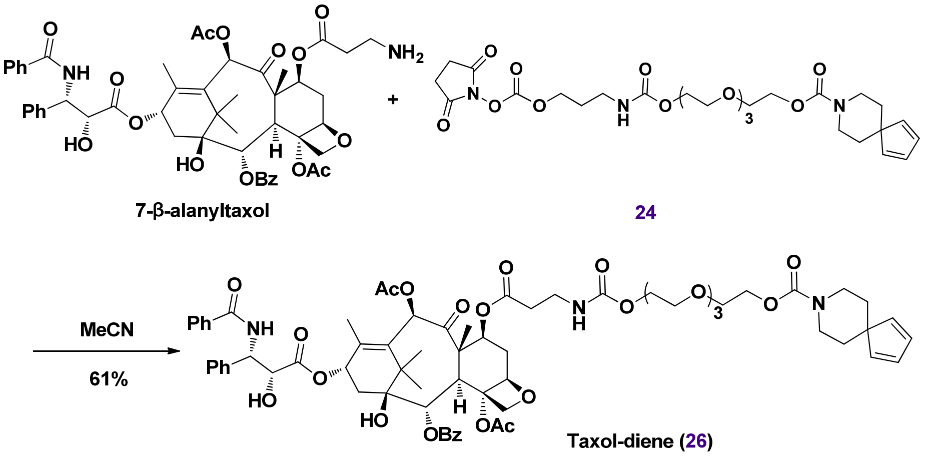

7-β-alanyltaxol (9.24 mg, 0.01 mmol) in MeCN was added DIPEA (0.09 mL, 0.1 mmol), and then compound ***24*** (12 mg, 0.02 mmol) was added to the solution. The reaction was stirred overnight, monitored by TLC. The reaction mixture was purified

by Semi-preparative liquid chromatography to get the product ***26*** (8 mg, 61%). Conditions: solvent A: H_2O+0.1% TFA; solvent B: CH_3CN; gradient: 5% B to 95% B in 25 min, then 10 min 95% B back to 5% B, Column: C18 column, 10μ, 250*21.2, 19 mL/min flow rate.

HRMS-ESI (*m/z*): calculated for $C_{69}H_{83}N_3O_{22}+H^+$: 1306.5541; found: 1307.0124;

3.8 Calculation for fluorescent probes

For further identifying the characterization and fluorescent mechanism of the above fluorescent probes, the density-functional theory (DFT) and time-dependent density functional theory (TD-DFT) methods in Gaussian09 package were carried out with the structure optimization.

8-Ground State:

Number	Atomic Number	X	Y	Z
1	6	-5.500079	0.638758	-0.196947
2	6	-4.132341	0.794338	-0.187026
3	6	-3.370377	-0.387838	-0.225619
4	6	-3.961269	-1.655959	-0.271772
5	6	-5.352459	-1.791592	-0.281132
6	6	-6.097544	-0.626585	-0.243037
7	6	-6.586986	1.663513	-0.163206
8	8	-6.489483	2.869774	-0.120559
9	6	-7.580633	-0.433064	-0.239800
10	8	-8.454904	-1.271825	-0.271847
11	7	-7.788737	0.945559	-0.191029
12	6	-9.110990	1.557146	-0.173580
13	8	-1.399174	0.693481	-0.180051
14	7	-1.933334	-0.397820	-0.220832
15	1	-3.651044	1.763180	-0.151472
16	1	-3.315233	-2.525255	-0.299682
17	1	-5.823502	-2.765460	-0.316303
18	1	-9.657046	1.298623	-1.081700
19	1	-8.979611	2.635688	-0.120352
20	1	-9.672101	1.212317	0.695653

14-Ground State:

Number	Atomic Number	X	Y	Z

1	6	-5.513845	0.574876	-0.156715
2	6	-4.142079	0.725292	-0.121226
3	6	-3.355754	-0.444522	-0.202155
4	6	-3.981984	-1.706143	-0.327735
5	6	-5.366604	-1.826236	-0.357453
6	6	-6.131929	-0.669393	-0.269936
7	6	-6.585788	1.613375	-0.082399
8	8	-6.477764	2.819875	0.019577
9	6	-7.596435	-0.466032	-0.269700
10	8	-8.491712	-1.284902	-0.348035
11	7	-7.794204	0.923564	-0.153568
12	6	-9.097190	1.568892	-0.112738
13	6	-0.948053	0.196972	2.253453
14	6	-0.322244	0.687290	0.970188
15	6	0.279145	-0.610584	0.349310
16	8	-1.454119	0.938189	0.048892
17	7	-1.962118	-0.377416	-0.262592
18	6	-1.097100	-1.317618	0.541235
19	6	-1.419157	-1.030685	1.996883
20	6	0.719726	-0.481163	-1.119586
21	6	2.057393	0.249695	-1.268856
22	7	3.097552	-0.373854	-0.448873
23	6	2.757444	-0.523832	0.968918
24	6	1.426418	-1.264907	1.138628
25	6	4.330207	-0.591529	-0.974201
26	8	4.640436	-0.379102	-2.140212
27	8	5.194182	-1.100775	-0.055834
28	6	6.562379	-1.348483	-0.487413
29	6	9.116625	1.986530	0.489537
30	6	8.872128	0.989592	1.434710
31	6	8.051992	-0.090557	1.112026
32	6	7.464154	-0.186510	-0.154738
33	6	7.718462	0.816327	-1.097373
34	6	8.539735	1.897019	-0.777641
35	1	-3.677045	1.695667	-0.032323
36	1	-3.374088	-2.597954	-0.407570
37	1	-5.828108	-2.802126	-0.448662
38	1	-9.856334	0.790785	-0.161738
39	1	-9.214863	2.245654	-0.960726
40	1	-9.212614	2.132695	0.814245
41	1	-1.079604	0.789890	3.148421
42	1	0.260729	1.603348	0.984425
43	1	-1.186713	-2.333824	0.169401
44	1	-2.021445	-1.660456	2.637433

45	1	-0.030892	0.042200	-1.710053
46	1	0.818145	-1.489786	-1.536338
47	1	1.949657	1.300854	-0.970320
48	1	2.384938	0.234146	-2.305466
49	1	2.698683	0.472367	1.427497
50	1	3.551458	-1.069254	1.469469
51	1	1.548884	-2.291333	0.776086
52	1	1.197820	-1.322598	2.202557
53	1	6.857800	-2.241072	0.063070
54	1	6.561333	-1.560721	-1.554782
55	1	9.756879	2.825666	0.737575
56	1	9.323288	1.050721	2.418738
57	1	7.869476	-0.867588	1.847398
58	1	7.268117	0.748098	-2.081078
59	1	8.731815	2.666277	-1.517295

14-Excitation State (first):

Number	Atomic Number	X	Y	Z
1	6	-5.593668	0.423322	-0.383498
2	6	-4.232734	0.769031	-0.229594
3	6	-3.364251	-0.243319	0.153723
4	6	-3.779274	-1.579422	0.400724
5	6	-5.129111	-1.906279	0.272744
6	6	-6.018860	-0.912805	-0.110805
7	6	-6.741392	1.200769	-0.760892
8	8	-6.867670	2.403865	-1.074207
9	6	-7.481124	-0.994315	-0.318654
10	8	-8.234182	-1.974254	-0.201482
11	7	-7.846749	0.285865	-0.697483
12	6	-9.201543	0.675051	-1.023157
13	6	-0.848701	1.788955	2.008307
14	6	-0.296741	1.481119	0.634440
15	6	0.288831	0.041279	0.792368
16	8	-1.518055	1.179190	-0.194806
17	7	-1.971157	-0.017315	0.277832
18	6	-1.077310	-0.416469	1.412421
19	6	-1.326981	0.633167	2.487864
20	6	0.652221	-0.666126	-0.529607
21	6	1.980363	-0.166522	-1.114338
22	7	3.057305	-0.265401	-0.134699
23	6	2.797831	0.405047	1.137344
24	6	1.482129	-0.088431	1.758503
25	6	4.217330	-0.901279	-0.461738

26	8	4.431604	-1.440863	-1.543657
27	8	5.110540	-0.878350	0.562544
28	6	6.400241	-1.504677	0.320605
29	6	9.333151	1.388561	-0.971419
30	6	9.236703	1.042321	0.378141
31	6	8.289133	0.103204	0.791152
32	6	7.429693	-0.499655	-0.136238
33	6	7.534440	-0.146380	-1.489886
34	6	8.479710	0.792623	-1.904541
35	1	-3.878229	1.778525	-0.394092
36	1	-3.048794	-2.339355	0.652515
37	1	-5.474603	-2.918503	0.459838
38	1	-9.852620	-0.177807	-0.825536
39	1	-9.283750	0.960173	-2.077762
40	1	-9.511015	1.528277	-0.411571
41	1	-0.900339	2.779514	2.441873
42	1	0.237343	2.248945	0.082589
43	1	-1.215667	-1.465685	1.659125
44	1	-1.879953	0.449331	3.400883
45	1	-0.129859	-0.542252	-1.282429
46	1	0.737232	-1.740491	-0.324006
47	1	1.881785	0.879636	-1.439805
48	1	2.257508	-0.759532	-1.985010
49	1	2.754393	1.491252	0.968127
50	1	3.623150	0.211174	1.818791
51	1	1.593026	-1.149389	2.014531
52	1	1.306332	0.454828	2.688970
53	1	6.675617	-1.934532	1.285780
54	1	6.263836	-2.302785	-0.410046
55	1	10.071511	2.116996	-1.295610
56	1	9.899466	1.499680	1.107788
57	1	8.218372	-0.167552	1.842132
58	1	6.866966	-0.608301	-2.211432
59	1	8.554082	1.056494	-2.956137

14-Excitation Wavelength：

Excited State 1: Singlet-A 2.9594 eV 418.95 nm *f* = 0.1343

121 -> 122 0.69804

HOMO: 121

LUMO: 122

14-Emission Wavelength：

Excited State 1: Singlet-A 2.2481 eV 551.52 nm *f* = 0.0871

121 -> 122 0.70226

Excitation Wavelength：418.95nm

Emission Wavelength：551.52nm

Stokes Shift：551.52 – 418.95 = 132.57nm

Fluorescence Quantum Yield：Φ_f = 0.0871/0.1343 = 64.9%

17-Ground State:

Number	Atomic Number	X	Y	Z
1	6	-2.561130	-0.520134	0.008903
2	6	-1.486085	0.339500	0.058623
3	6	-1.739388	1.721000	-0.119151
4	6	-3.065626	2.166412	-0.319881
5	6	-4.131141	1.270286	-0.371681
6	6	-3.868736	-0.081161	-0.206006
7	6	-2.596908	-2.006223	0.161105
8	8	-1.680161	-2.778354	0.363045
9	6	-4.764087	-1.257687	-0.198395
10	8	-5.969213	-1.314819	-0.348348
11	7	-3.934282	-2.373164	0.025354
12	6	-4.407738	-3.746159	0.108252
13	6	1.592765	4.129489	-0.138080
14	6	1.652720	2.626138	-0.061346
15	8	0.451182	2.124633	0.555330
16	7	-0.678948	2.622079	-0.155052
17	6	-0.872112	4.041426	0.147561
18	6	0.435105	4.769560	0.002170
19	6	2.805946	2.158483	0.817066
20	8	2.953638	0.723451	0.791341
21	6	3.740618	0.193038	-0.169437
22	8	4.314144	0.865553	-1.002563
23	6	4.053176	-4.071888	0.007022
24	6	3.244126	-3.408398	0.930315
25	6	3.128396	-2.021576	0.888997
26	6	3.825791	-1.290585	-0.081910
27	6	4.635307	-1.961207	-1.007859
28	6	4.748920	-3.346779	-0.962058
29	1	-0.480864	-0.017186	0.226491
30	1	-3.271564	3.220207	-0.441750
31	1	-5.139239	1.634346	-0.529381
32	1	-5.485483	-3.736994	-0.042003
33	1	-4.180420	-4.168721	1.088298
34	1	-3.937534	-4.357589	-0.663505
35	1	2.520157	4.667439	-0.302714

36	1	1.751182	2.185409	-1.060879
37	1	3.731159	2.628448	0.485692
38	1	2.613424	2.408522	1.859773
39	1	-1.579632	4.445811	-0.579063
40	1	-1.301786	4.187924	1.150018
41	1	0.385623	5.852587	-0.011471
42	1	4.141019	-5.151853	0.042102
43	1	2.703001	-3.971454	1.681683
44	1	2.501181	-1.505669	1.603541
45	1	5.168918	-1.387255	-1.755015
46	1	5.377235	-3.861301	-1.679644

17-Excitation State (first):

Number	Atomic Number	X	Y	Z
1	6	-2.446454	-0.511779	0.069309
2	6	-1.358516	0.367709	0.160166
3	6	-1.615130	1.739150	0.016506
4	6	-2.934498	2.244403	-0.222637
5	6	-4.003899	1.364114	-0.316794
6	6	-3.764693	0.000863	-0.173816
7	6	-2.528561	-1.943638	0.171729
8	8	-1.658738	-2.819048	0.372941
9	6	-4.713148	-1.127667	-0.230782
10	8	-5.941722	-1.113431	-0.418741
11	7	-3.920935	-2.244899	-0.018400
12	6	-4.432639	-3.596812	0.014038
13	6	1.751185	4.121967	-0.244610
14	6	1.787281	2.627226	-0.129741
15	8	0.596262	2.142711	0.569609
16	7	-0.571019	2.657300	0.071220
17	6	-0.699262	4.114048	0.184554
18	6	0.616126	4.793279	-0.067552
19	6	2.959163	2.132646	0.711321
20	8	3.003375	0.698888	0.770919
21	6	3.609879	0.065088	-0.266558
22	8	4.082088	0.662990	-1.217776
23	6	3.704255	-4.195824	0.112773
24	6	3.121214	-3.434032	1.129335
25	6	3.079618	-2.044526	1.026547
26	6	3.624880	-1.411200	-0.100933
27	6	4.209061	-2.179644	-1.119211
28	6	4.248311	-3.567411	-1.011680
29	1	-0.353715	0.007984	0.334625

30	1	-3.109764	3.306808	-0.325508
31	1	-5.008345	1.733933	-0.497313
32	1	-5.506854	-3.554432	-0.170576
33	1	-4.242187	-4.056662	0.989663
34	1	-3.946744	-4.208731	-0.753111
35	1	2.682735	4.629559	-0.476556
36	1	1.808533	2.141518	-1.112366
37	1	3.887884	2.519254	0.286457
38	1	2.852860	2.466146	1.744848
39	1	-1.426993	4.444298	-0.559763
40	1	-1.095957	4.369731	1.178842
41	1	0.580339	5.876865	-0.121109
42	1	3.734009	-5.278609	0.196277
43	1	2.698132	-3.922758	2.001976
44	1	2.628556	-1.451227	1.813673
45	1	4.625399	-1.675902	-1.985174
46	1	4.700639	-4.159222	-1.802027

17-Excitation Wavelength：
Excited State 1:   Singlet-A   3.1867 eV   389.06 nm   *f* = 0.1269
    99 -> 100   0.69391

HOMO: 99
LUMO: 100

17-Emission Wavelength：
Excited State 1:   Singlet-A   2.4623 eV   503.52 nm   *f* = 0.0164
    99 -> 100   0.69881

Excitation Wavelength：389.06nm
Emission Wavelength：503.52nm
Stokes Shift：503.52 – 389.06 = 114.46nm
Fluorescence Quantum Yield：Φ_f = 0.0164/0.1269 = 12.9%

18-Ground State:

Number	Atomic Number	X	Y	Z
1	6	1.772397	0.269599	-0.012796
2	6	0.400703	0.393071	0.001048
3	6	-0.370059	-0.782893	-0.177800
4	6	0.288824	-2.024996	-0.347402
5	6	1.677120	-2.114621	-0.357785
6	6	2.420291	-0.954214	-0.188843
7	6	2.820732	1.322572	0.147659
8	8	2.686183	2.518369	0.322285

9	6	3.877554	-0.722764	-0.145785
10	8	4.793693	-1.515345	-0.255824
11	7	4.043651	0.662308	0.057549
12	6	5.331907	1.329315	0.161900
13	6	-4.368132	-0.186417	-0.675107
14	6	-3.369952	0.934943	-0.560147
15	8	-2.262897	0.510836	0.263618
16	7	-1.751154	-0.706338	-0.250989
17	6	-2.650131	-1.824340	0.086581
18	6	-4.042505	-1.434412	-0.347718
19	6	-3.963509	2.164968	0.105636
20	8	-3.034507	3.243790	-0.005159
21	6	-2.589945	-2.243444	1.564774
22	1	-0.082844	1.348298	0.139753
23	1	-0.288089	-2.929111	-0.481927
24	1	2.156863	-3.076740	-0.492367
25	1	6.108041	0.575578	0.044594
26	1	5.435129	1.809348	1.136519
27	1	5.434608	2.082685	-0.620794
28	1	-5.363838	0.060495	-1.028787
29	1	-2.983011	1.219815	-1.547870
30	1	-4.902887	2.406988	-0.404263
31	1	-4.184687	1.940897	1.155404
32	1	-3.389892	4.001088	0.472233
33	1	-2.341071	-2.664217	-0.541401
34	1	-3.295686	-3.056820	1.749180
35	1	-2.857046	-1.402456	2.207252
36	1	-1.590975	-2.592663	1.834347
37	1	-4.773632	-2.234384	-0.392459

18-Excitation State (first):

Number	Atomic Number	X	Y	Z
1	6	1.790944	0.349274	0.020864
2	6	0.392667	0.451221	0.050419
3	6	-0.351373	-0.729950	-0.089188
4	6	0.277367	-2.005997	-0.264902
5	6	1.662802	-2.094881	-0.296086
6	6	2.412678	-0.931970	-0.154136
7	6	2.824498	1.342192	0.136538
8	8	2.780553	2.581503	0.298156
9	6	3.877991	-0.756762	-0.151781
10	8	4.771689	-1.611952	-0.273350
11	7	4.054342	0.606026	0.024576

12	6	5.349039	1.246410	0.090602
13	6	-4.340507	-0.164470	-0.794364
14	6	-3.368071	0.957829	-0.598458
15	8	-2.265590	0.526541	0.265955
16	7	-1.742158	-0.684763	-0.100745
17	6	-2.671885	-1.819405	0.051368
18	6	-4.025539	-1.418688	-0.479623
19	6	-3.969411	2.168270	0.098966
20	8	-2.999016	3.203625	0.092040
21	6	-2.734680	-2.300288	1.516236
22	1	-0.104174	1.403326	0.176995
23	1	-0.315041	-2.903919	-0.379159
24	1	2.150513	-3.055356	-0.429920
25	1	6.112611	0.469880	0.027938
26	1	5.453673	1.798287	1.030263
27	1	5.473011	1.953420	-0.736924
28	1	-5.312020	0.087991	-1.209825
29	1	-2.920482	1.281694	-1.547656
30	1	-4.876388	2.453776	-0.454164
31	1	-4.266522	1.886200	1.118704
32	1	-3.343486	3.935613	0.627065
33	1	-2.284894	-2.620607	-0.582759
34	1	-3.454748	-3.120850	1.593596
35	1	-3.061645	-1.483889	2.165735
36	1	-1.757573	-2.659706	1.850642
37	1	-4.738328	-2.229142	-0.598495

18-Excitation Wavelength：
Excited State 1: Singlet-A 3.0930 eV 400.85 nm *f* = 0.1312
76 -> 77 0.69471

HOMO: 53
LUMO: 54

18-Emission Wavelength：
Excited State 1: Singlet-A 2.4240 eV 511.48 nm *f* = 0.0359
76 -> 77 0.69929
Excitation Wavelength：400.85nm
Emission Wavelength：511.48nm
Stokes Shift：511.48 – 400.85 = 110.63nm
Fluorescence Quantum Yield：Φ_f = 0.0359/0.1312 = 27.4%

References

#

1. Lin, W., Gupta, A., Kim, K.H., Mendel, D. & Miller, M.J. Syntheses of new spirocarbocyclic nucleoside analogs using iminonitroso Diels-Alder reactions. *Org. Lett.* **11**, 449-52 (2009).
2. Lin, W. *et al.* Diastereoselective synthesis of a spironoraristeromycin using an acylnitroso Diels-Alder reaction. *J. Org. Chem.***74**, 5941-6 (2009).
3. Khan, I., Chidipudi, S.R. & Lam, H.W. Synthesis of spiroindanes by palladium-catalyzed oxidative annulation of non- or weakly activated 1,3-dienes involving C-H functionalization. *Chem. Commun. (Camb)***51**, 2613-6 (2015).
4. Priewisch, B. & Ruck-Braun, K. Efficient preparation of nitrosoarenes for the synthesis of azobenzenes. *J. Org. Chem.***70**, 2350-2 (2005).
5. Molander, G.A. & Cavalcanti, L.N. Nitrosation of aryl and heteroaryltrifluoroborates with nitrosonium tetrafluoroborate. *J. Org. Chem.***77**, 4402-13 (2012).
6. Goswami, L.N., Houston, Z.H., Sarma, S.J., Jalisatgi, S.S. & Hawthorne, M.F. Efficient synthesis of diverse heterobifunctionalized clickable oligo(ethylene glycol) linkers: potential applications in bioconjugation and targeted drug delivery. *Org. Biomol. Chem.***11**, 1116-26 (2013).
7. Asano, S., Gavrilyuk, J., Burton, D.R. & Barbas, C.F., 3rd. Preparation and activities of macromolecule conjugates of the CCR5 antagonist Maraviroc. *ACS Med. Chem. Lett.* **5**, 133-137 (2014).
8. Gavrilyuk, J.I., Wuellner, U. & Barbas, C.F., 3rd. Beta-lactam-based approach for the chemical programming of aldolase antibody 38C2. *Bioorg. Med. Chem. Lett.* **19**, 1421-4 (2009).
9. Kidd, D., Liu, Y. S. & Cravatt, B. F. Profiling serine hydrolase activities in complex proteomes. *Biochemistry*, **2001**, *40*, 4005-4015
10. Riggs-Sauthier J., Wen Z., US patent, *WO 2012051551*.

4. #NMR Spectra of Synthesized Compounds

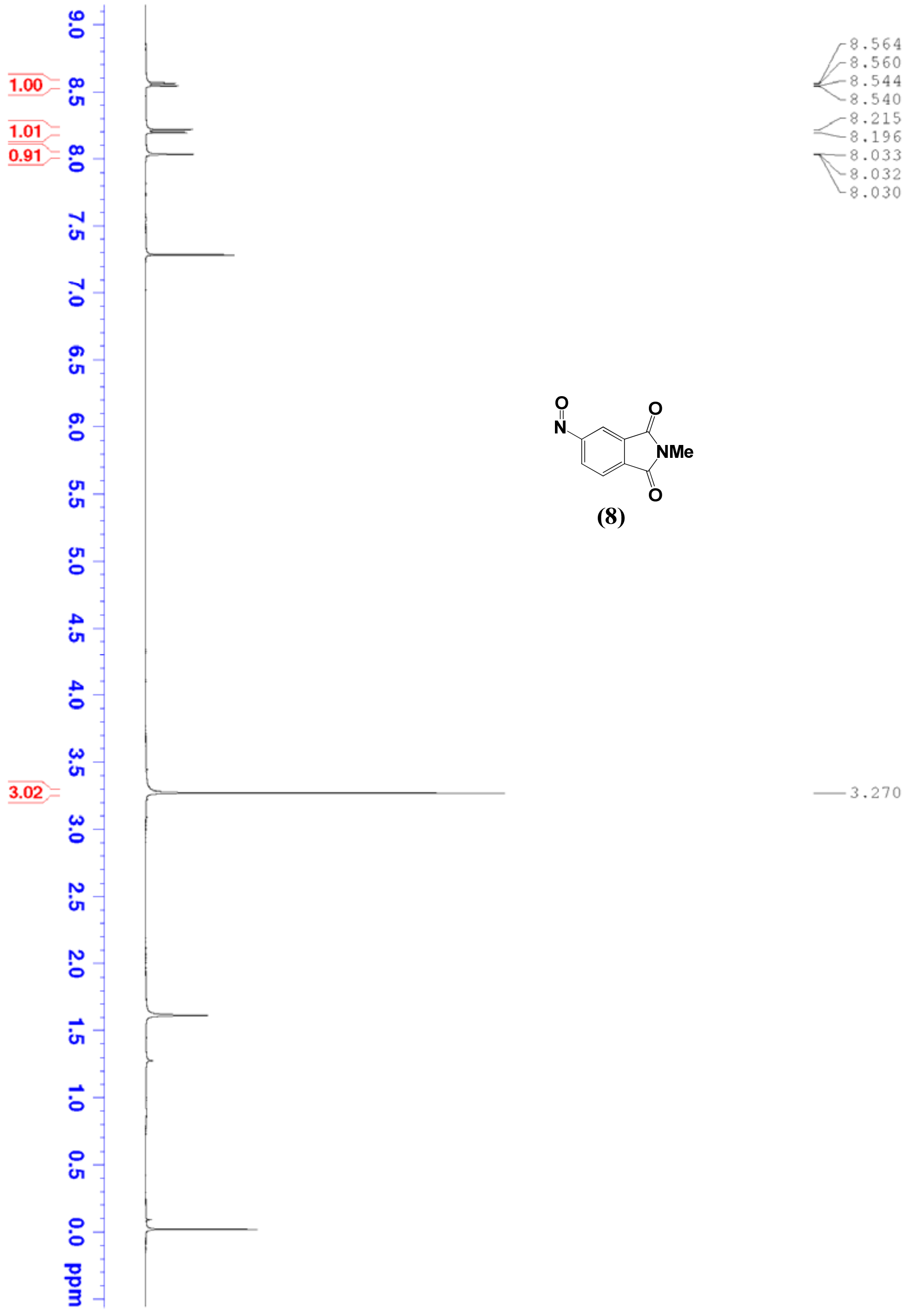

8.564
8.560
8.544
8.540
8.215
8.196
8.033
8.032
8.030
3.270
1.00
1.01
0.91
3.02
9.0
8.5
8.0
7.5
7.0
6.5
6.0
5.5
5.0
4.5
4.0
3.5
3.0
2.5
2.0
1.5
1.0
0.5
0.0
ppm
O
N
O
NMe
O
(8)

#

#

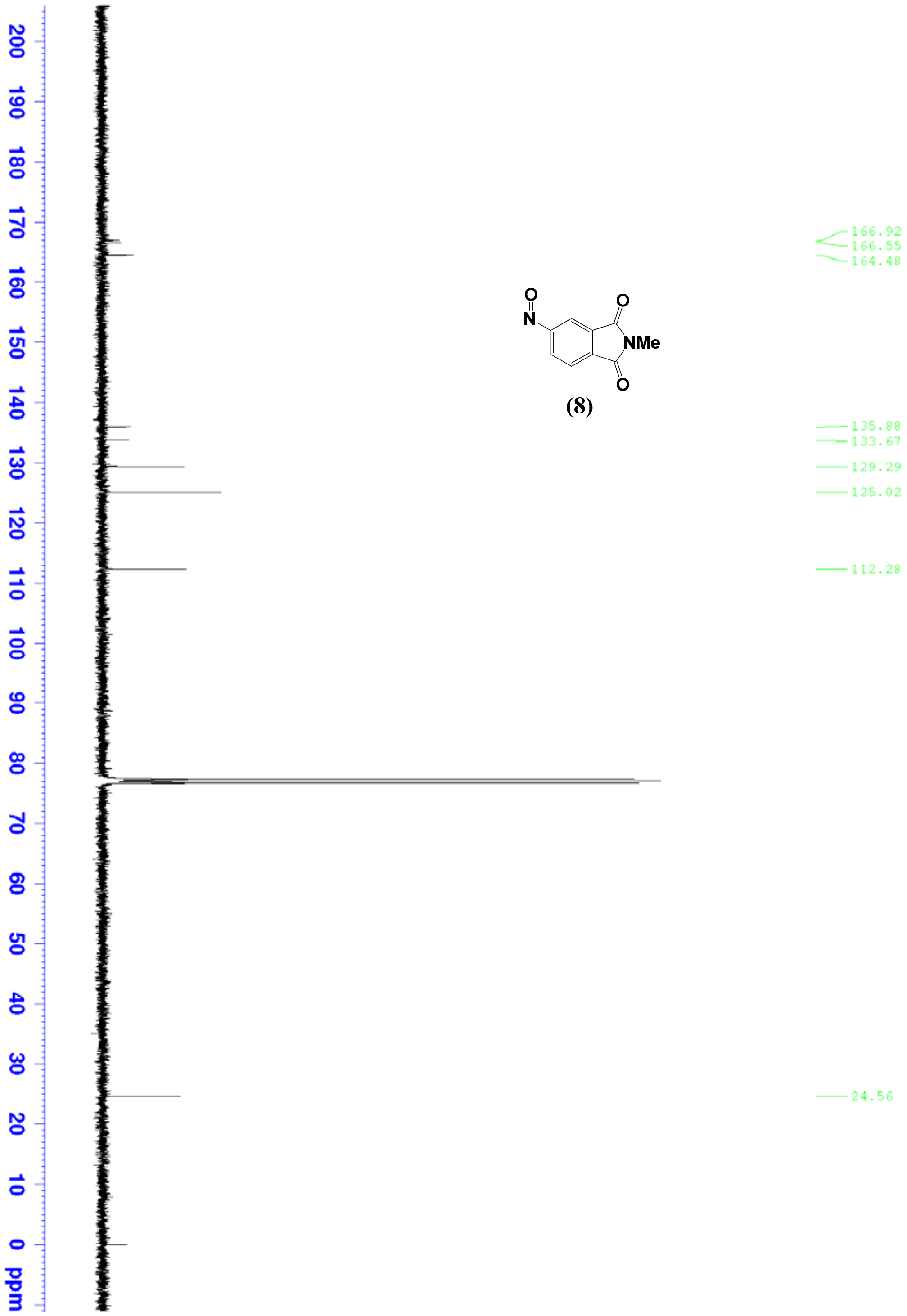
166.92
166.55
164.48
135.88
133.67
129.29
125.02
112.28
24.56
NMe
(8)
ppm

#

#

#

#

#

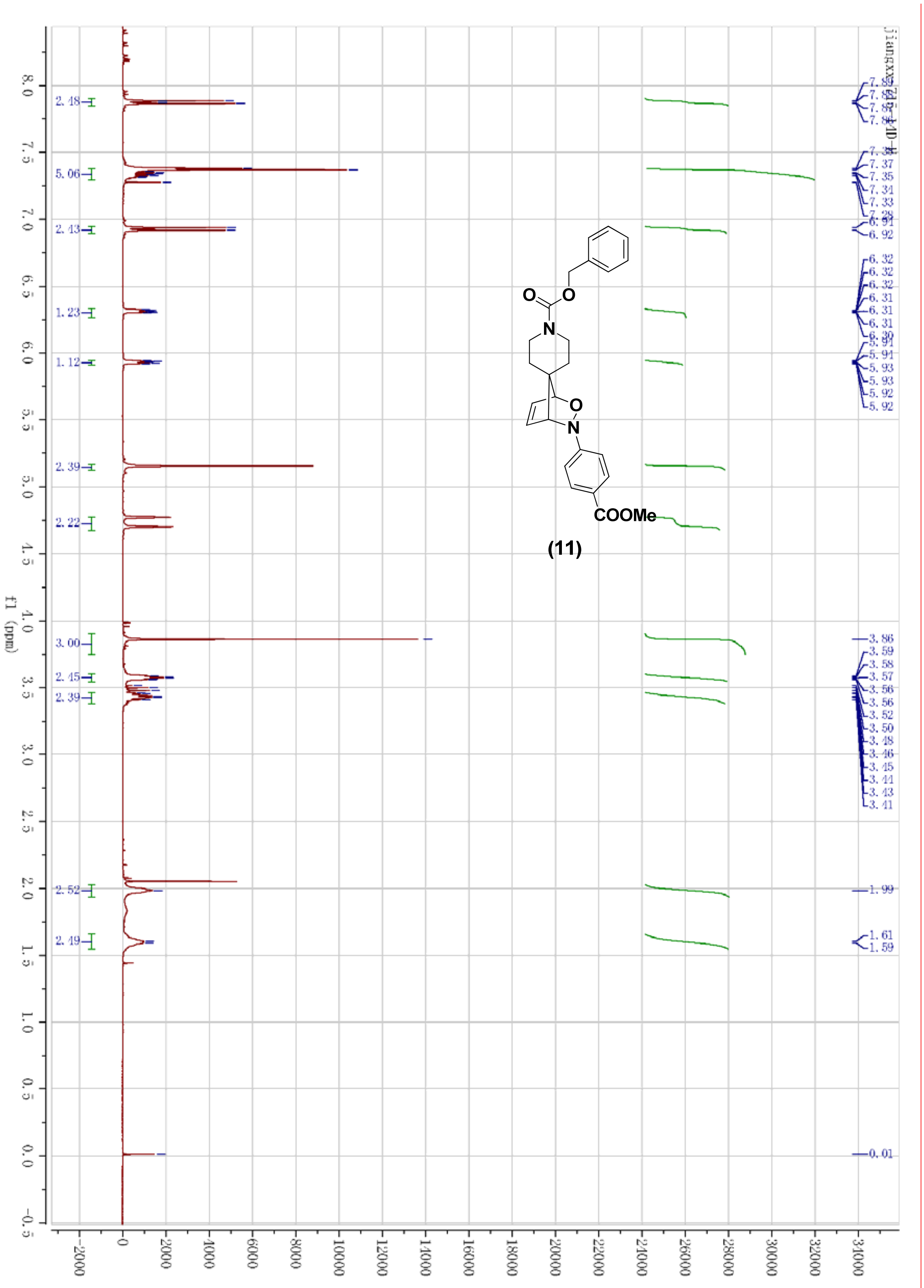

COOMe
(11)
f1 (ppm)

#

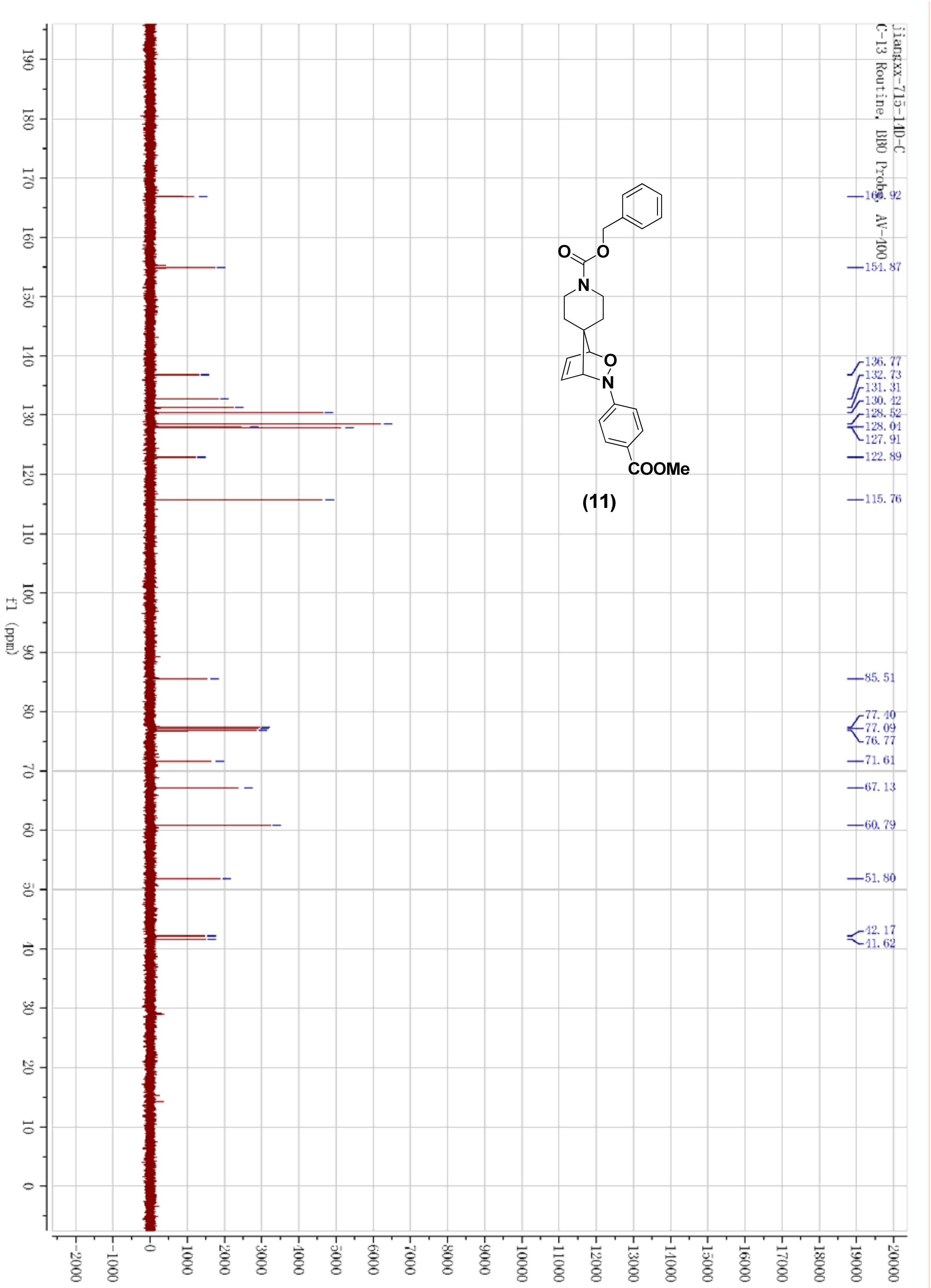

jiangxx-715-14D-C
C-13 Routine, BBO Probe, AV-400
166.92
154.87
136.77
132.73
131.31
130.42
128.52
128.04
127.91
122.89
115.76
85.51
77.40
77.09
76.77
71.61
67.13
60.79
51.80
42.17
41.62
f1 (ppm)
COOMe
(11)

#

#

#

#

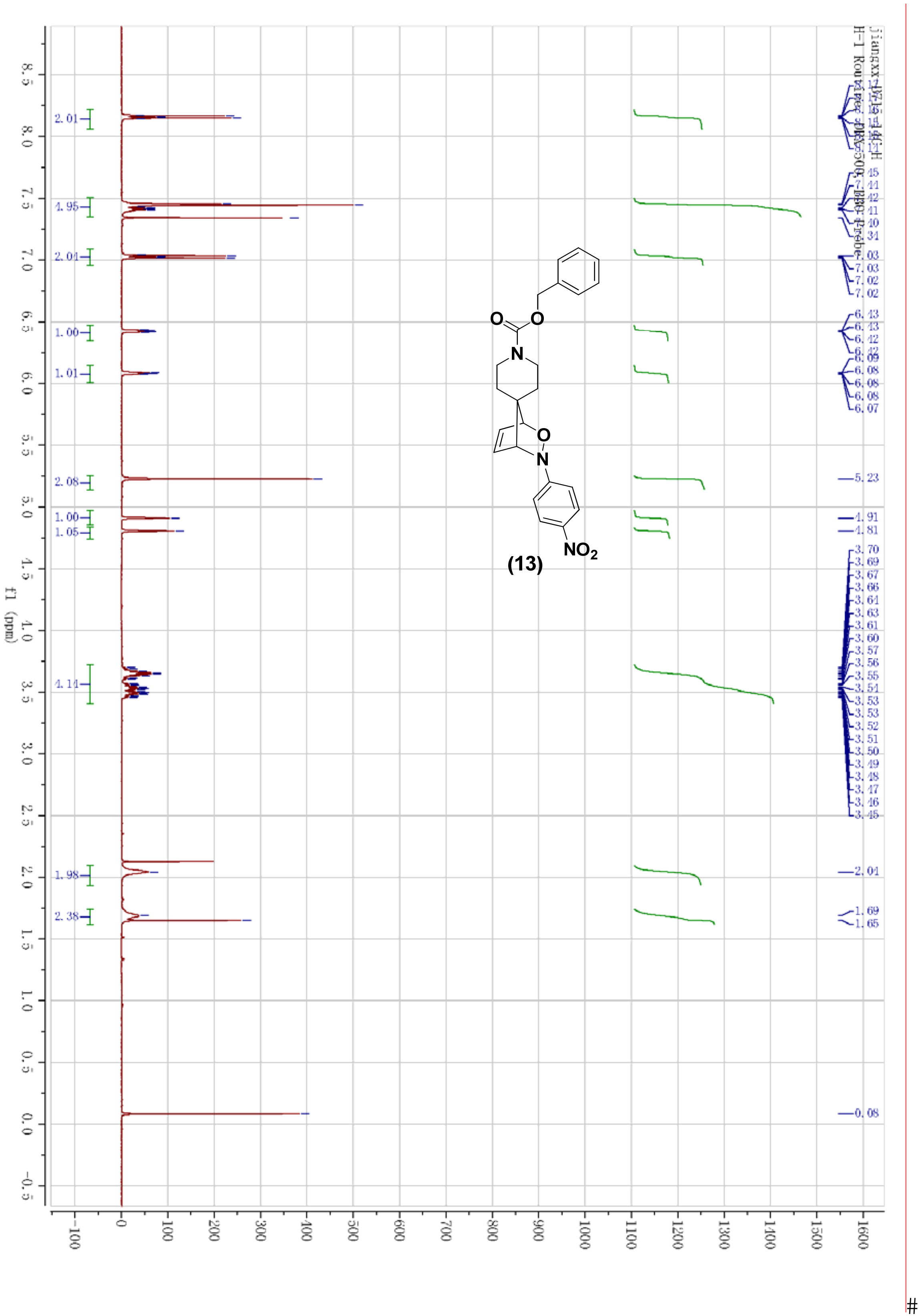
f1 (ppm)
(13)

#

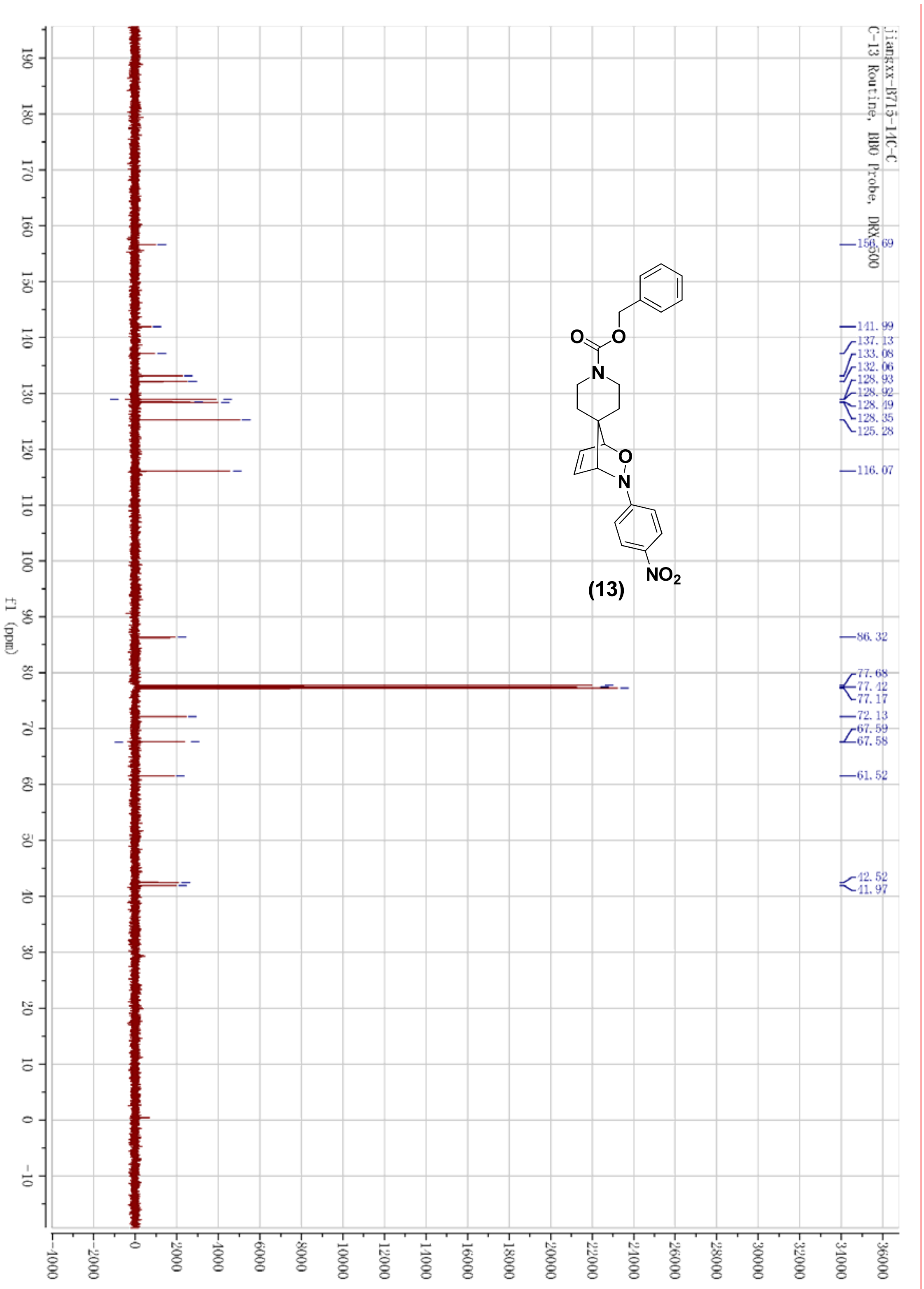
jiangxx-B715-14C-C
C-13 Routine, BBO Probe, DRX-500
156.69
141.99
137.13
133.08
132.06
128.93
128.92
128.49
128.35
125.28
116.07
86.32
77.68
77.42
77.17
72.13
67.59
67.58
61.52
42.52
41.97
f1 (ppm)
(13)
NO2

#

#

#

#

#

#

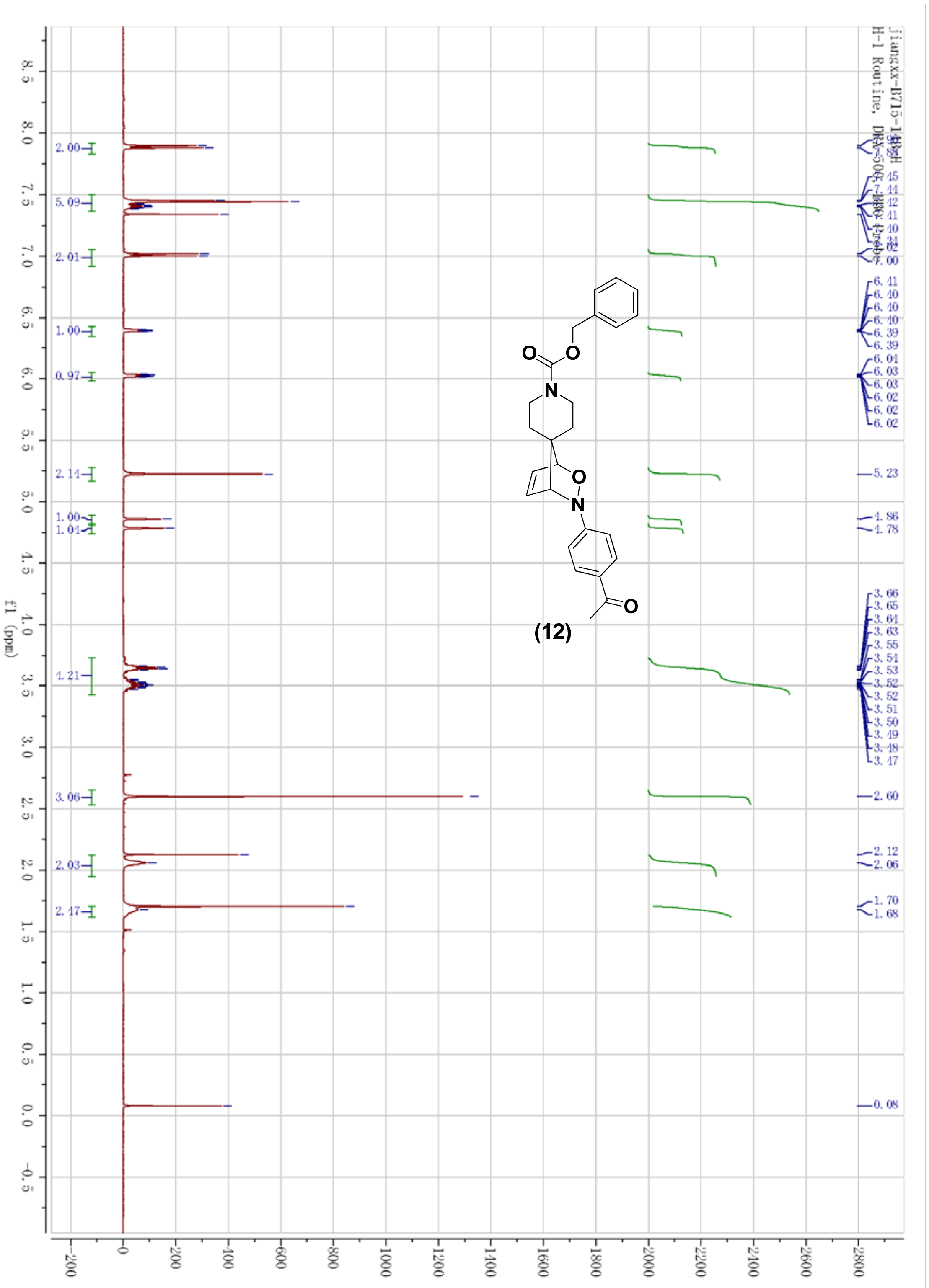
(12)
f1 (ppm)

#

#

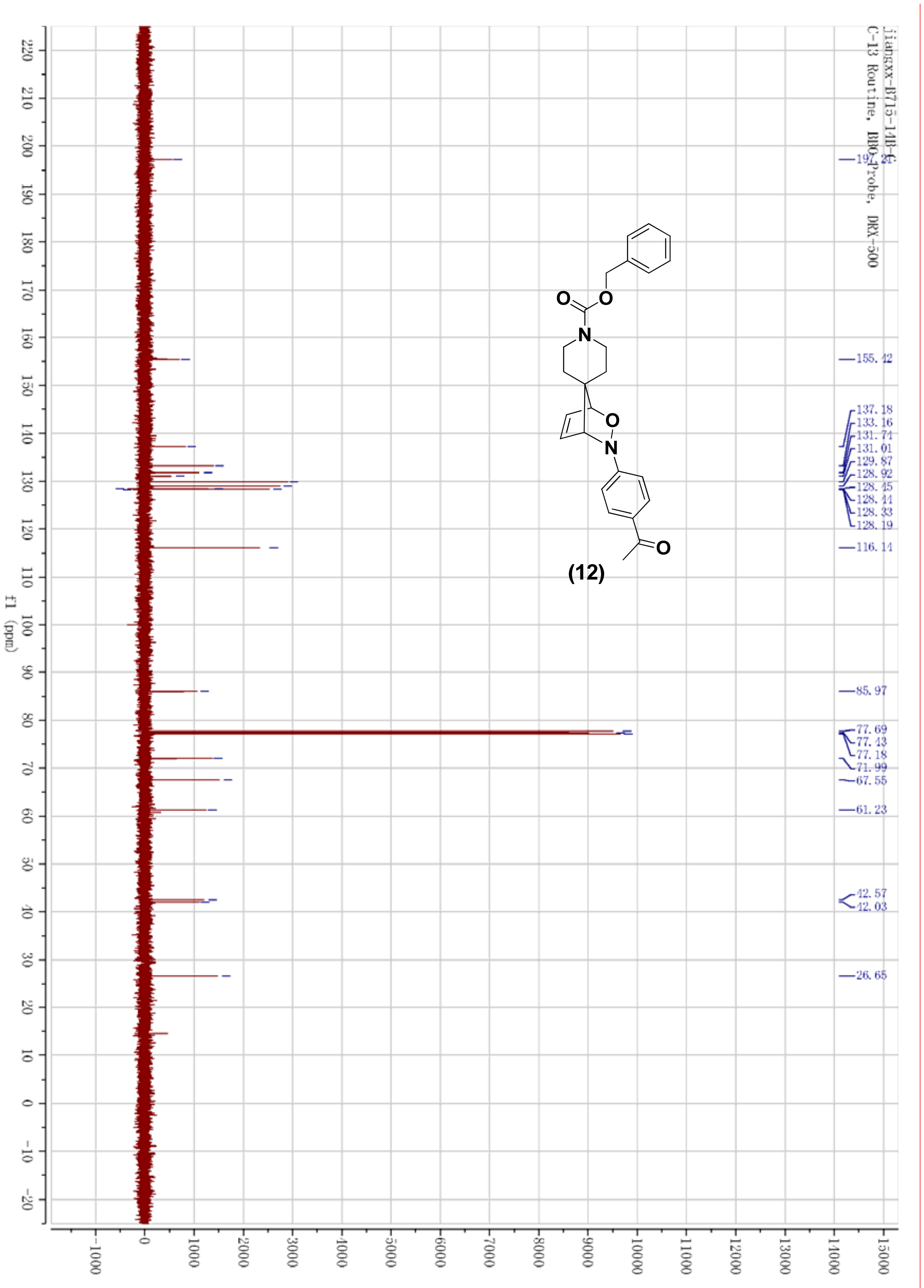

C-13 Routine, BBO Probe, DRX-500
155.42
137.18
133.16
131.74
131.01
129.87
128.92
128.45
128.44
128.33
128.19
116.14
85.97
77.69
77.43
77.18
71.99
67.55
61.23
42.57
42.03
26.65
(12)
f1 (ppm)

#

#

#

#

#

#

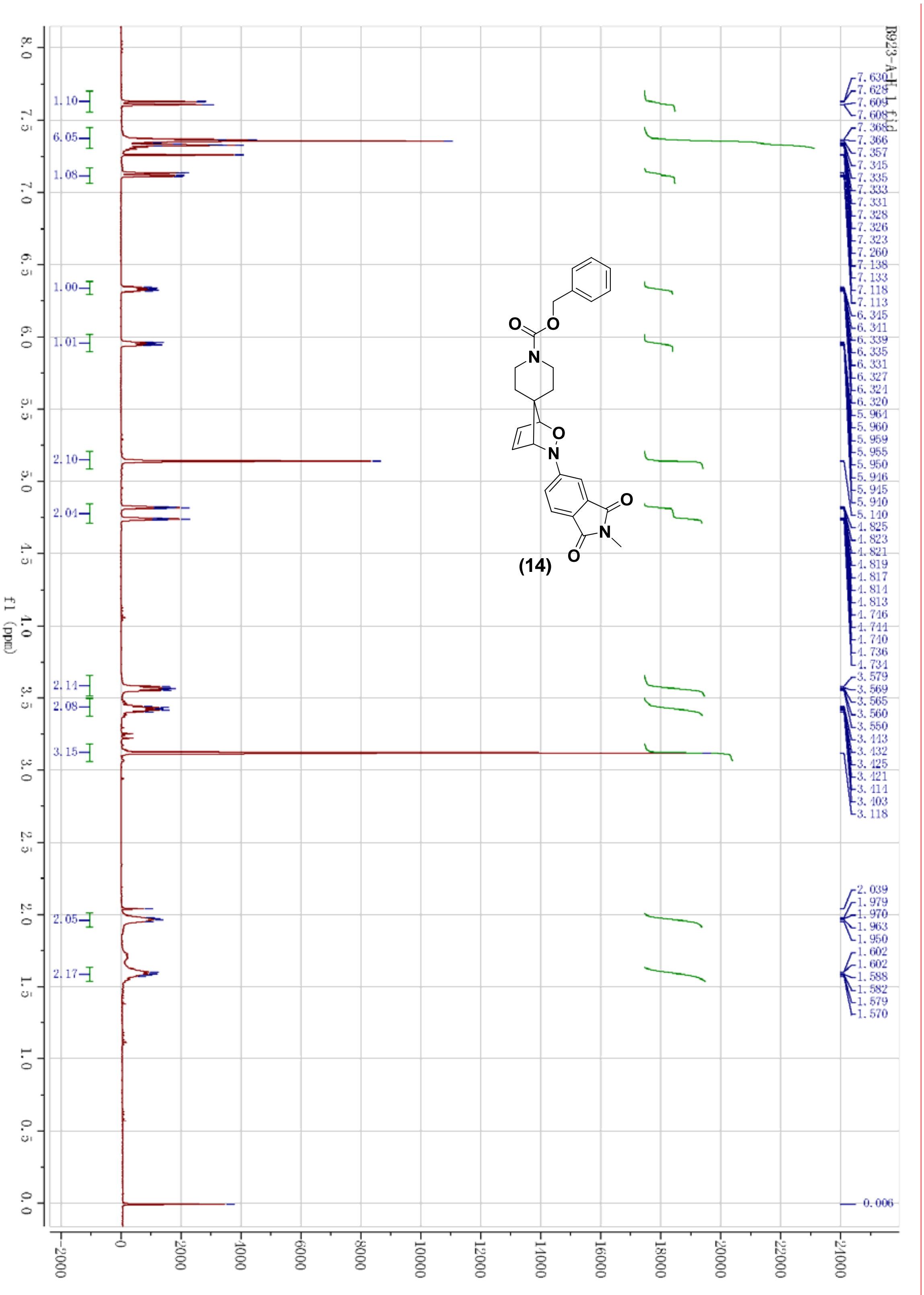
(14)
f1 (ppm)

#

#

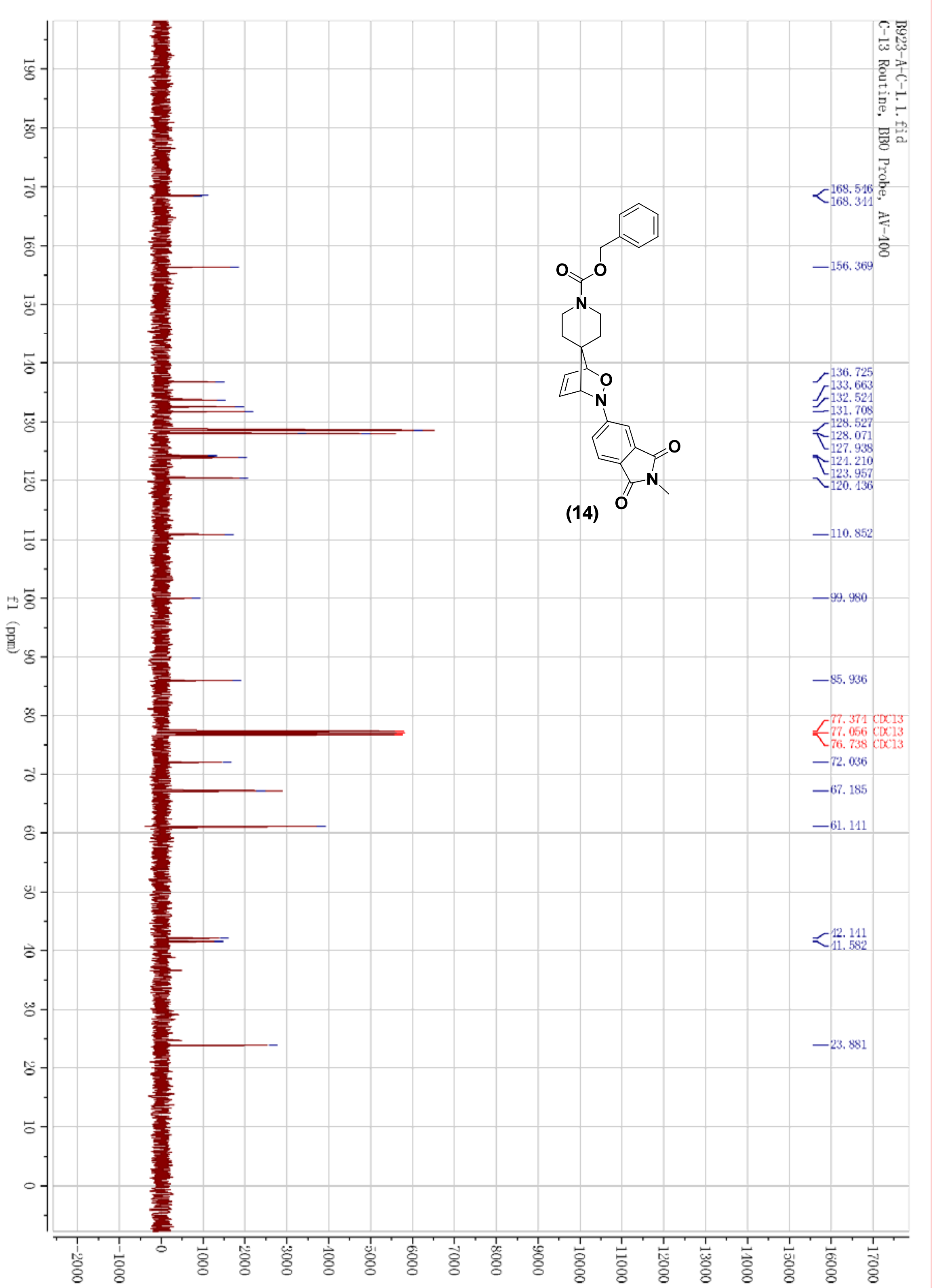
B923-A-C-1.1.fid
C-13 Routine, BBO Probe, AV-400
168.546
168.344
156.369
136.725
133.663
132.524
131.708
128.527
128.071
127.938
124.210
123.957
120.436
110.852
99.980
85.936
77.374 CDCl3
77.056 CDCl3
76.738 CDCl3
72.036
67.185
61.141
42.141
41.582
23.881
(14)
f1 (ppm)

#

#

#

#

#

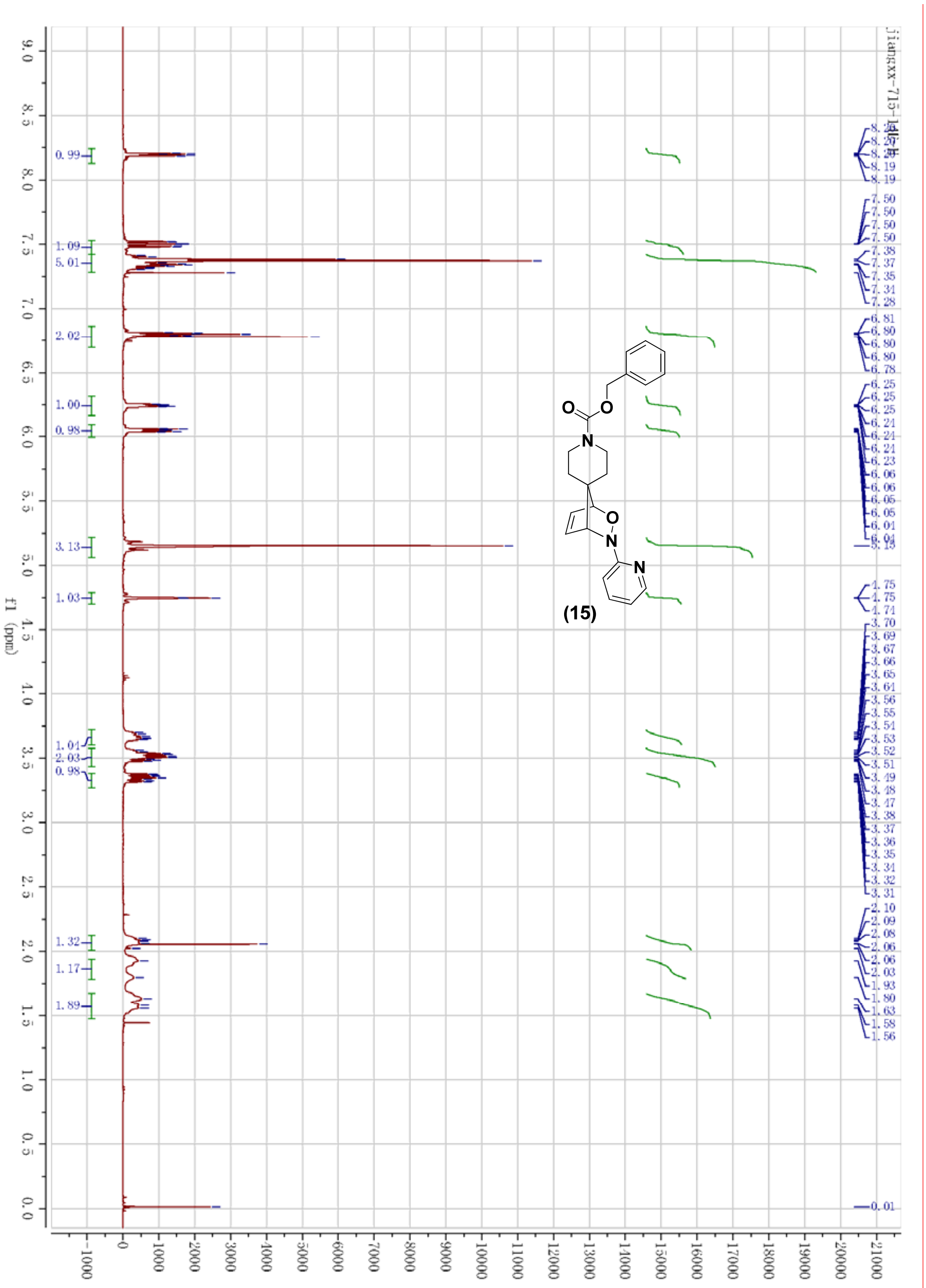
(15)
f1 (ppm)

#

#

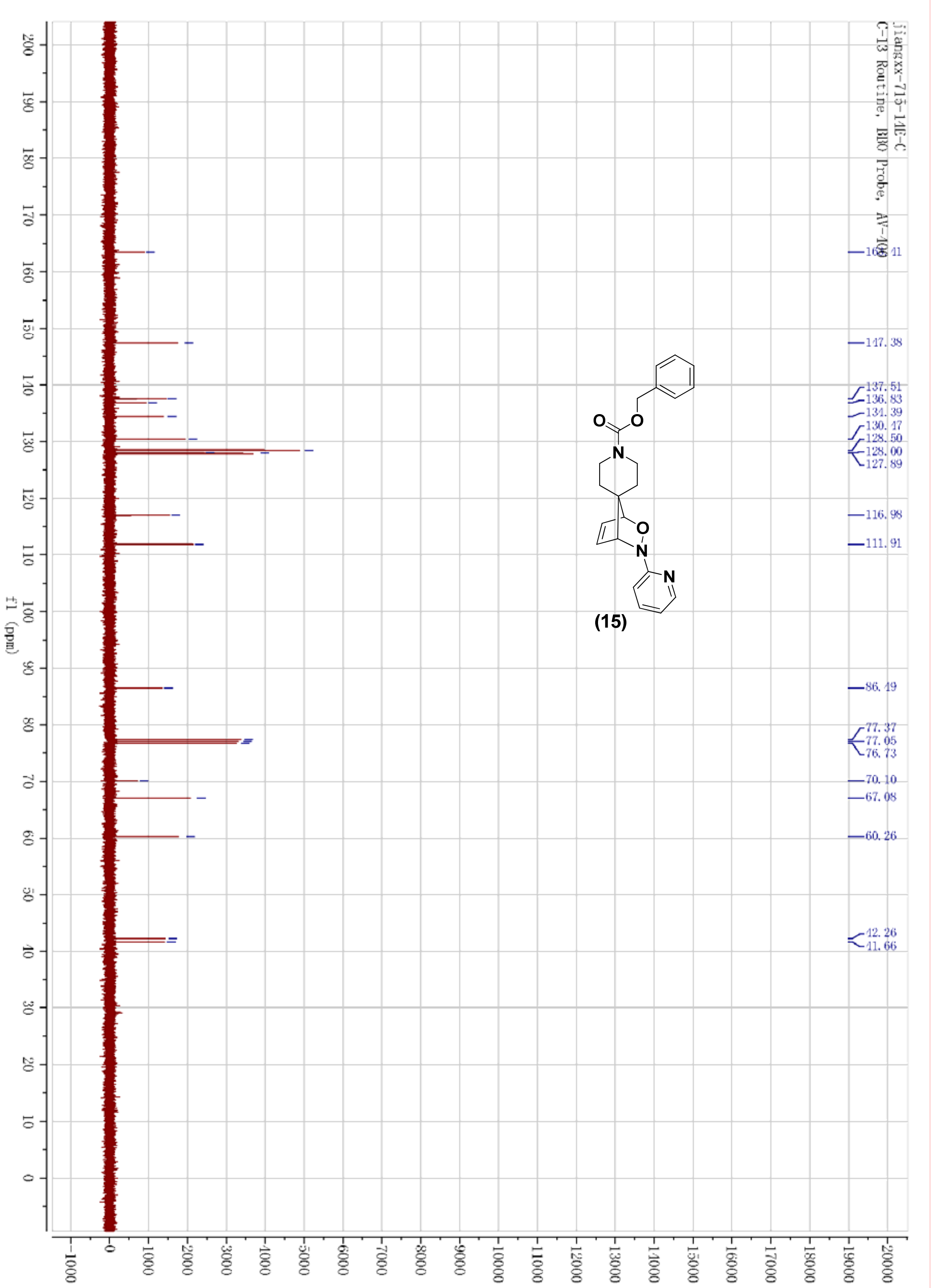
jiangxx-715-1IE-C
C-13 Routine, BBO Probe, AV-400
147.38
137.51
136.83
134.39
130.47
128.50
128.00
127.89
116.98
111.91
86.49
77.37
77.05
76.73
70.10
67.08
60.26
42.26
41.66
f1 (ppm)
(15)

#

#

#

#

#

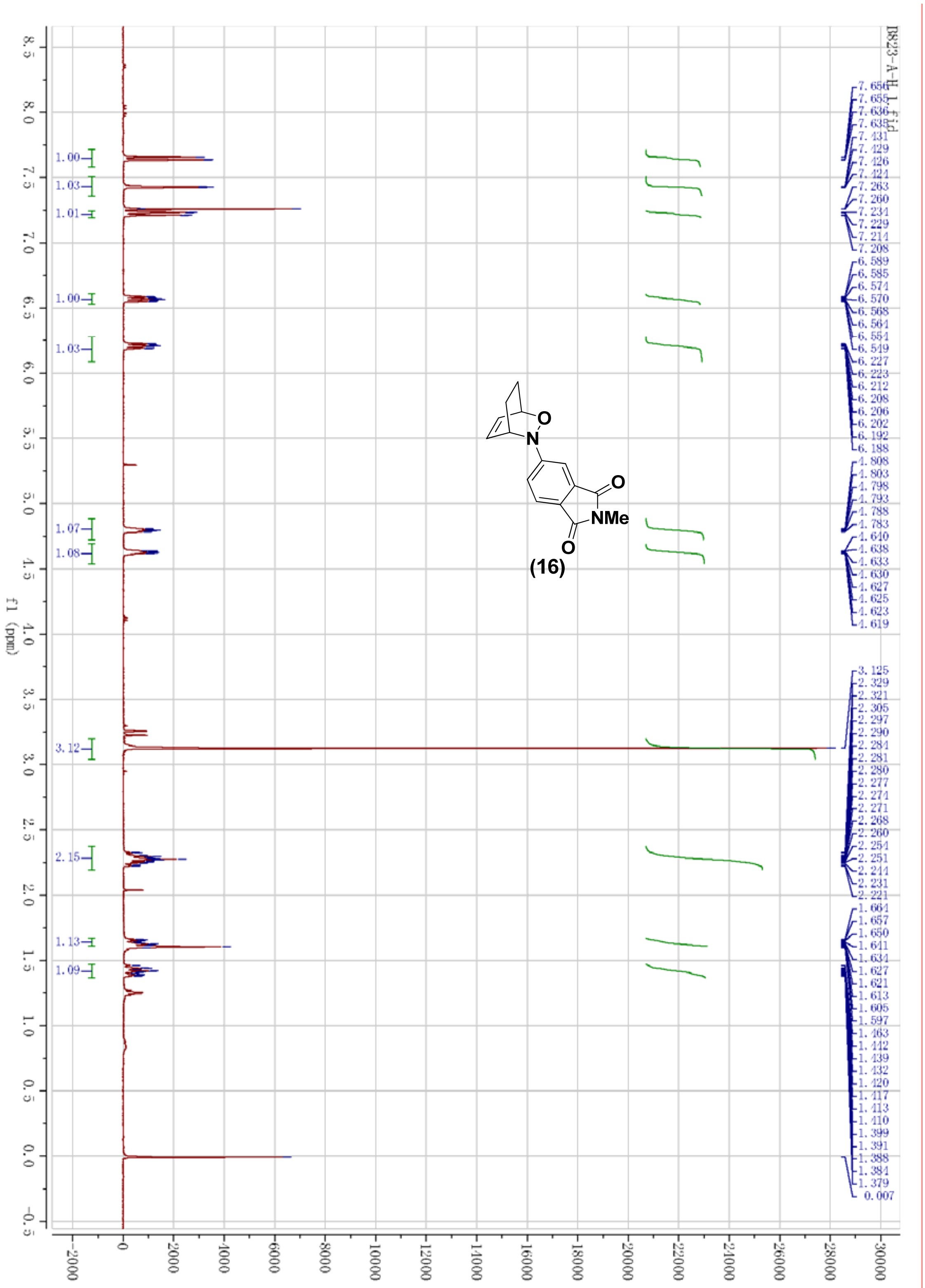

D823-A-H_1.fid
NMe
(16)
f1 (ppm)

#

#

#

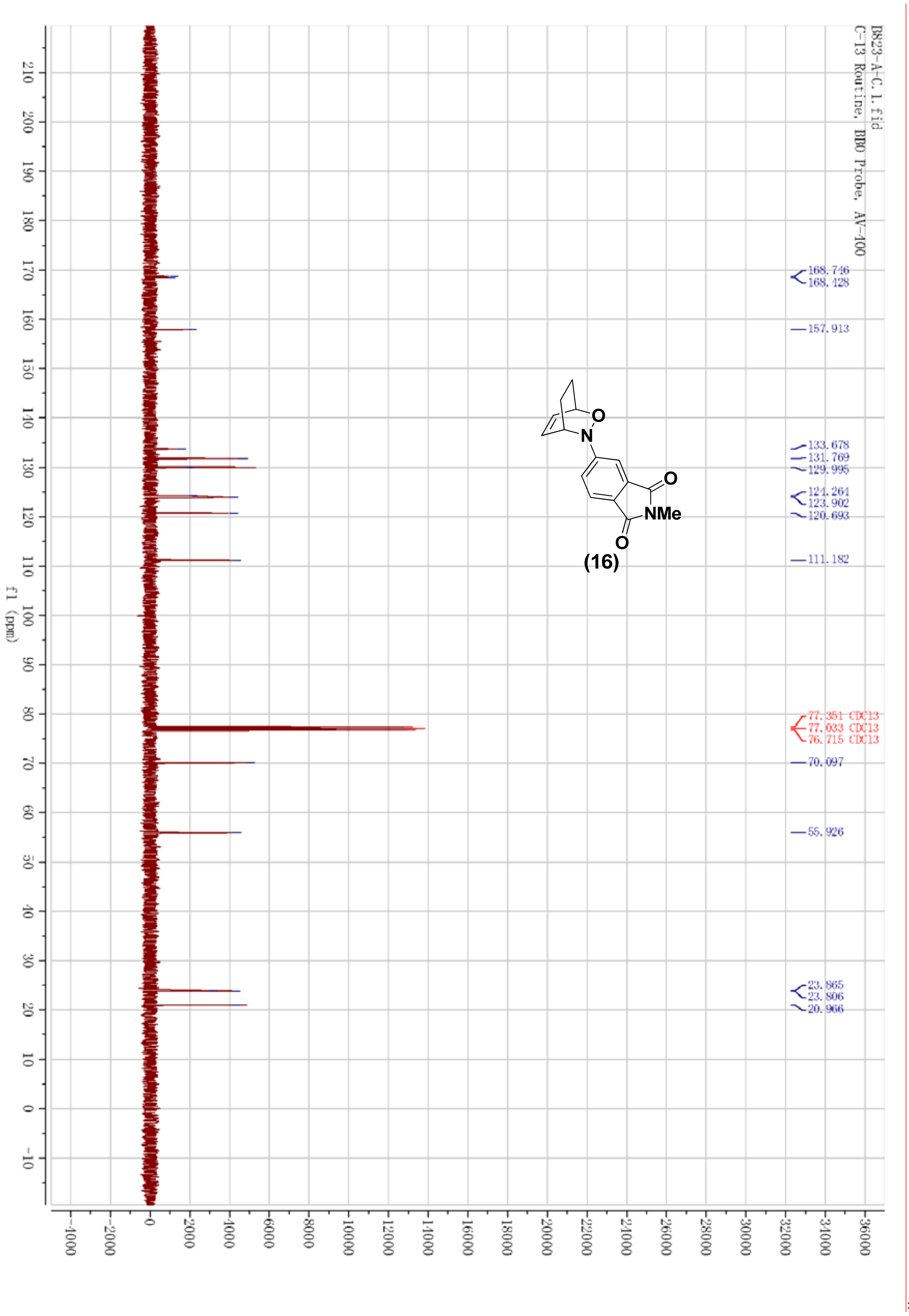

D823-A-C.1.fid
C-13 Routine, BBO Probe, AV-400
168.746
168.428
157.913
133.678
131.769
129.995
124.264
123.902
120.693
111.182
77.351 CDCl3
77.033 CDCl3
76.715 CDCl3
70.097
55.926
23.865
23.806
20.966
O
N
O
NMe
O
(16)
f1 (ppm)

#

#

#

#

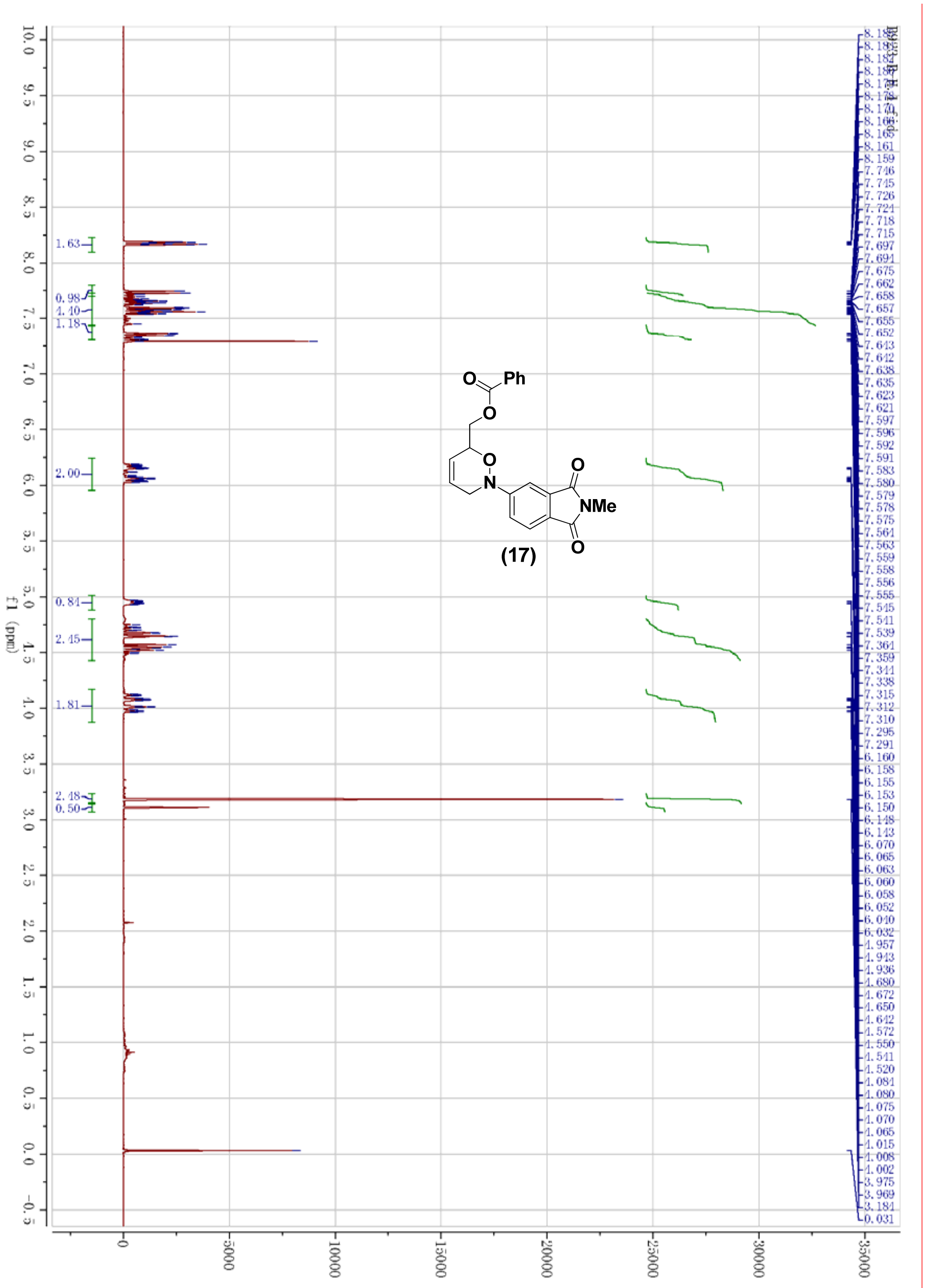
(17)

#

#

#

#

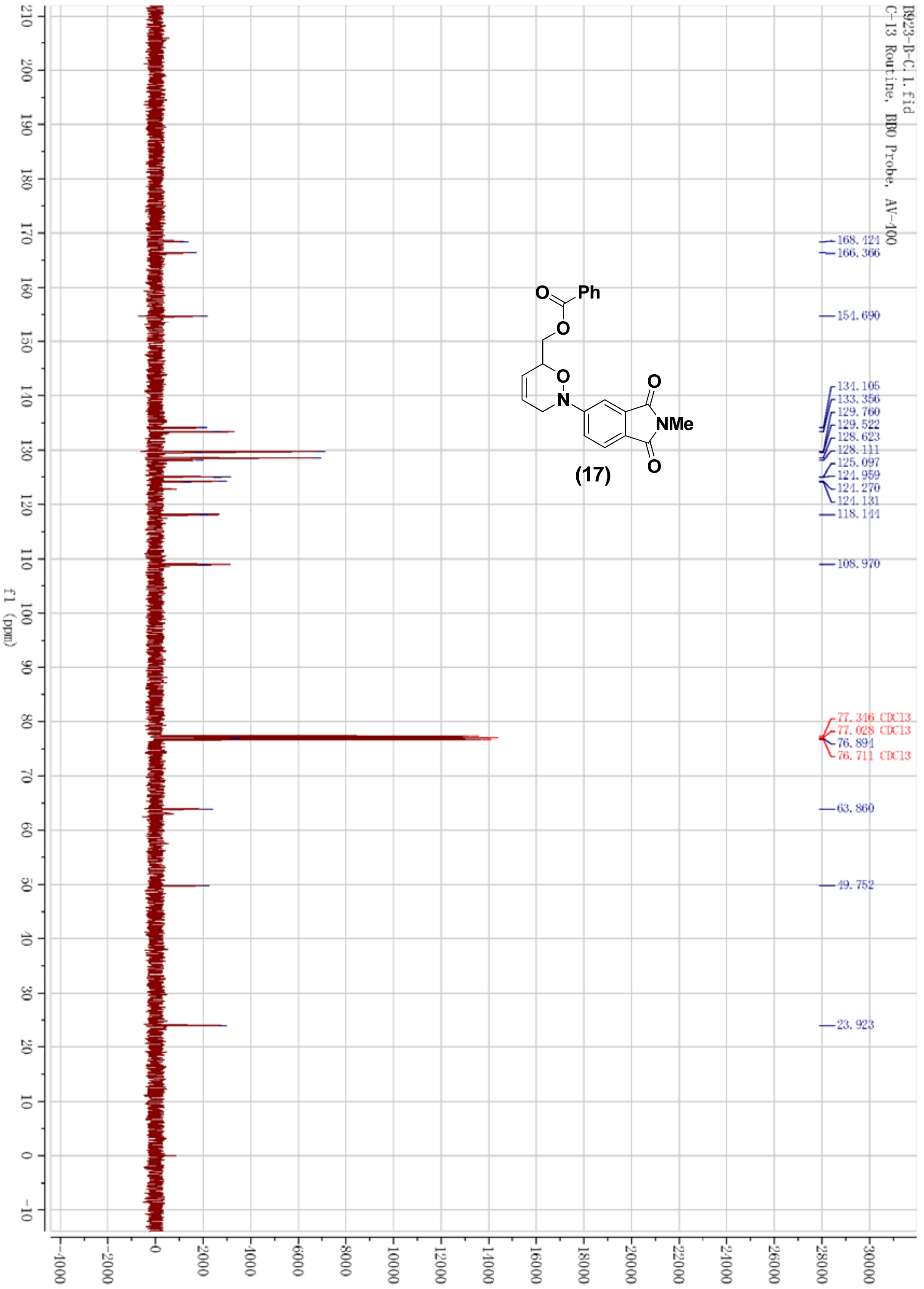

IS923-B-C.1.fid
C-13 Routine, BBO Probe, AV-400
168.424
166.366
154.690
134.105
133.356
129.760
129.522
128.623
128.111
125.097
124.959
124.270
124.131
118.144
108.970
77.346 CDCl3
77.028 CDCl3
76.894
76.711 CDCl3
63.860
49.752
23.923
O
Ph
O
O
N
O
NMe
O
(17)
f1 (ppm)

#

#

#

#

#

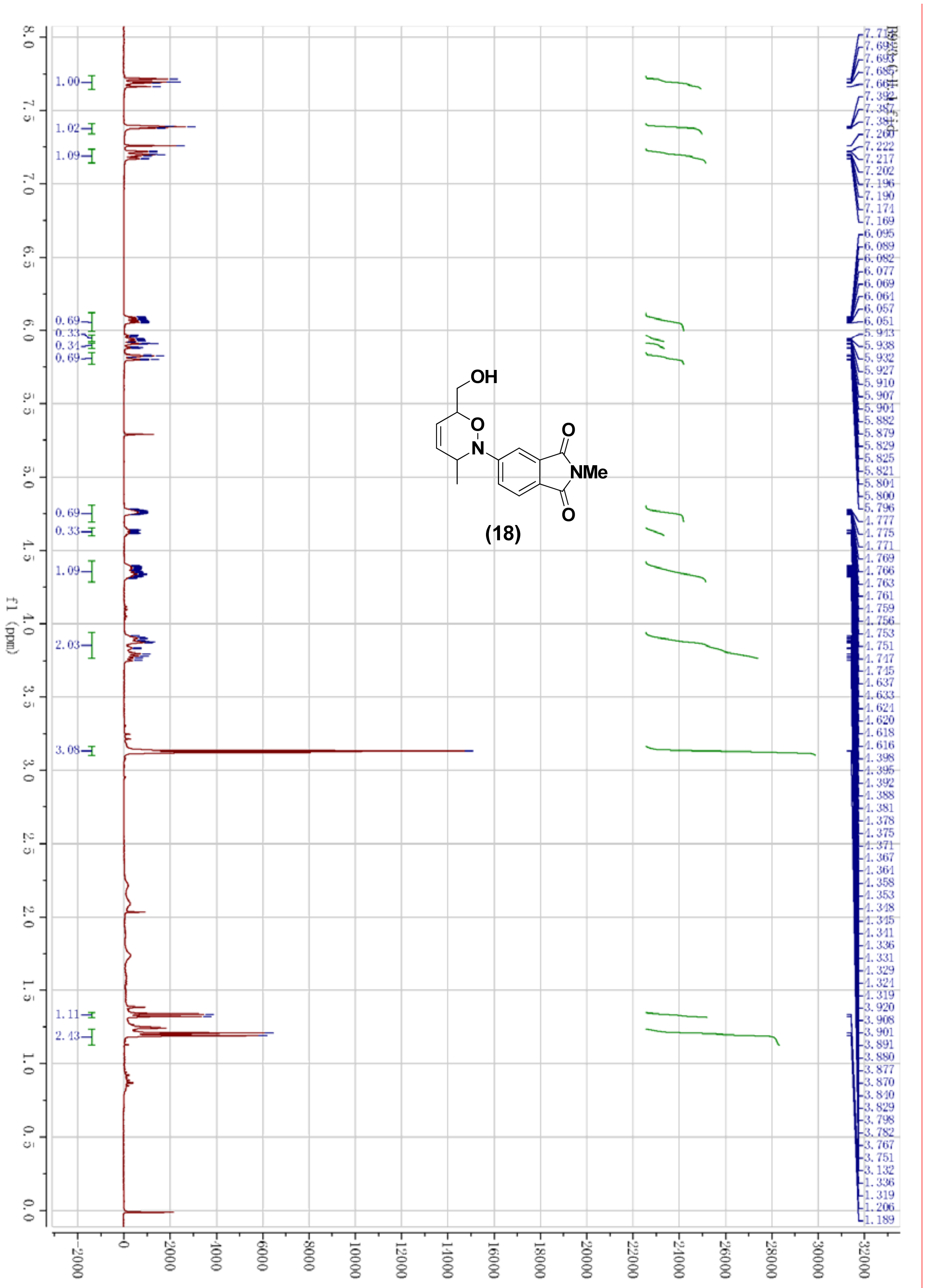
OH
O
N
O
NMe
O
(18)

#

#

#

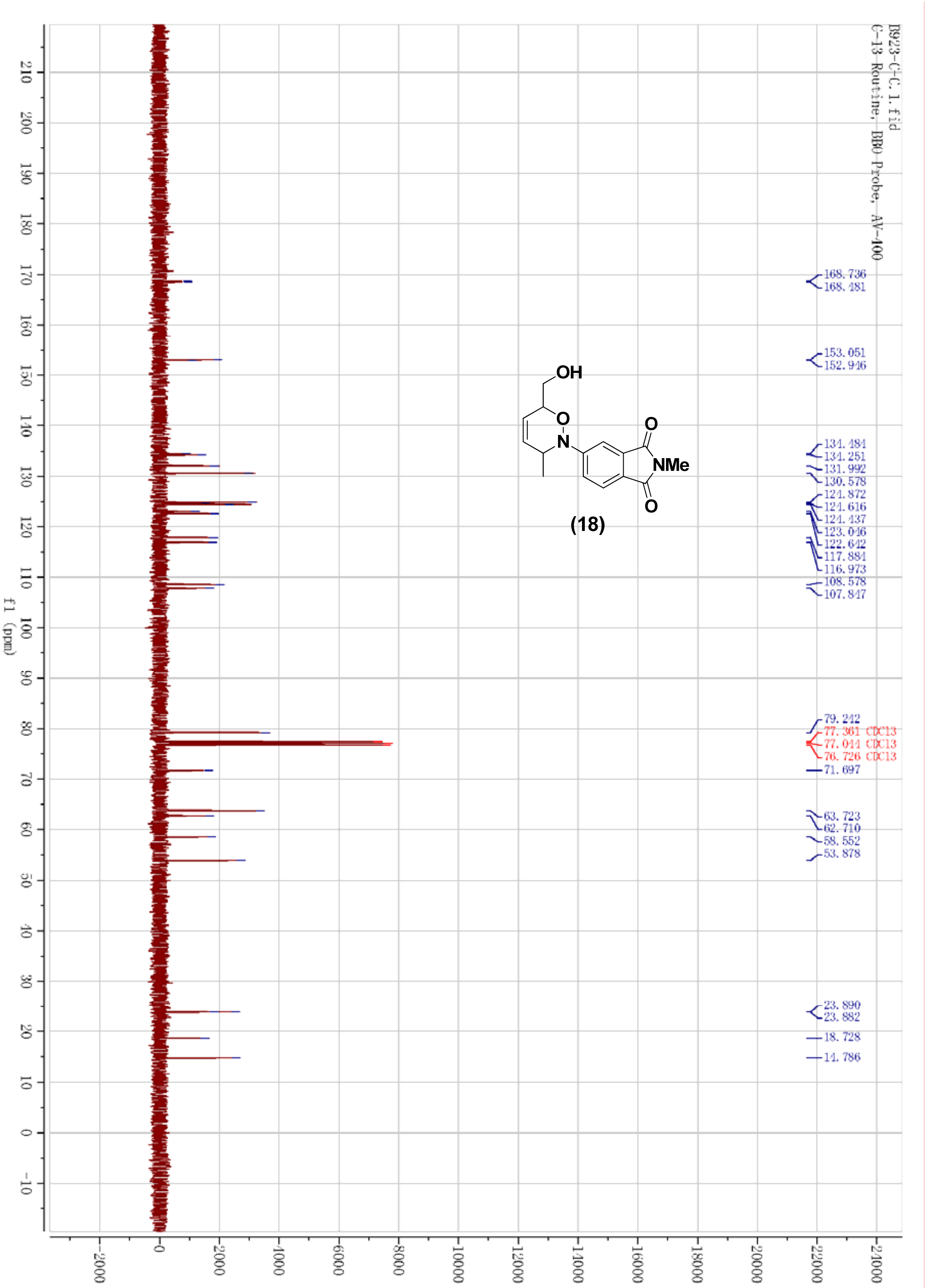

B923-C-C.1.fid
C-13 Routine, BBO Probe, AV-400
168.736
168.481
153.051
152.946
134.484
134.251
131.992
130.578
124.872
124.616
124.437
123.046
122.642
117.884
116.973
108.578
107.847
79.242
77.361 CDCl3
77.044 CDCl3
76.726 CDCl3
71.697
63.723
62.710
58.552
53.878
23.890
23.882
18.728
14.786
OH
O
N
O
NMe
O
(18)
f1 (ppm)

#

#

#

#

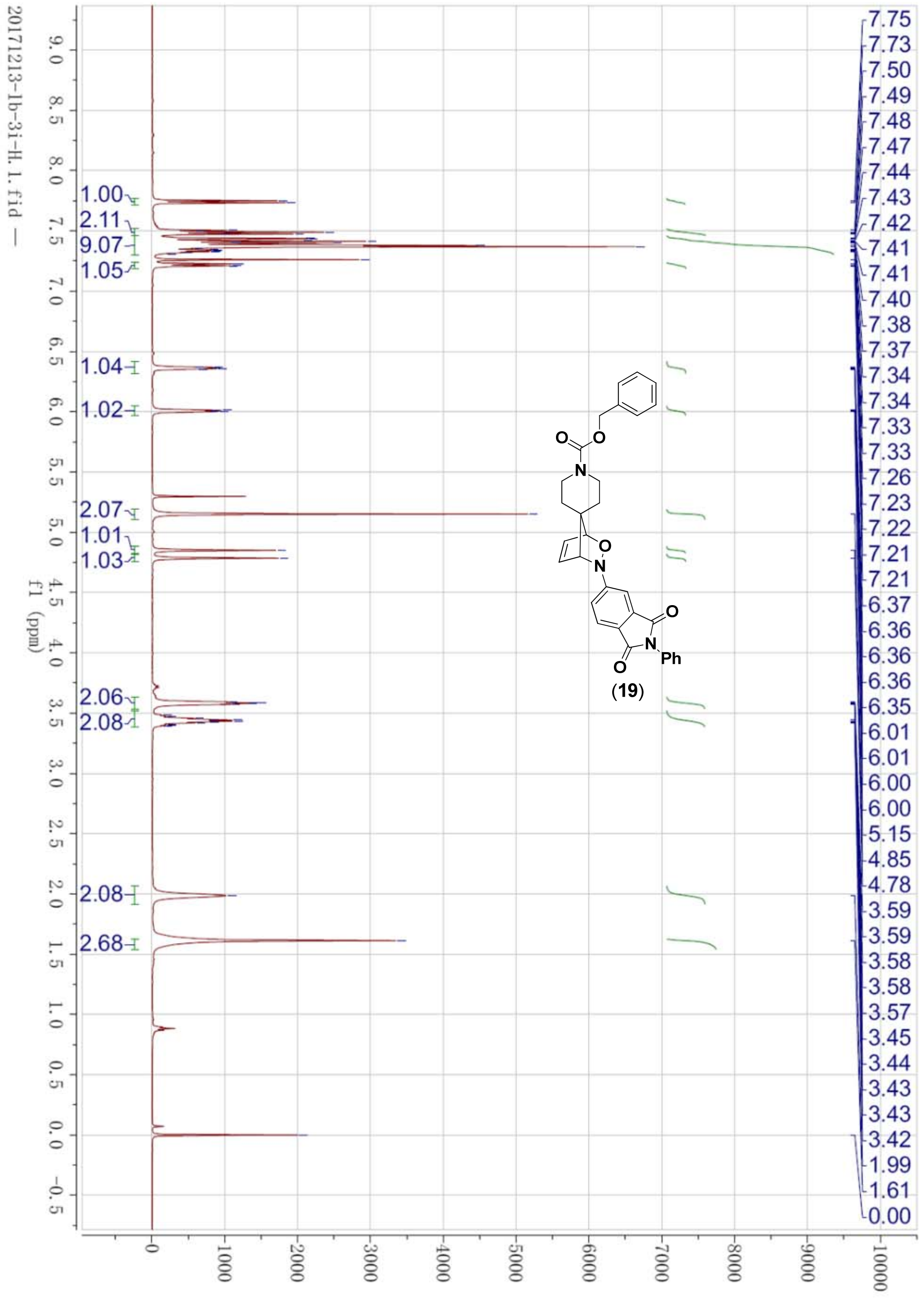

20171213-lb-3i-H.1.fid
7.75 7.73 7.50 7.49 7.48 7.47 7.44 7.43 7.42 7.41 7.41 7.40 7.38 7.37 7.34 7.34 7.33 7.33 7.26 7.23 7.22 7.21 7.21 6.37 6.36 6.36 6.36 6.35 6.01 6.01 6.00 6.00 5.15 4.85 4.78 3.59 3.59 3.58 3.58 3.57 3.45 3.44 3.43 3.43 3.42 1.99 1.61 0.00
1.00
2.11
9.07
1.05
1.04
1.02
2.07
1.01
1.03
2.06
2.08
2.08
2.68
f1 (ppm)
O
N
Ph
(19)

#

#

#

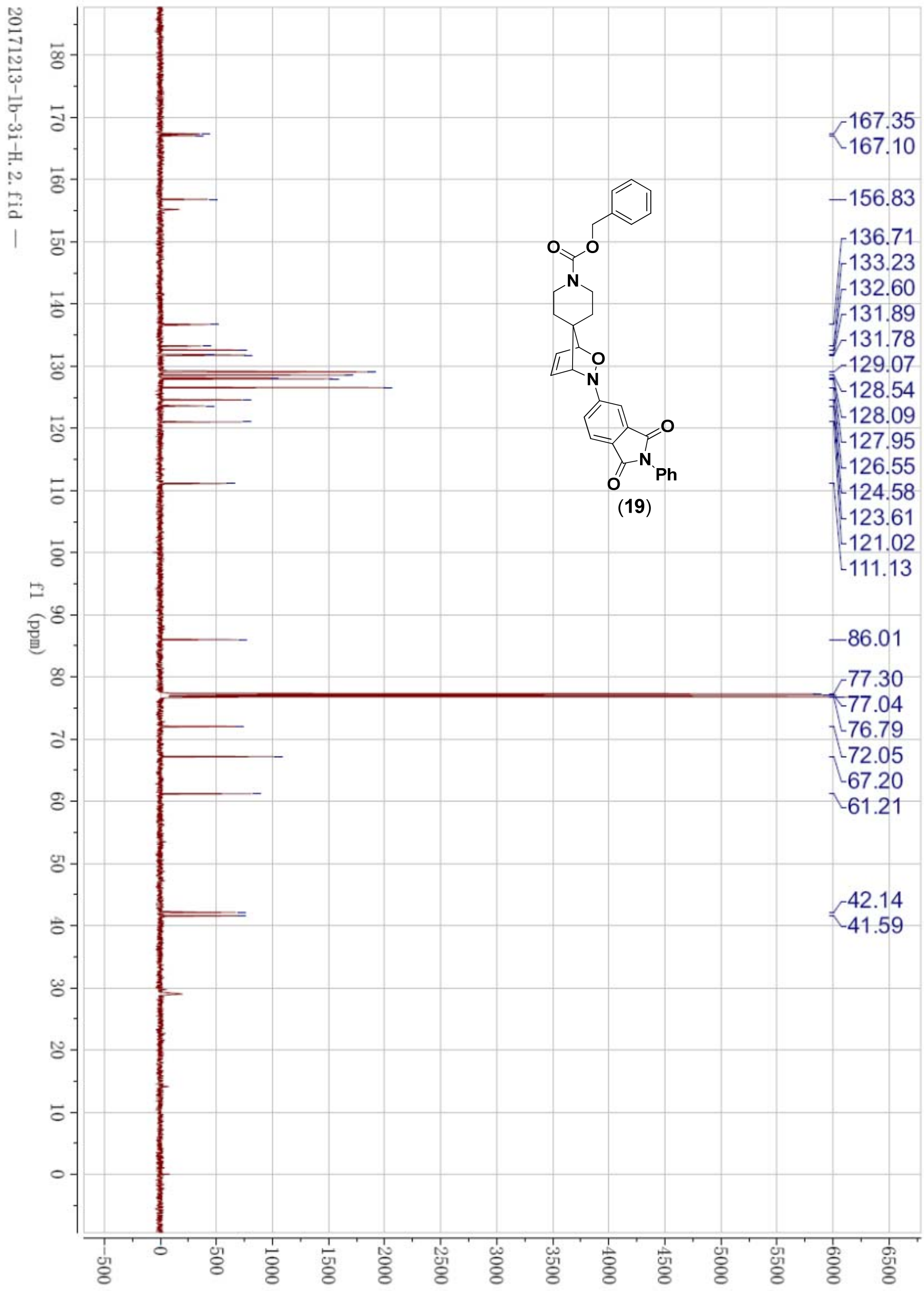
20171213-1b-3i-H.2.fid —
167.35
167.10
156.83
136.71
133.23
132.60
131.89
131.78
129.07
128.54
128.09
127.95
126.55
124.58
123.61
121.02
111.13
86.01
77.30
77.04
76.79
72.05
67.20
61.21
42.14
41.59
(19)
f1 (ppm)

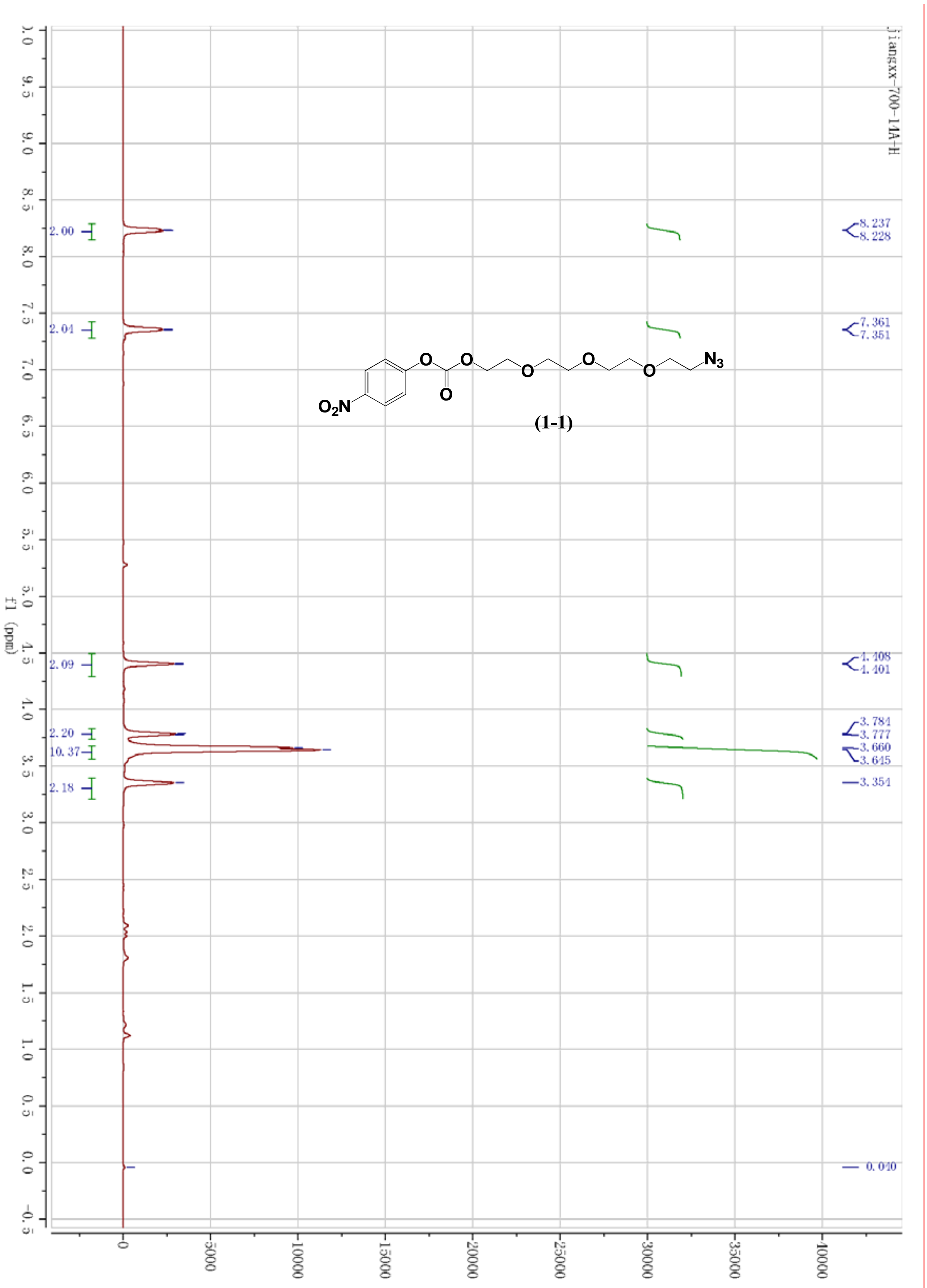
jiangxx-700-14A-H
(1-1)
8.237
8.228
7.361
7.351
4.408
4.401
3.784
3.777
3.660
3.645
3.354
0.040
2.00
2.04
2.09
2.20
10.37
2.18
f1 (ppm)

#

#

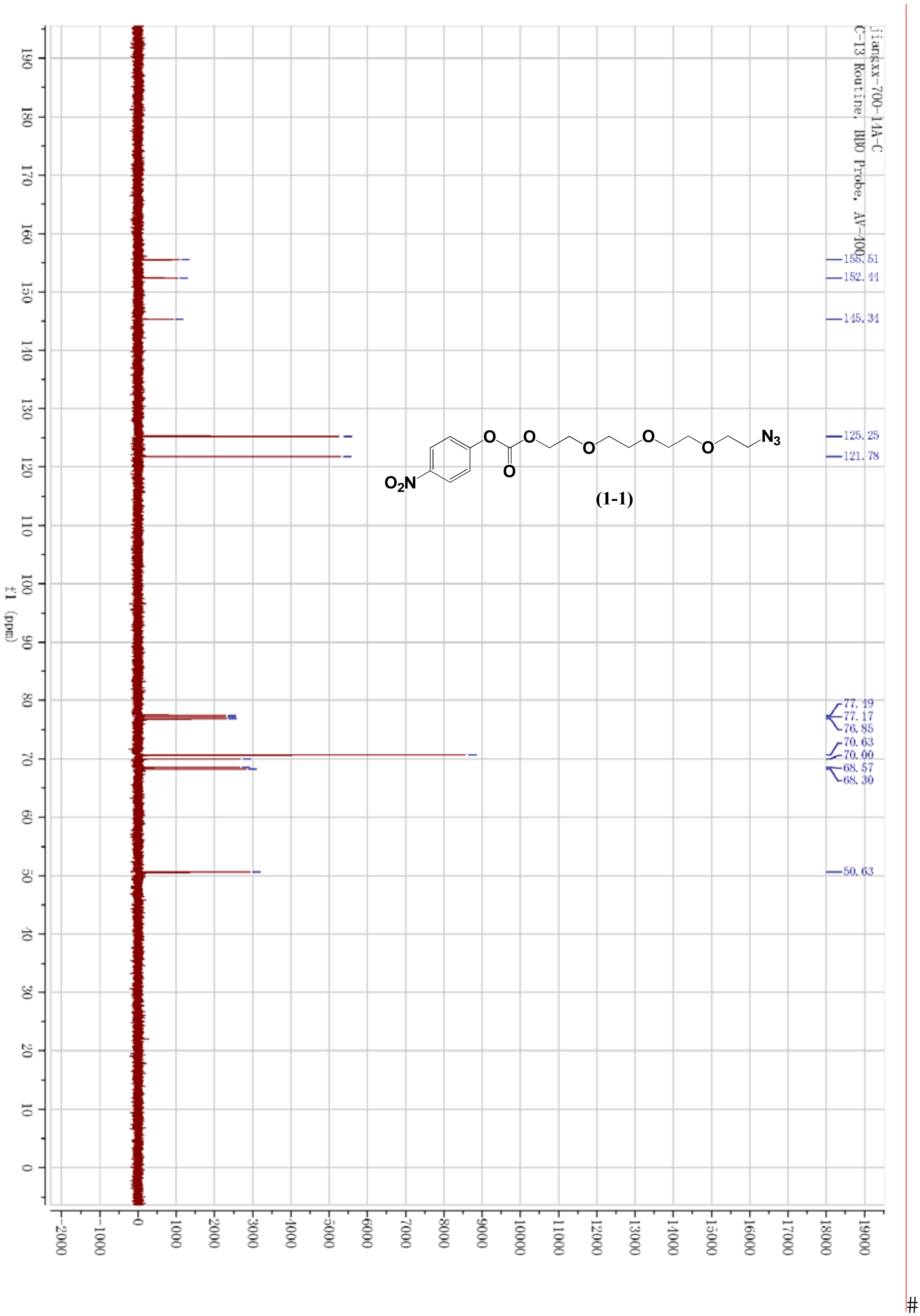
jiangxx-700-14A-C
C-13 Routine, BBO Probe, AV-400
155.51
152.44
145.34
125.25
121.78
77.49
77.17
76.85
70.63
70.00
68.57
68.30
50.63
O₂N
N₃
(1-1)
f1 (ppm)

#

#

#

#

#

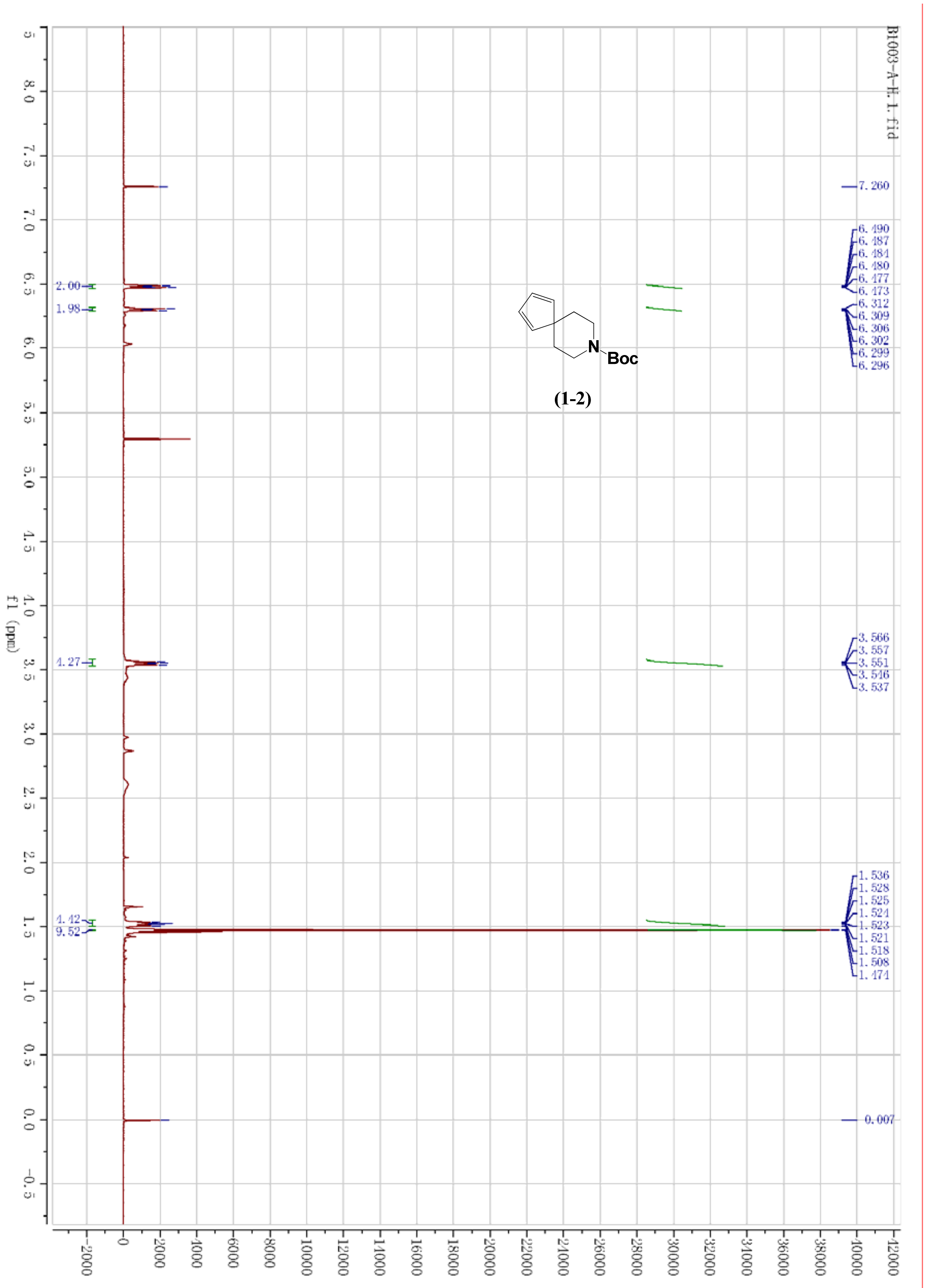
B1003-A-H.1.fid
7.260
6.490
6.487
6.484
6.480
6.477
6.473
6.312
6.309
6.306
6.302
6.299
6.296
3.566
3.557
3.551
3.546
3.537
1.536
1.528
1.525
1.524
1.523
1.521
1.518
1.508
1.474
0.007
2.00
1.98
4.27
4.42
9.52
N
Boc
(1-2)
f1 (ppm)

#

#

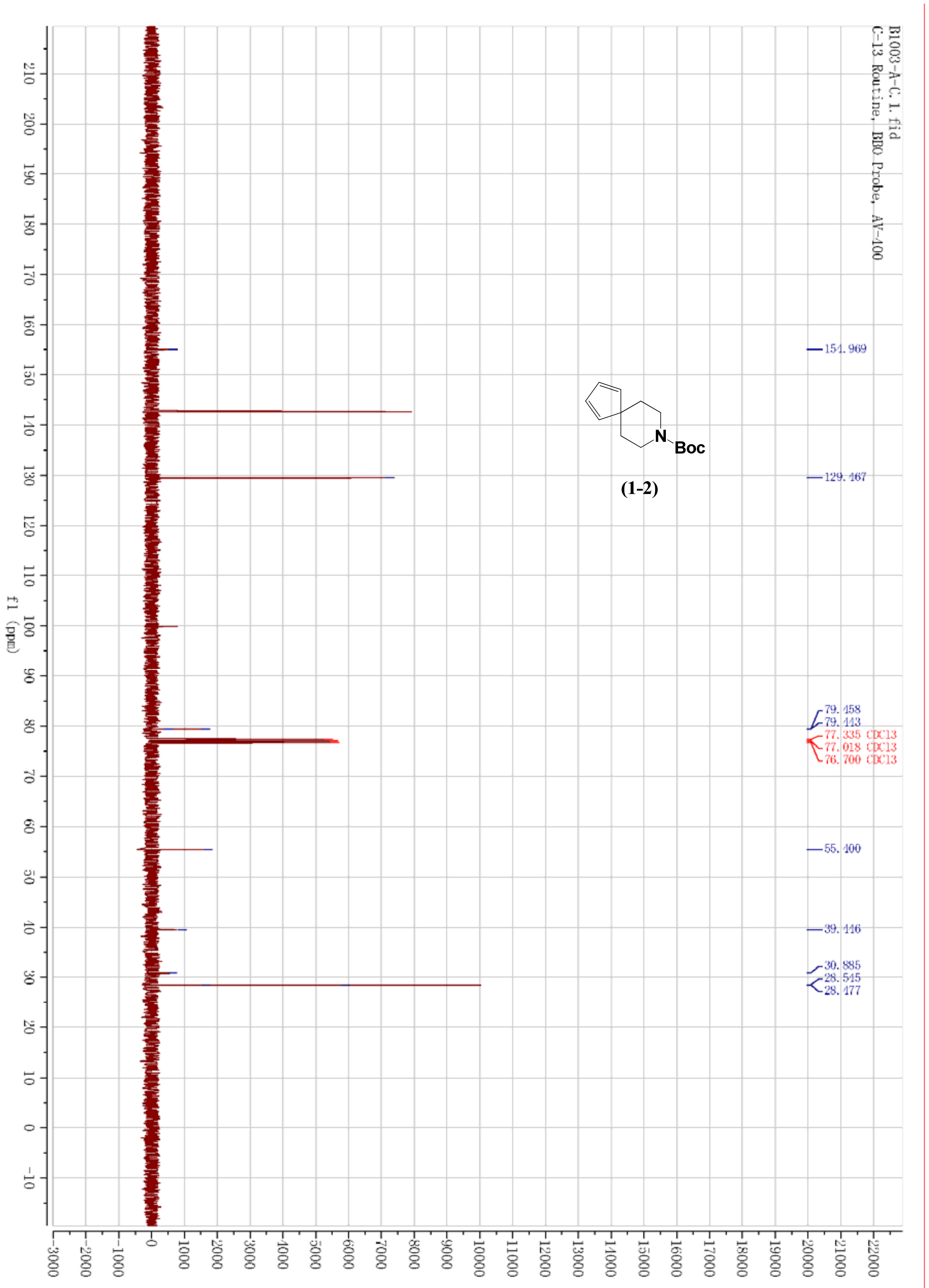
BI003-A-C.1.fid
C-13 Routine, BBO Probe, AV-400
154.969
129.467
79.458
79.443
77.335 CDCl3
77.018 CDCl3
76.700 CDCl3
55.400
39.446
30.885
28.545
28.477
N
Boc
(1-2)
f1 (ppm)

#

#

#

#

#

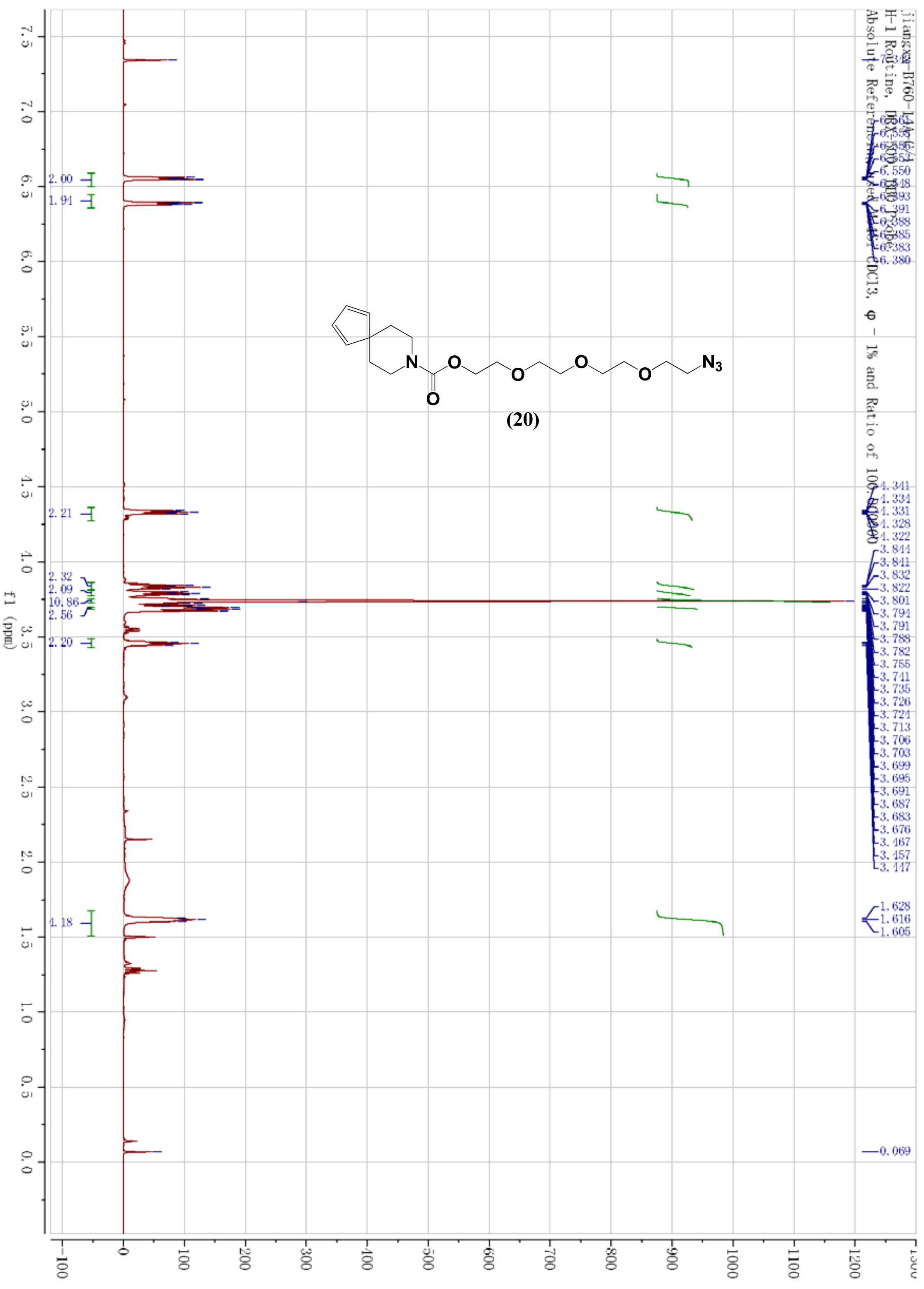

#

#

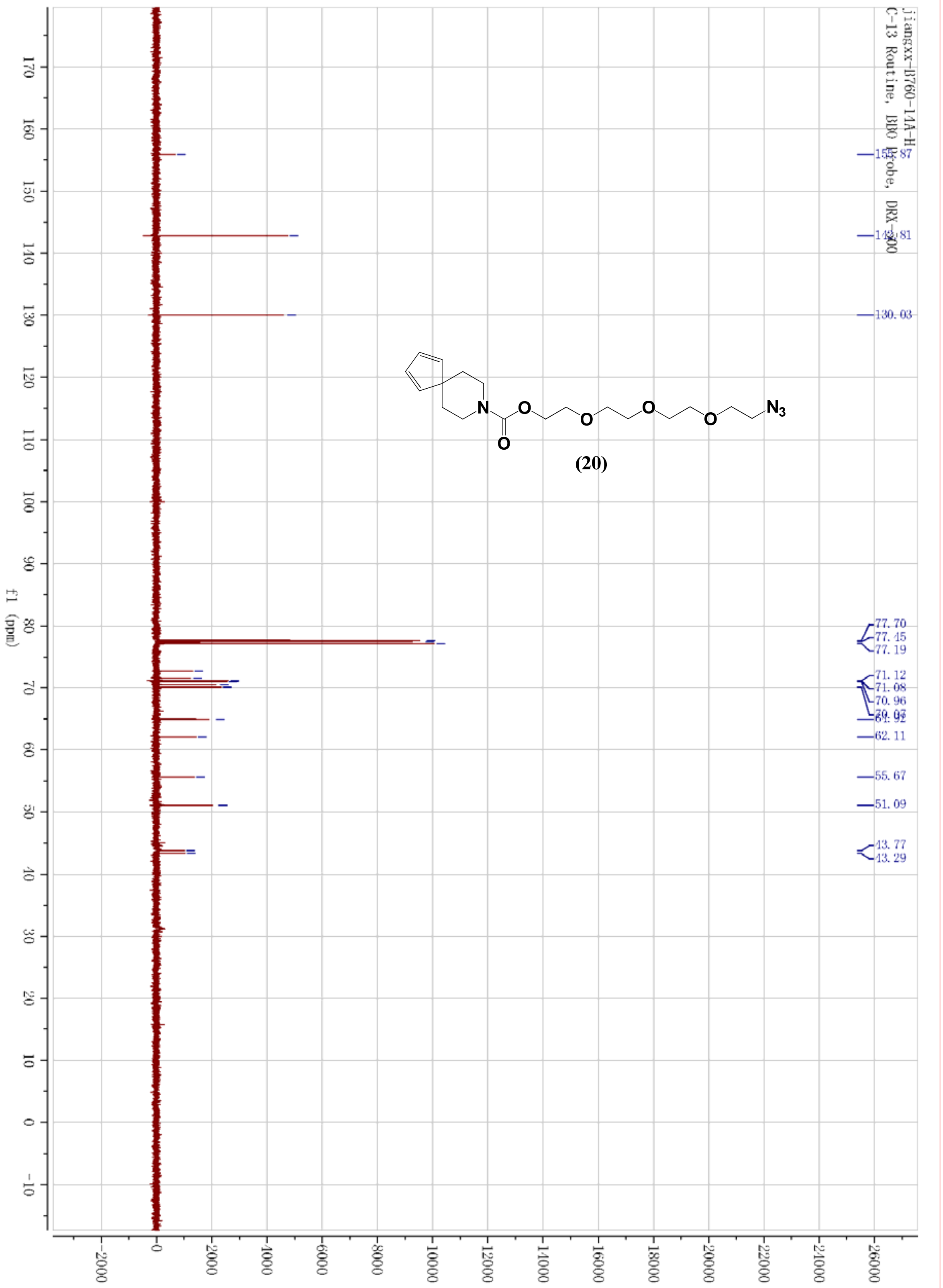
jiangxx-B760-14A-H
C-13 Routine, BBO probe, DRX-400
130.03
77.70
77.45
77.19
71.12
71.08
70.96
62.11
55.67
51.09
43.77
43.29
f1 (ppm)
N
O
N3
(20)

#

#

#

#

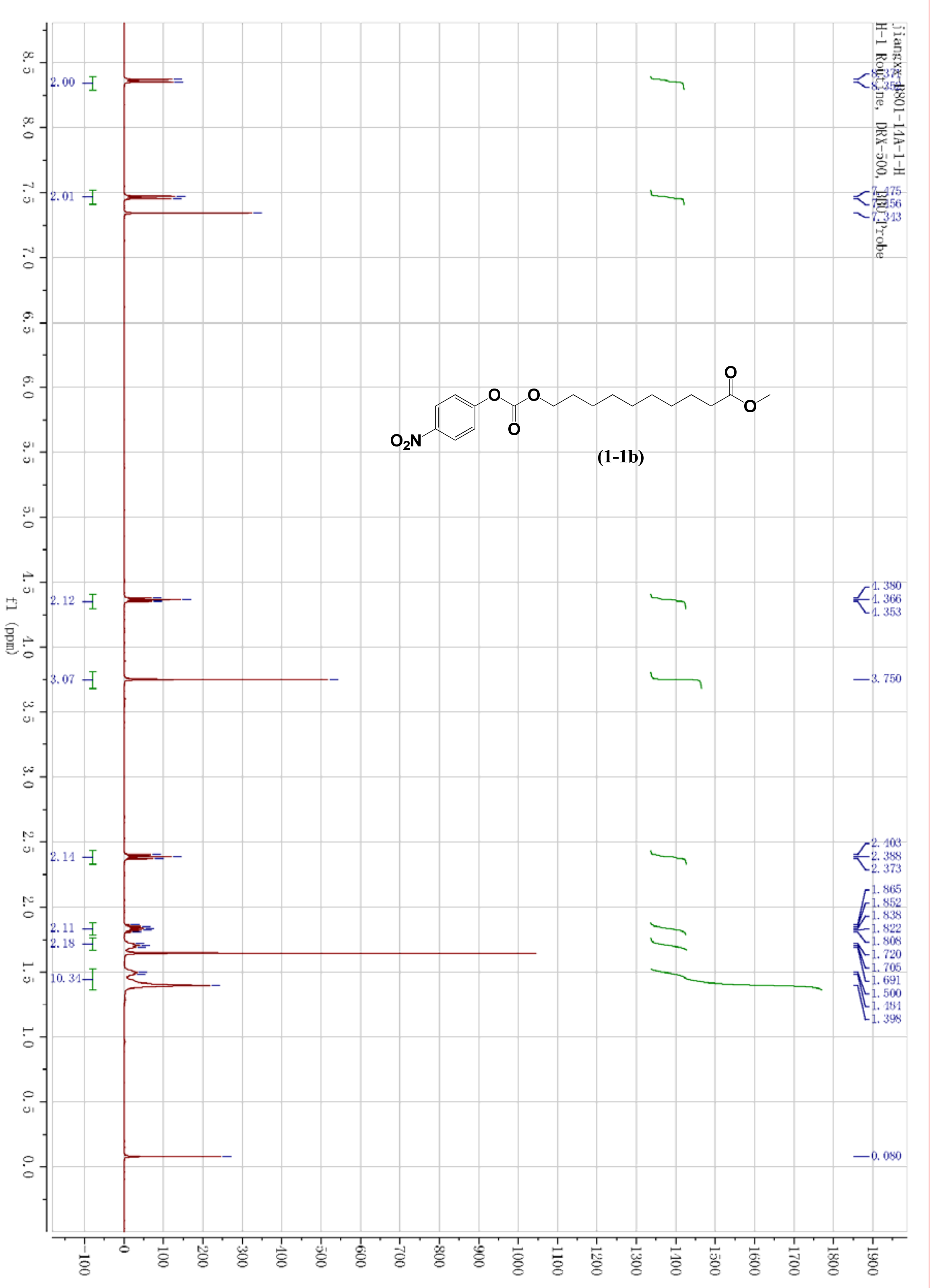
jiangxw-B801-14A-1-H
H-1 Routine, DRX-300, BBO Probe
(1-1b)
f1 (ppm)

#

#

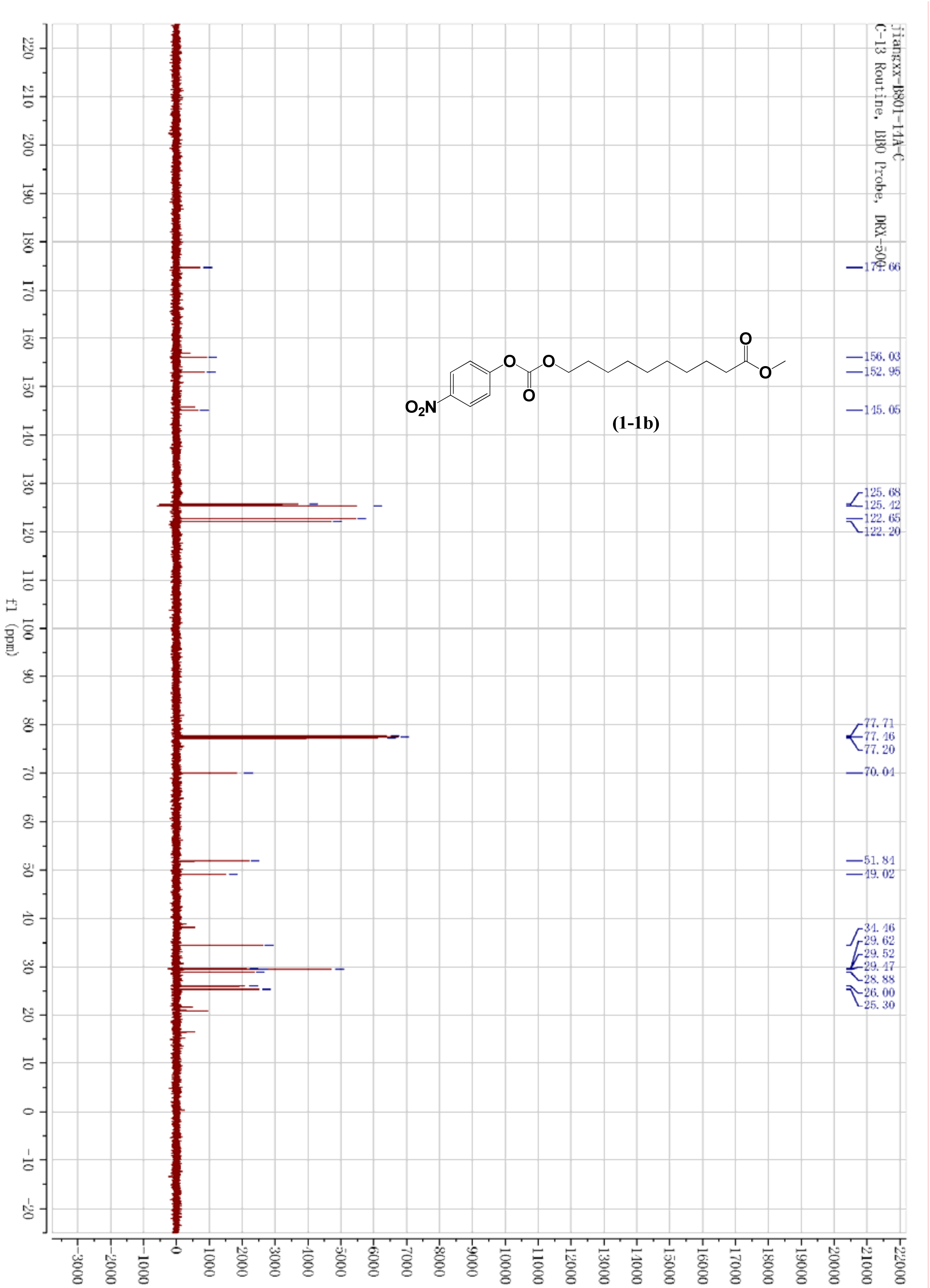
jiangxx-B801-14A-C
C-13 Routine, BBO Probe, DRX-500
174.66
156.03
152.95
145.05
125.68
125.42
122.65
122.20
77.71
77.46
77.20
70.04
51.84
49.02
34.46
29.62
29.52
29.47
28.88
26.00
25.30
O_2N
(1-1b)
f1 (ppm)

#

#

#

#

#

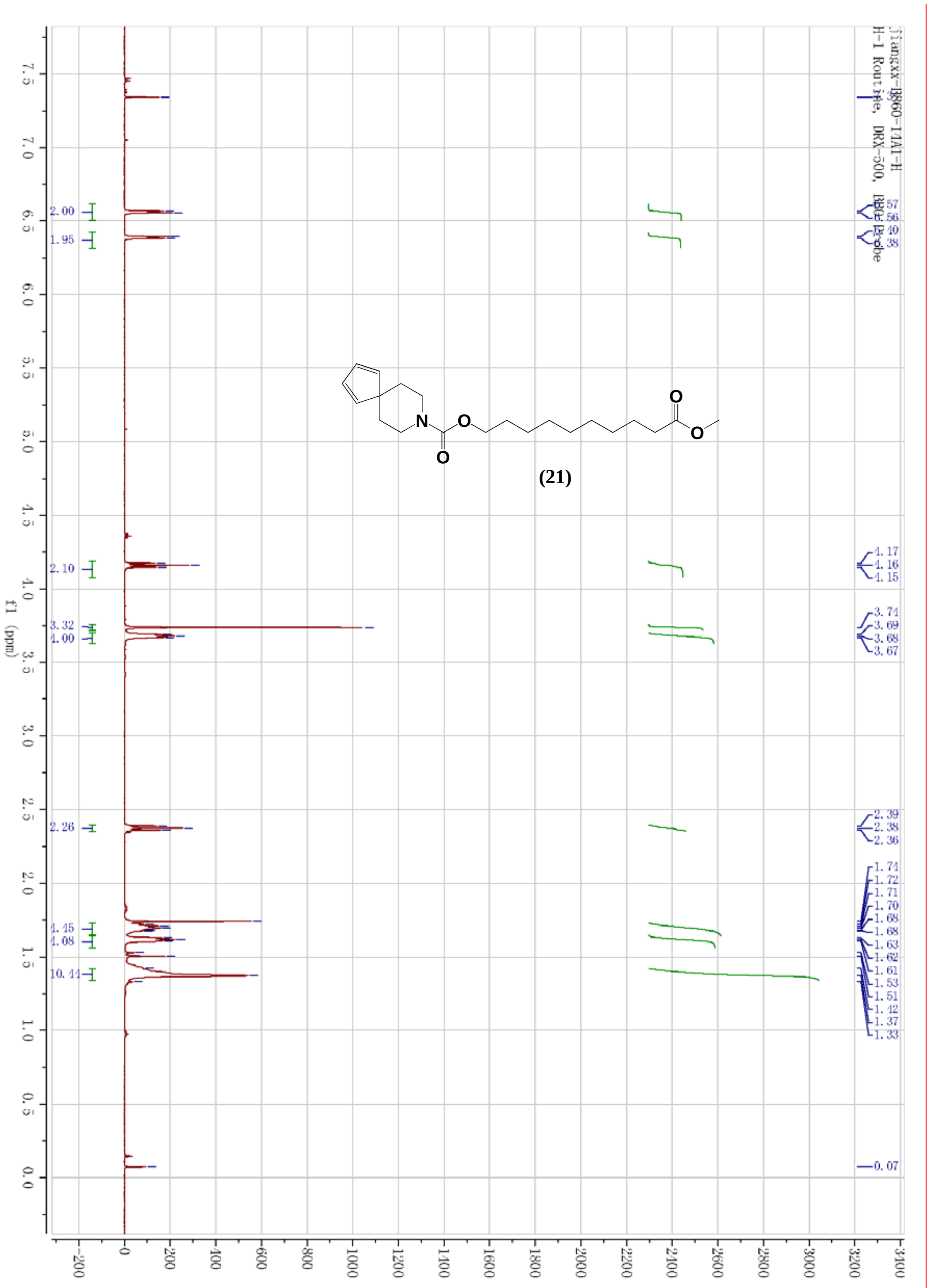

f1 (ppm)
(21)

#

#

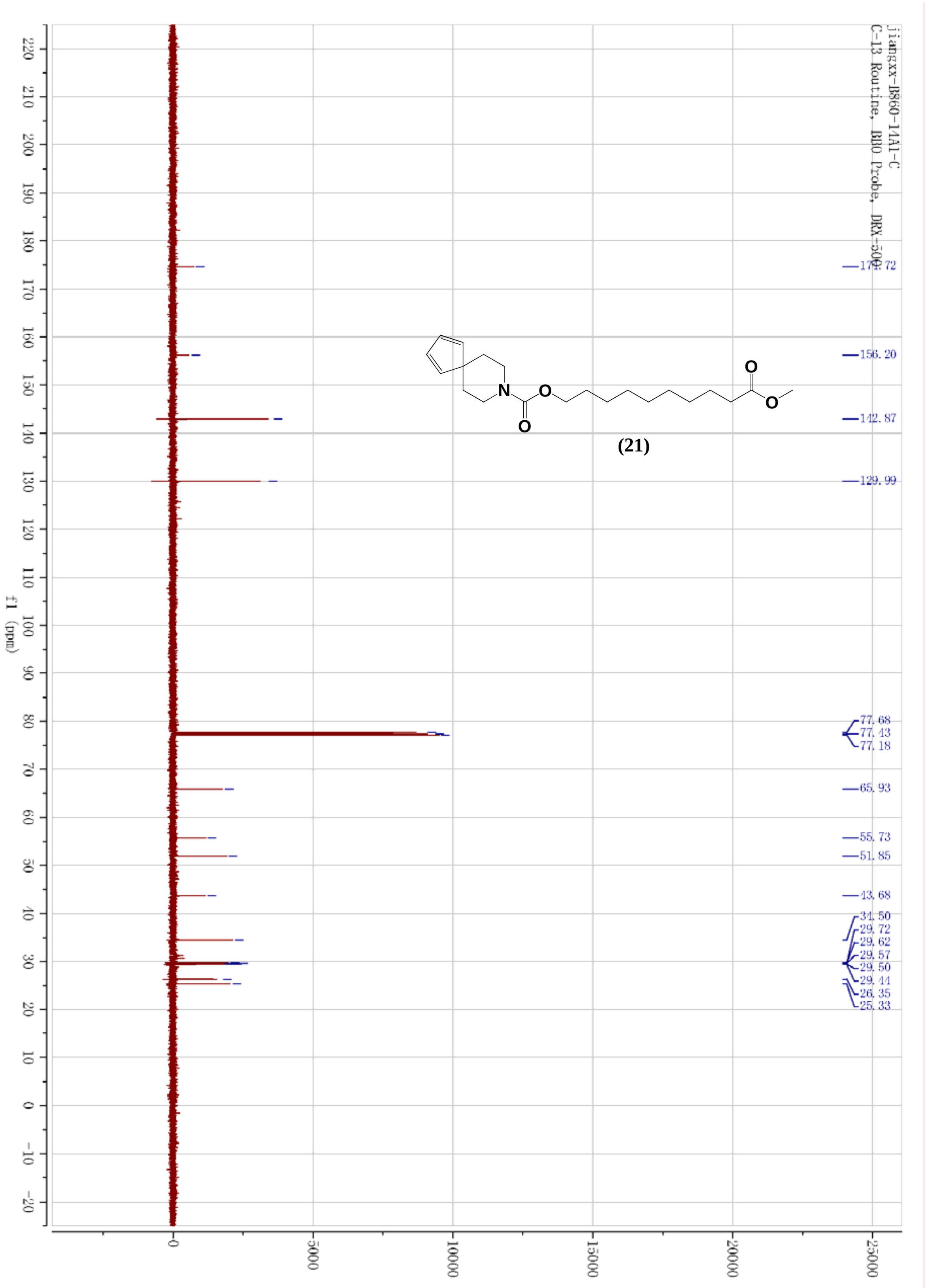

C-13 Routine, BBO Probe, DRX-500
156.20
142.87
129.99
77.68
77.43
77.18
65.93
55.73
51.85
43.68
34.50
29.72
29.62
29.57
29.50
29.44
26.35
25.33
f1 (ppm)
(21)

#

#

#

#

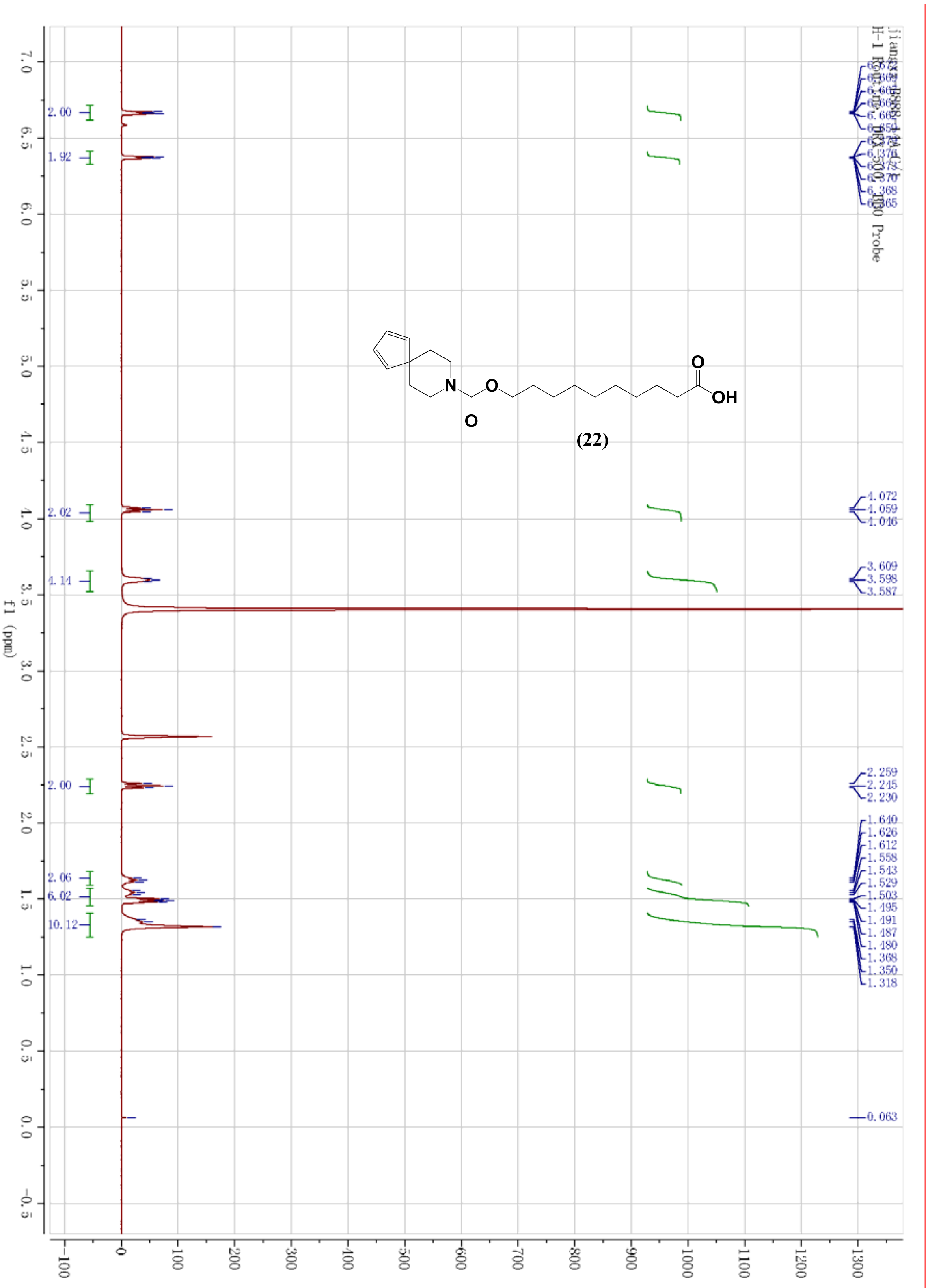

O
N
O
OH
O
(22)
f1 (ppm)

#

#

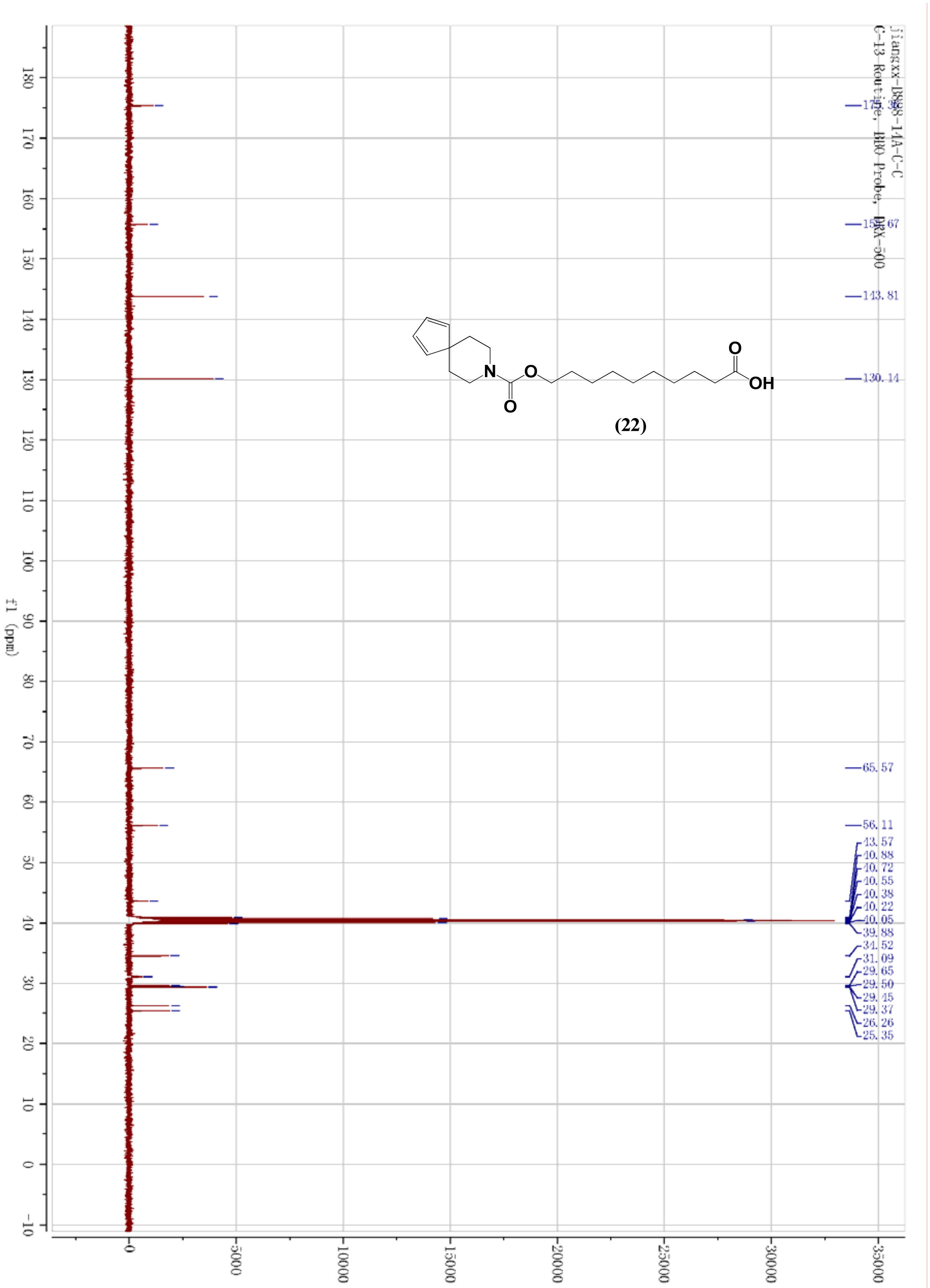
C-13 Routine, BBO Probe, DRX-500
143.81
130.14
65.57
56.11
43.57
40.88
40.72
40.55
40.38
40.22
40.05
39.88
34.52
31.09
29.65
29.50
29.45
29.37
26.26
25.35
f1 (ppm)
N
O
OH

(22)

#

#

#

#

#

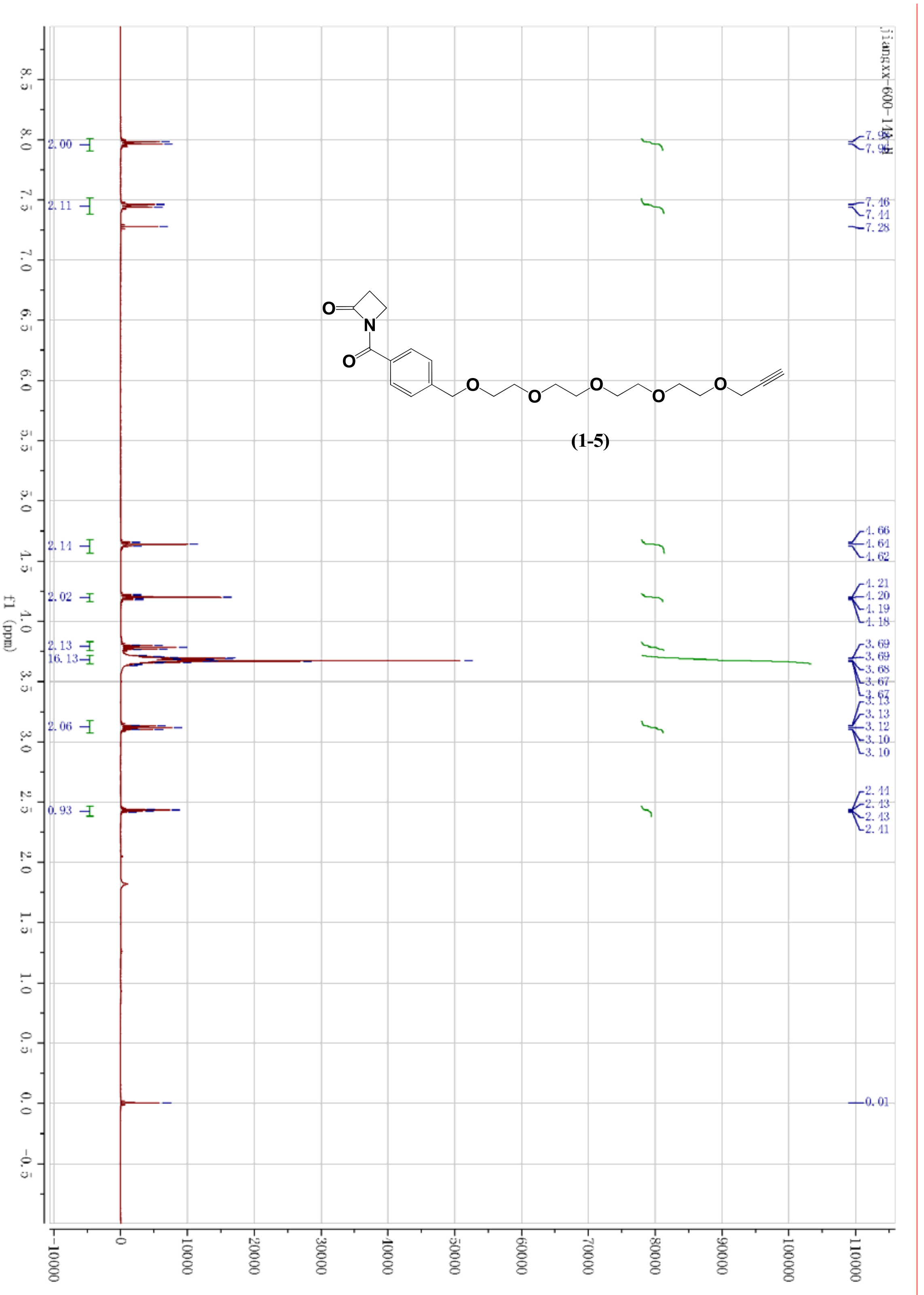
(1-5)

#

#

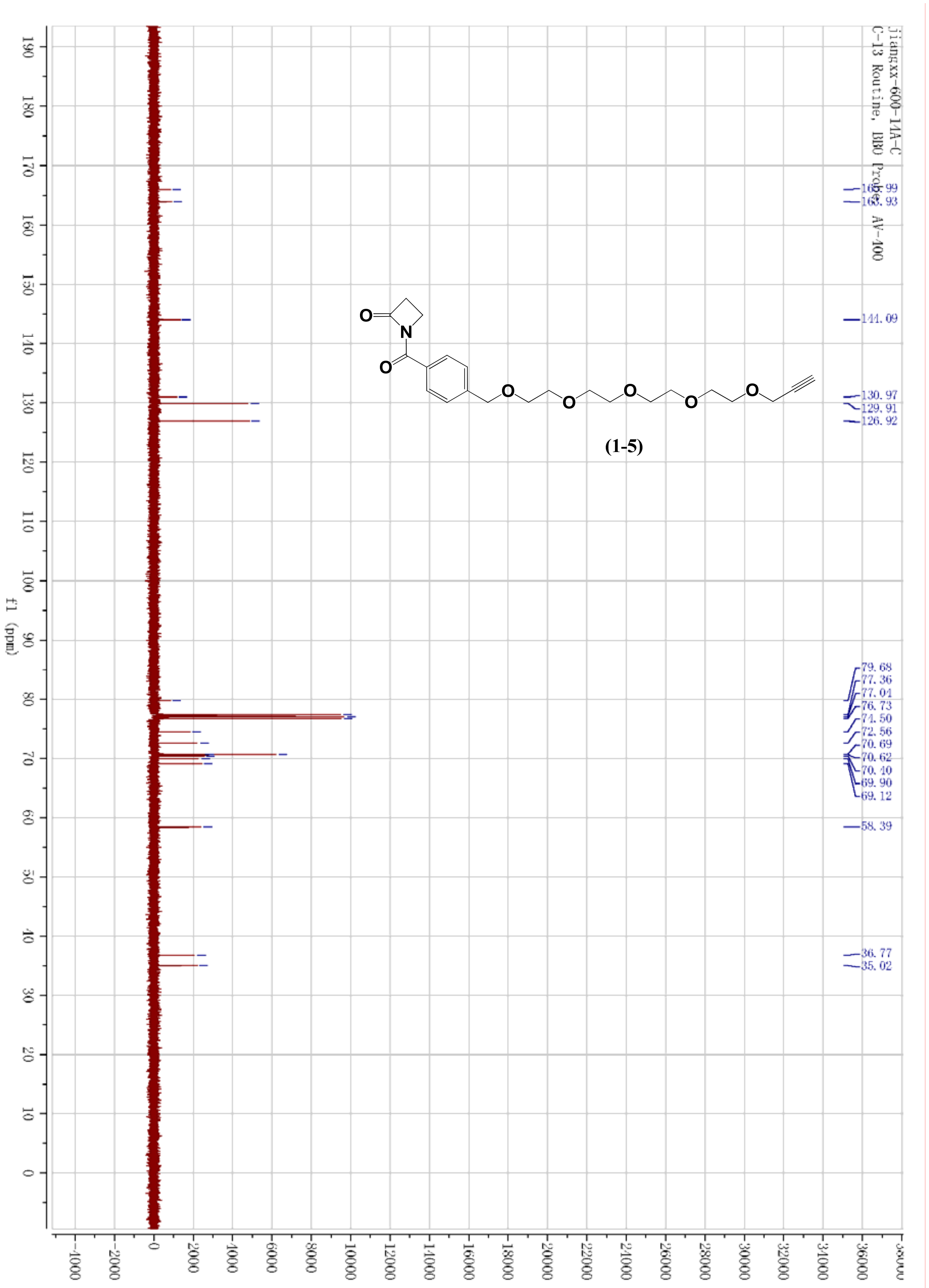
jiangxx-600-14A-C
C-13 Routine, BBO Probe, AV-400
141.09
130.97
129.91
126.92
79.68
77.36
77.04
76.73
74.50
72.56
70.69
70.62
70.10
69.90
69.12
58.39
36.77
35.02
(1-5)
f1 (ppm)

#

#

#

#

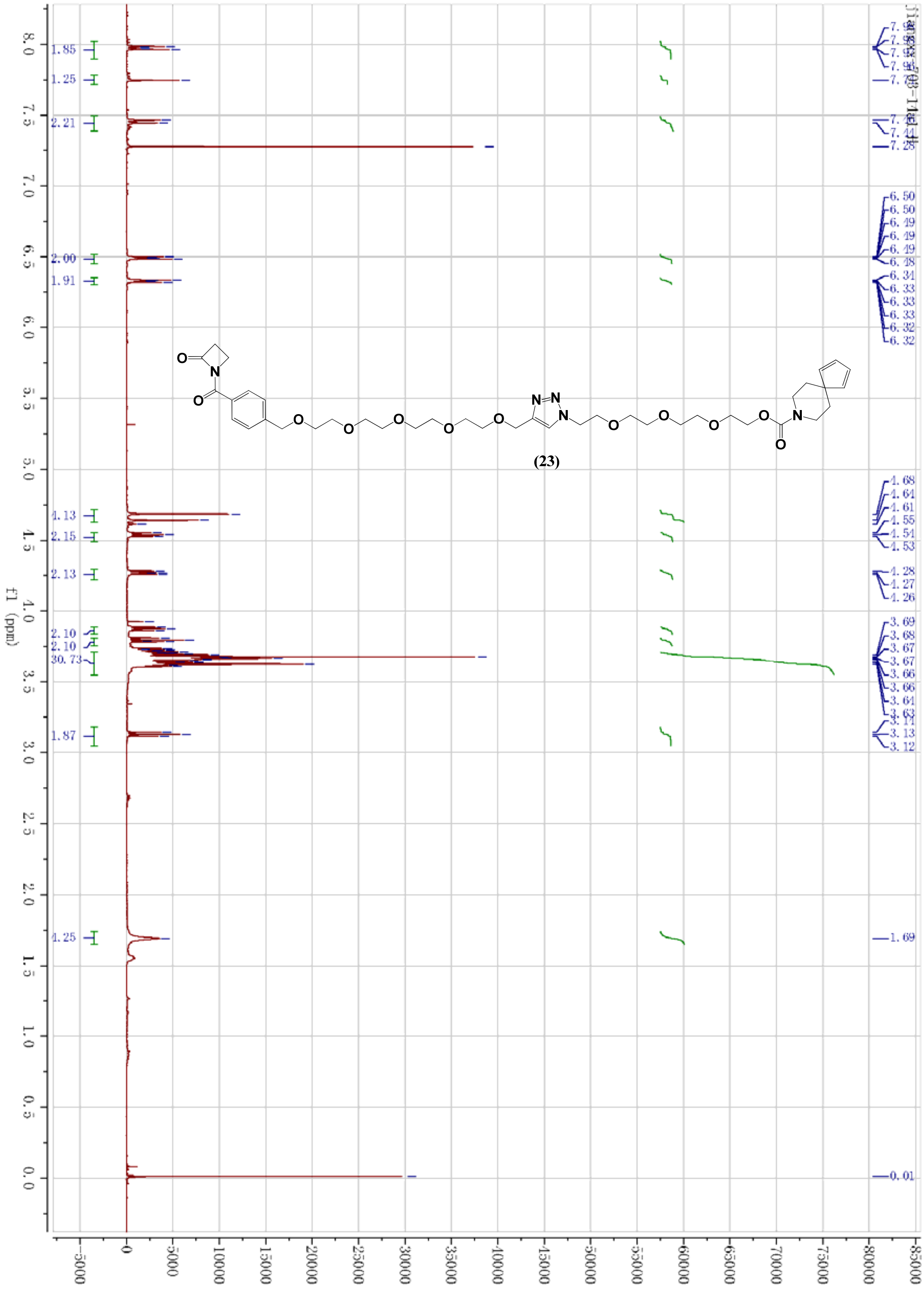
(23)

#

#

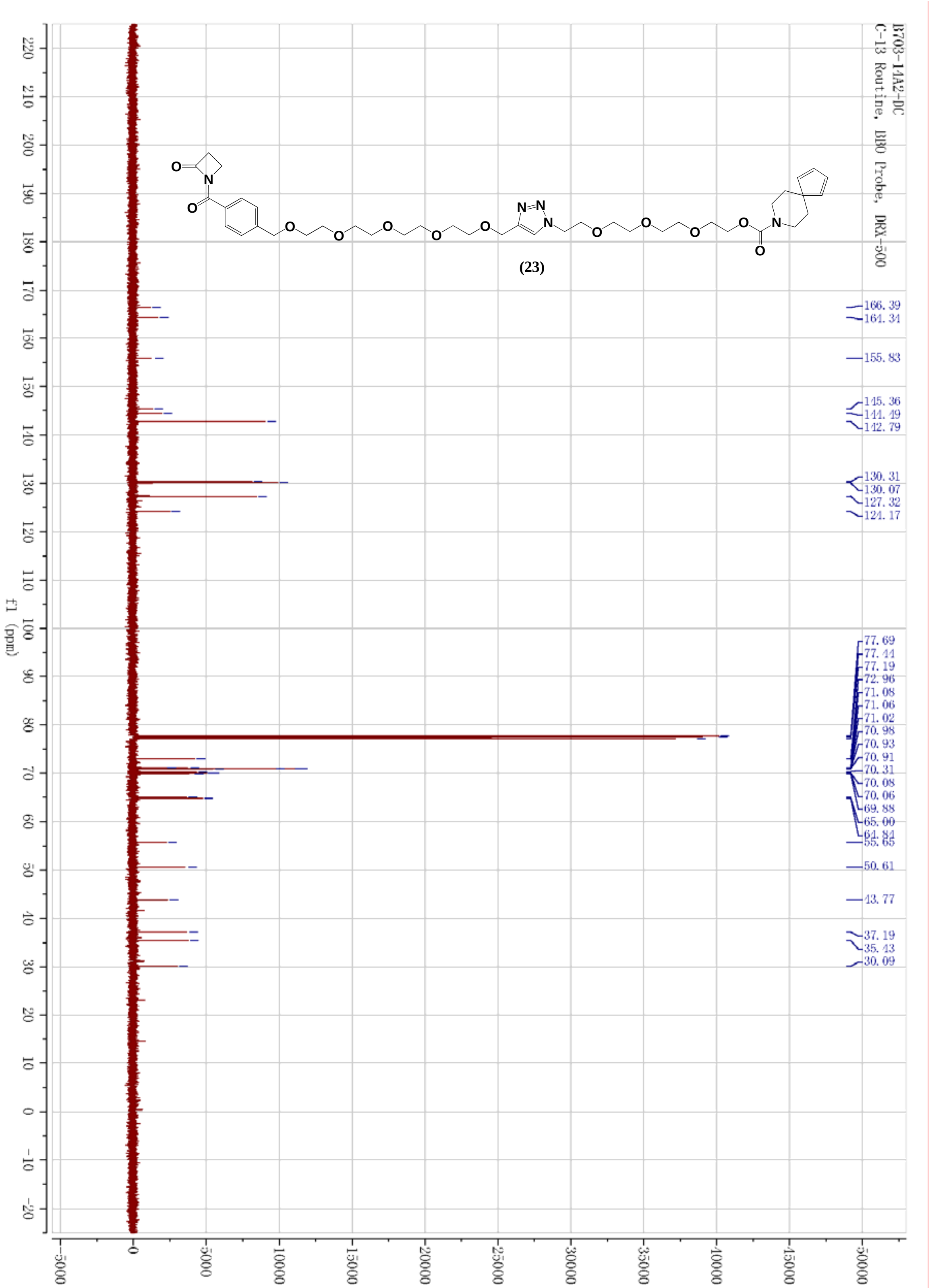
B703-14A2-DC
C-13 Routine, BBO Probe, DRX-500
(23)
166.39
164.34
155.83
145.36
144.49
142.79
130.31
130.07
127.32
124.17
77.69
77.44
77.19
72.96
71.08
71.06
71.02
70.98
70.93
70.91
70.31
70.08
70.06
69.88
65.00
64.84
55.65
50.61
43.77
37.19
35.43
30.09
f1 (ppm)

#

#

#

#

#

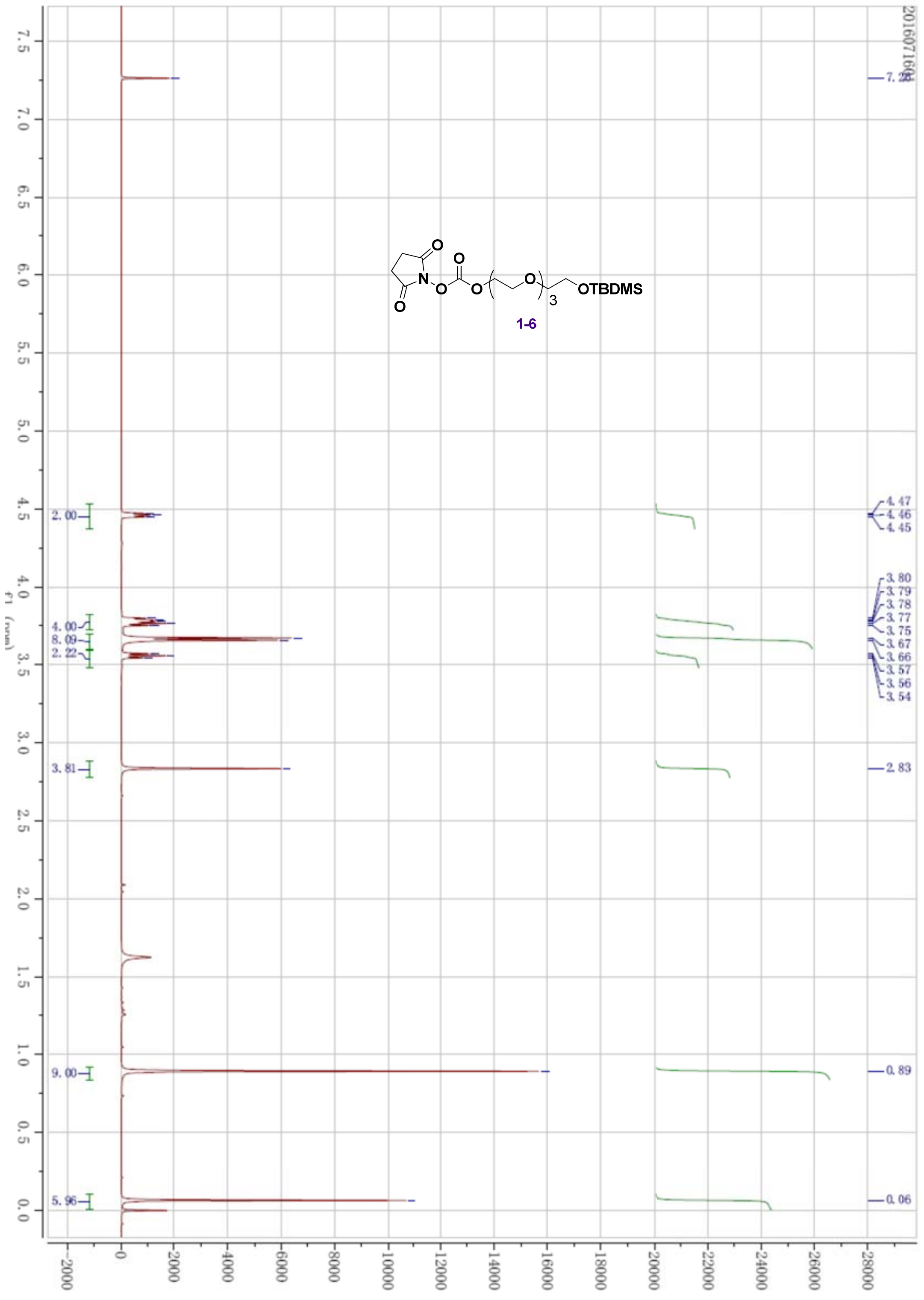
OTBDMS
1-6

#

#

#

#

#

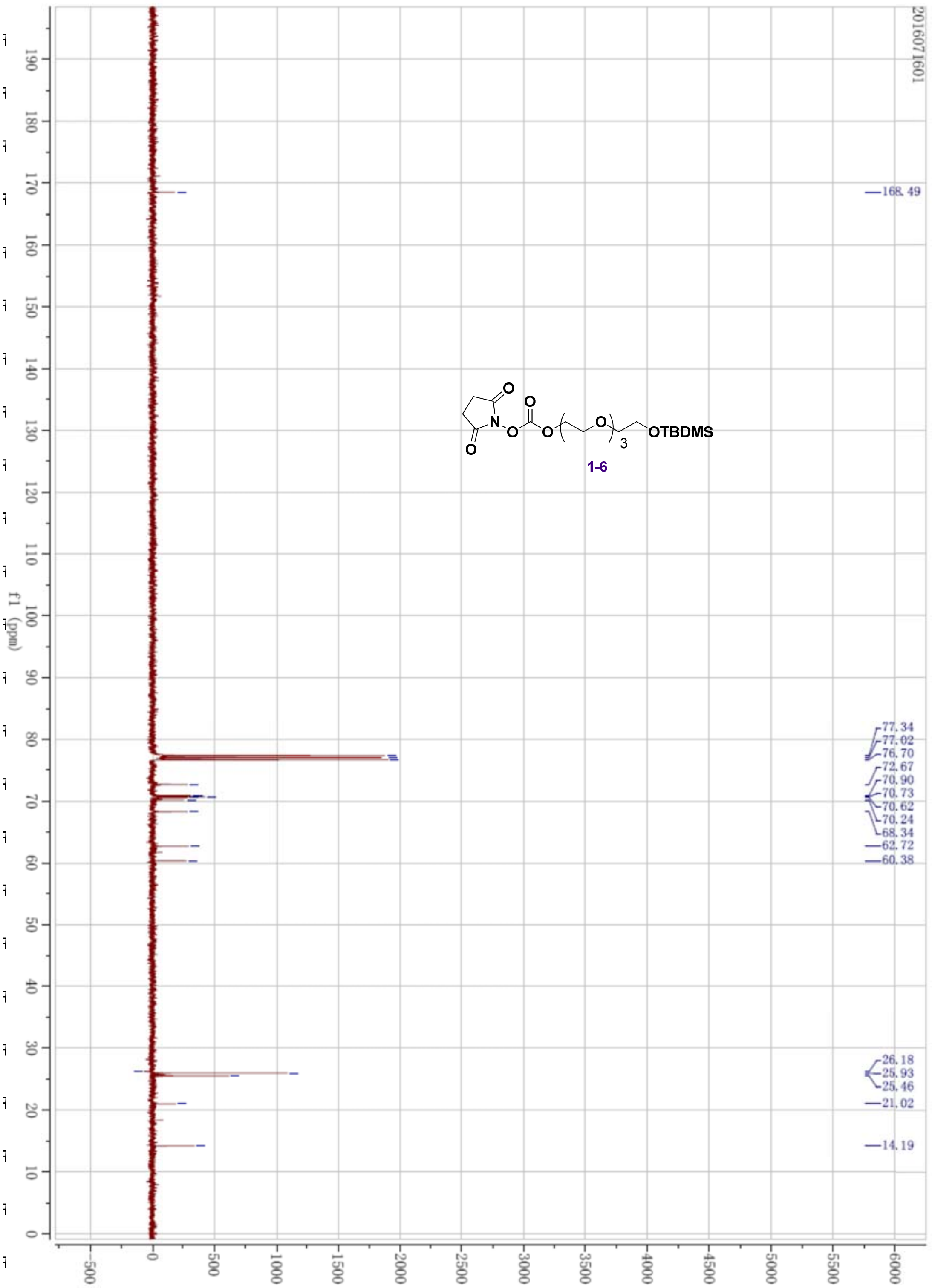

20160716O1
OTBDMS
3
1-6
168.49
77.34
77.02
76.70
72.67
70.90
70.73
70.62
70.24
68.34
62.72
60.38
26.18
25.93
25.46
21.02
14.19
f1 (ppm)

#

#

#

#

#

#

#

#

#

#

#

#

#

#

#

#

#

#

#

#

#

#

#

#

#

#

#

#

#

#

#

#

#

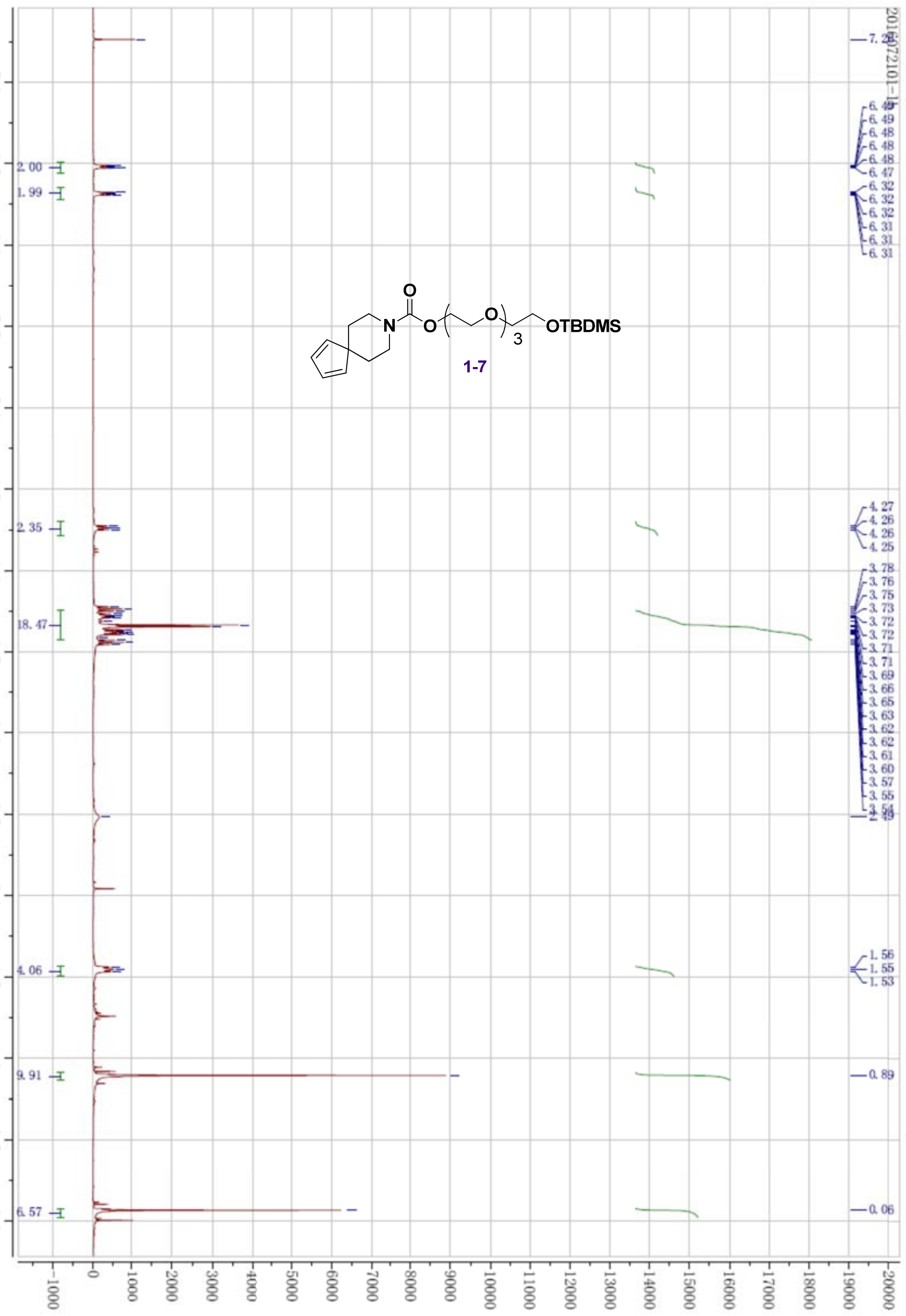

OTBDMS
1-7

#

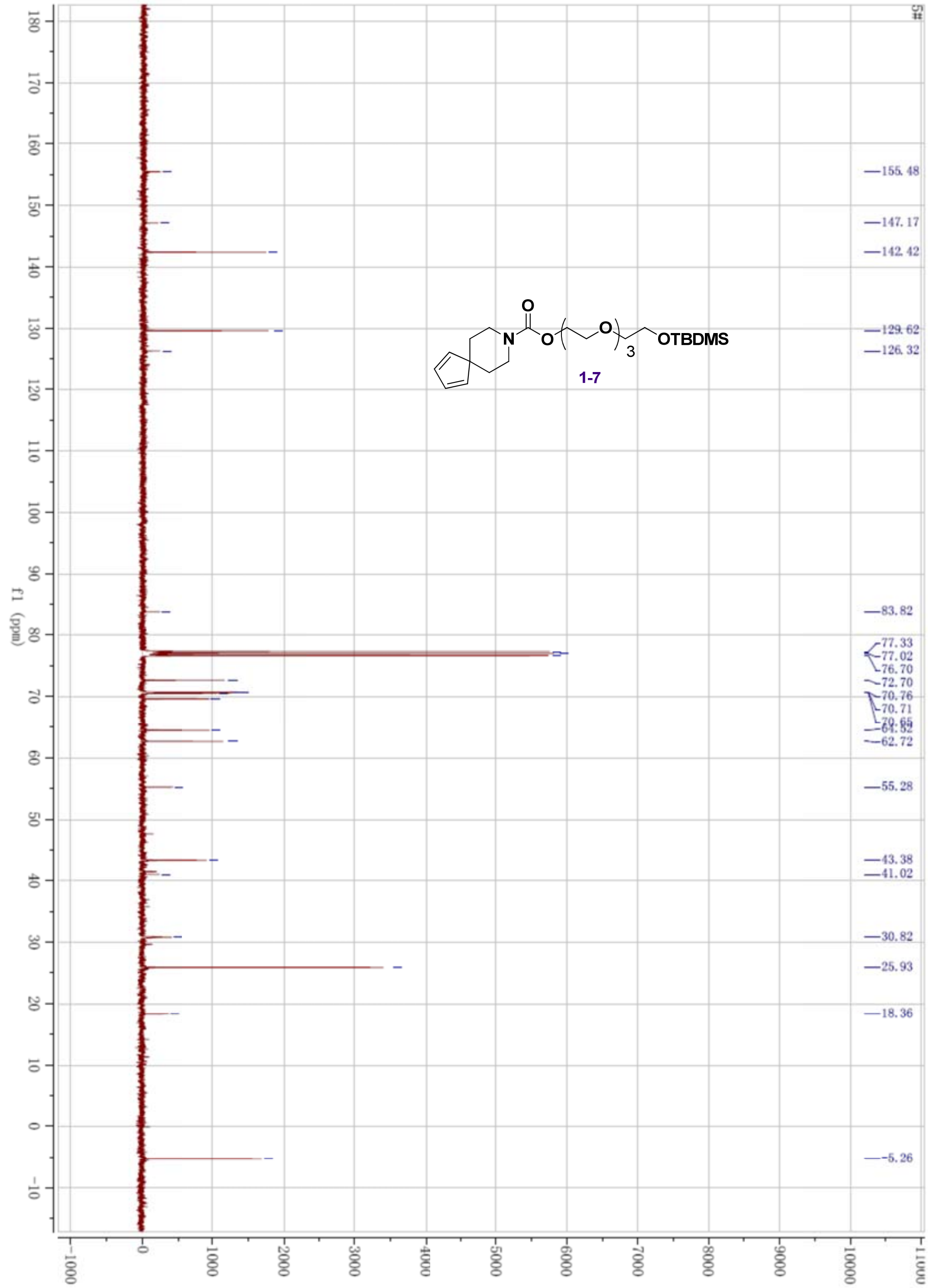

O
N
O
O
3
OTBDMS
1-7
155.48
147.17
142.42
129.62
126.32
83.82
77.33
77.02
76.70
72.70
70.76
70.71
70.65
64.52
62.72
55.28
43.38
41.02
30.82
25.93
18.36
-5.26
f1 (ppm)

#

#

#

#

#

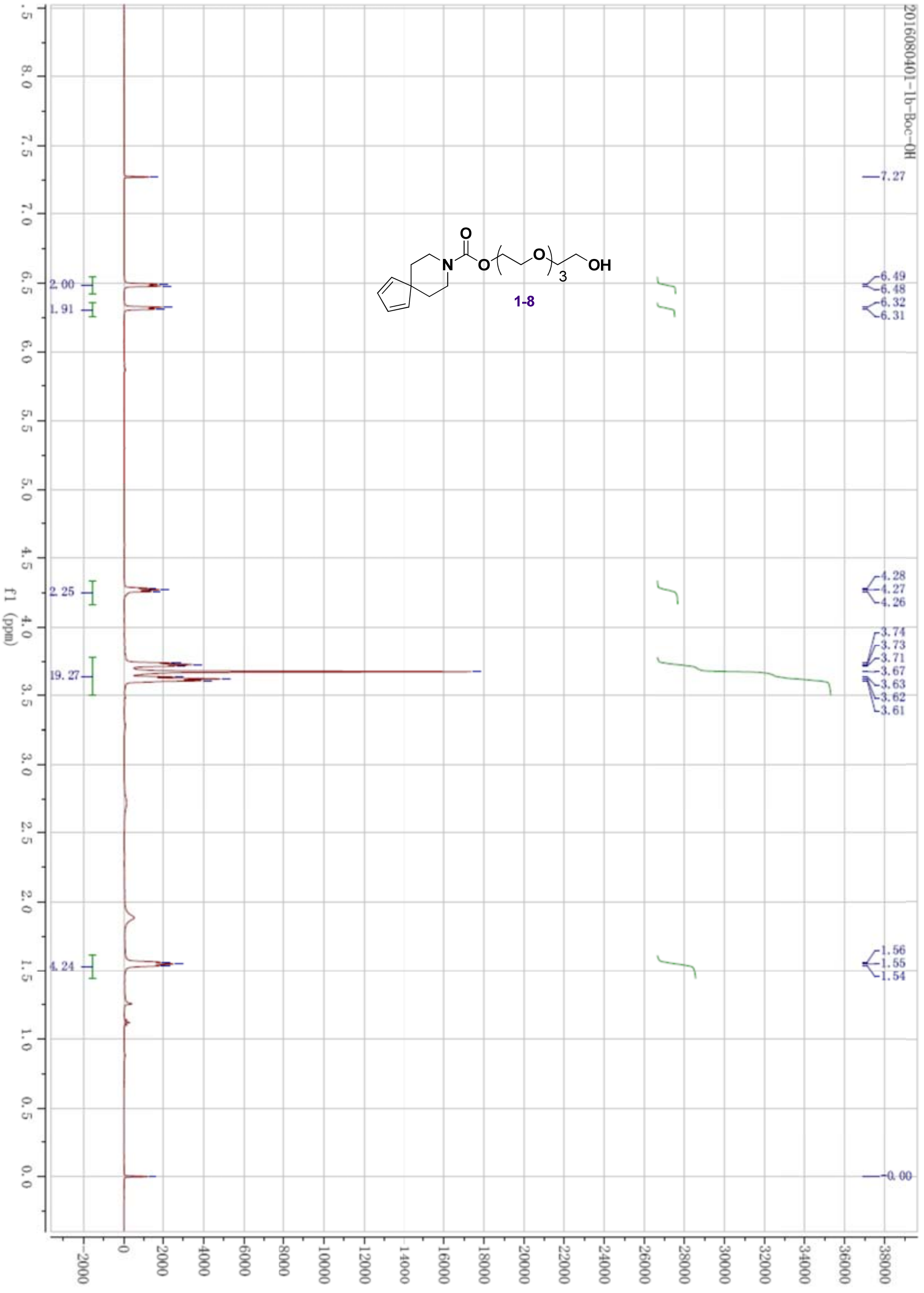

20160804O1-1b-Boc-OH
1-8
f1 (ppm)

#

#

#

#

#

#

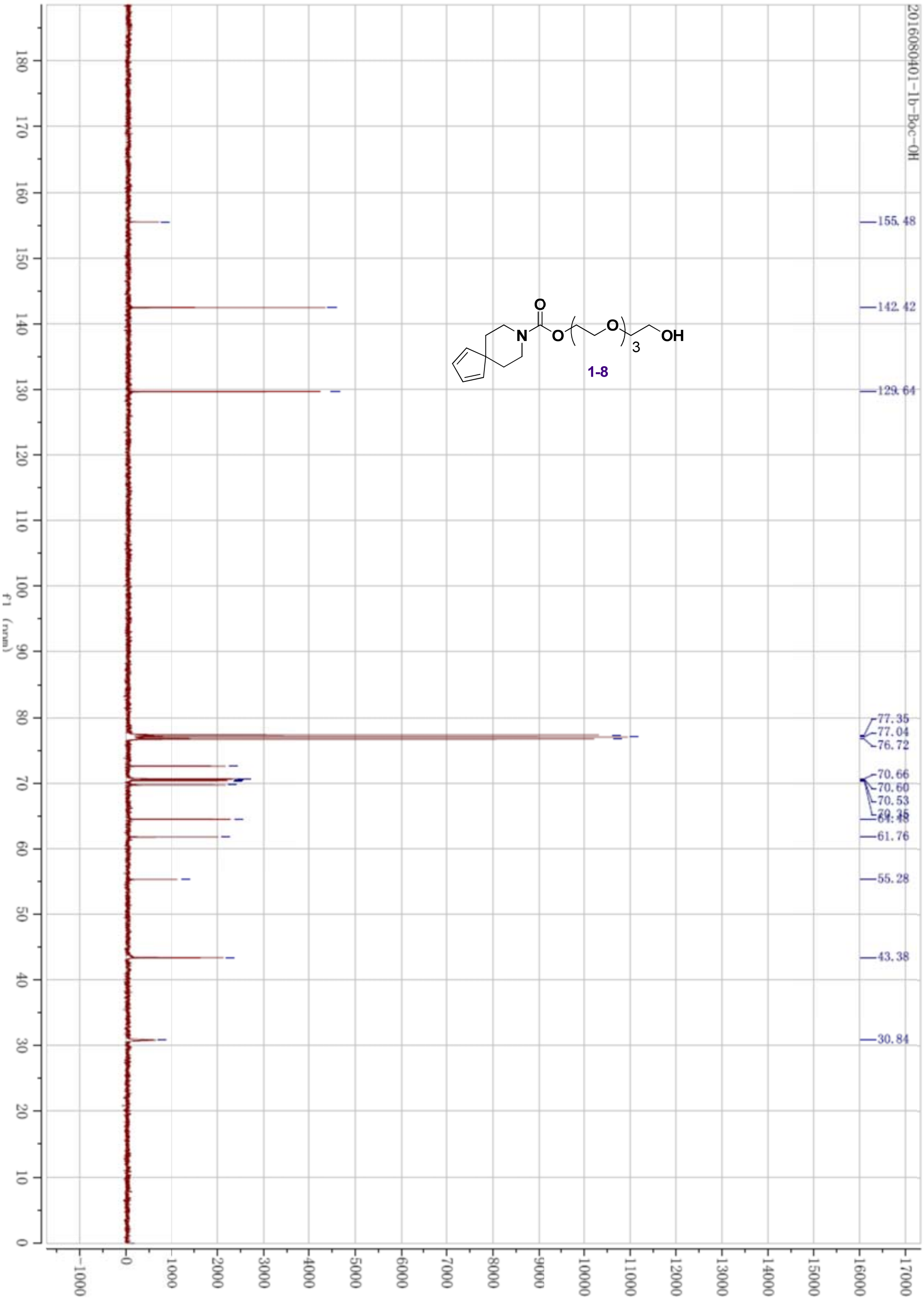
20160804 01-1b-Boc-OH
155.48
142.42
129.64
77.35
77.04
76.72
70.66
70.60
70.53
61.76
55.28
43.38
30.84
O
N
O
O
3
OH
1-8
f1 (ppm)

#

#

#

#

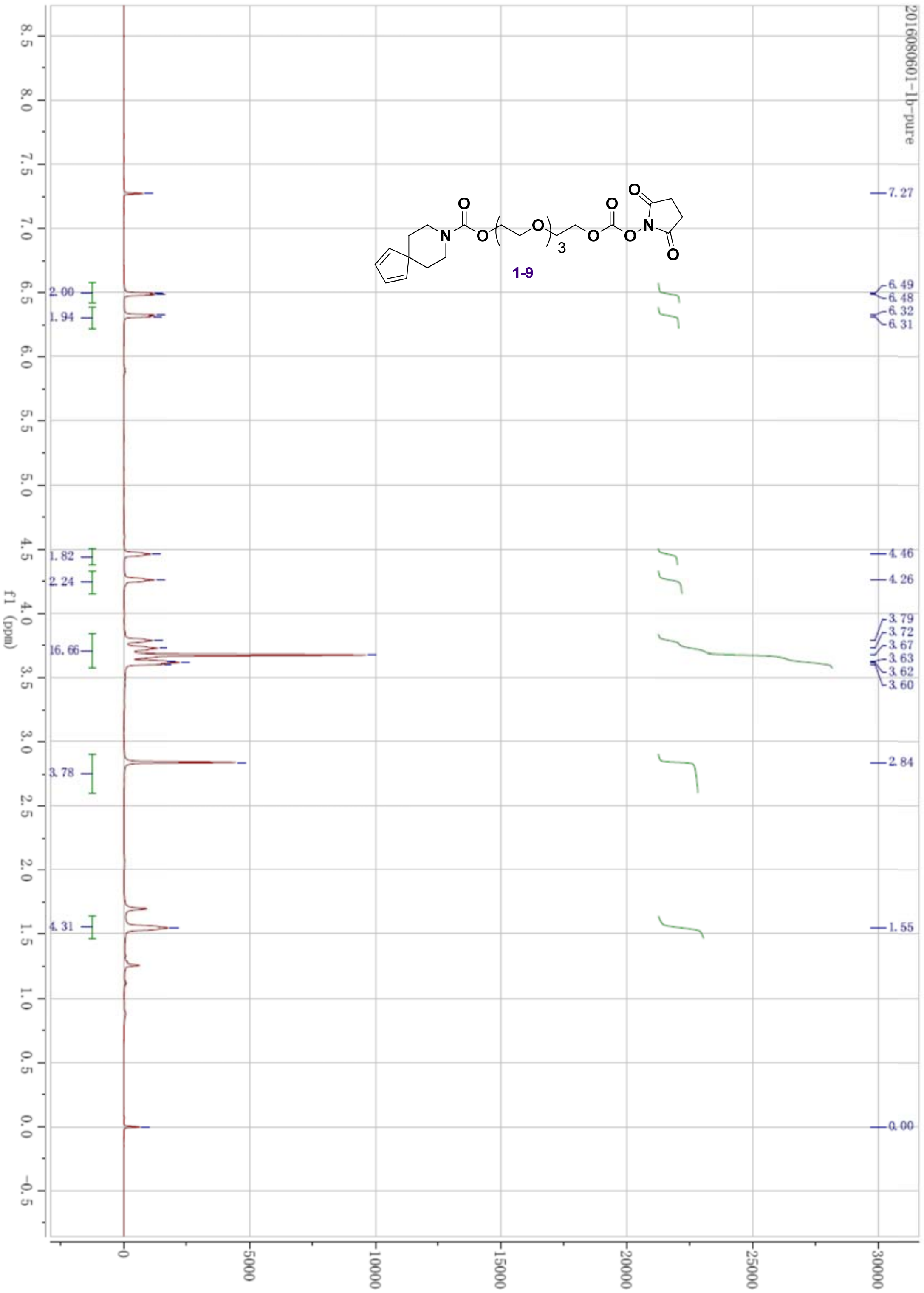

2016080601-1b-pure
1-9
f1 (ppm)
7.27
6.49
6.48
6.32
6.31
4.46
4.26
3.79
3.72
3.67
3.63
3.62
3.60
2.84
1.55
0.00
2.00
1.94
1.82
2.24
16.66
3.78
4.31

#

#

#

#

#

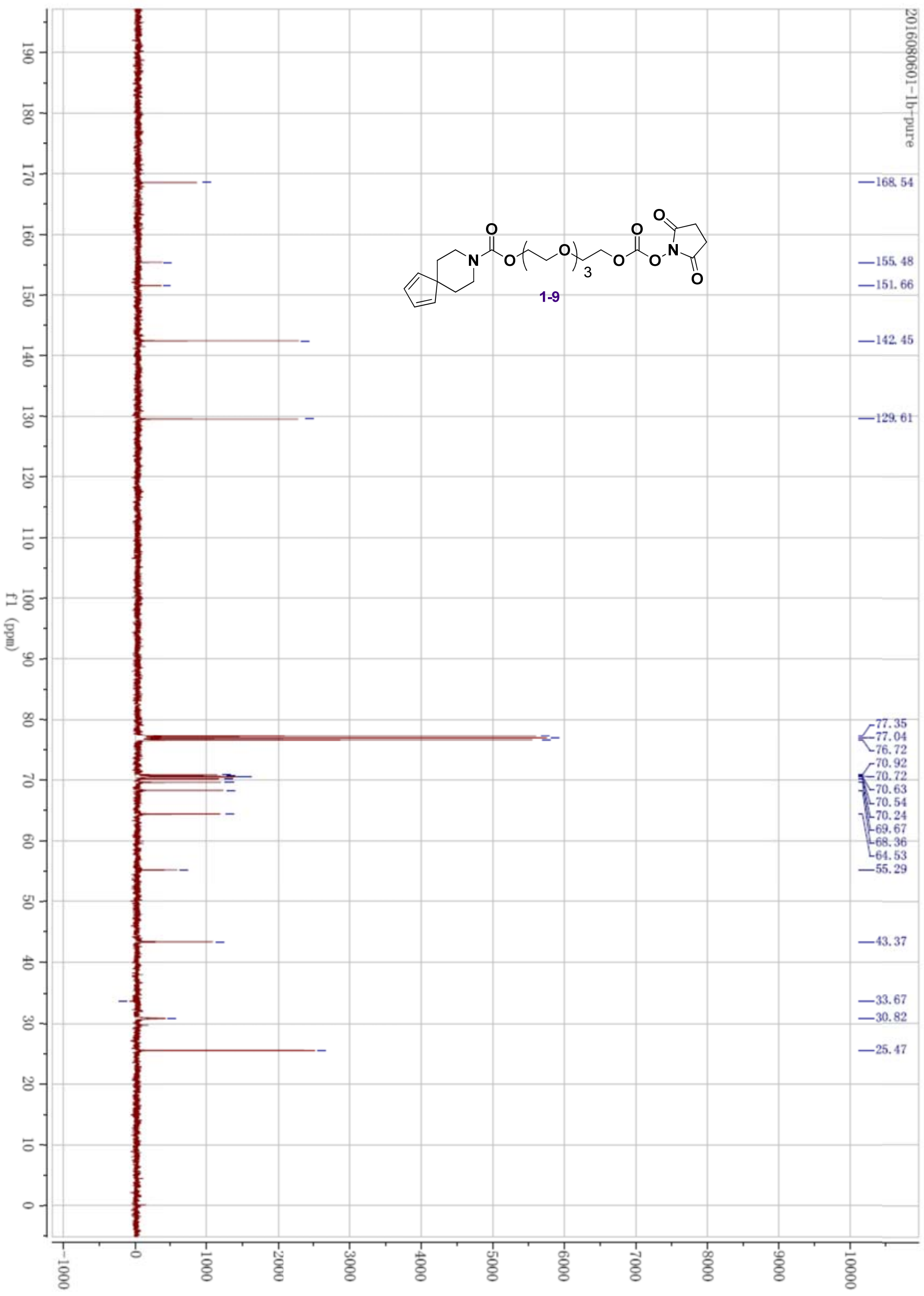
20160806O1-1b-pure
168.54
155.48
151.66
142.45
129.61
77.35
77.04
76.72
70.92
70.72
70.63
70.54
70.24
69.67
68.36
64.53
55.29
43.37
33.67
30.82
25.47
1-9
f1 (ppm)

#

#

#

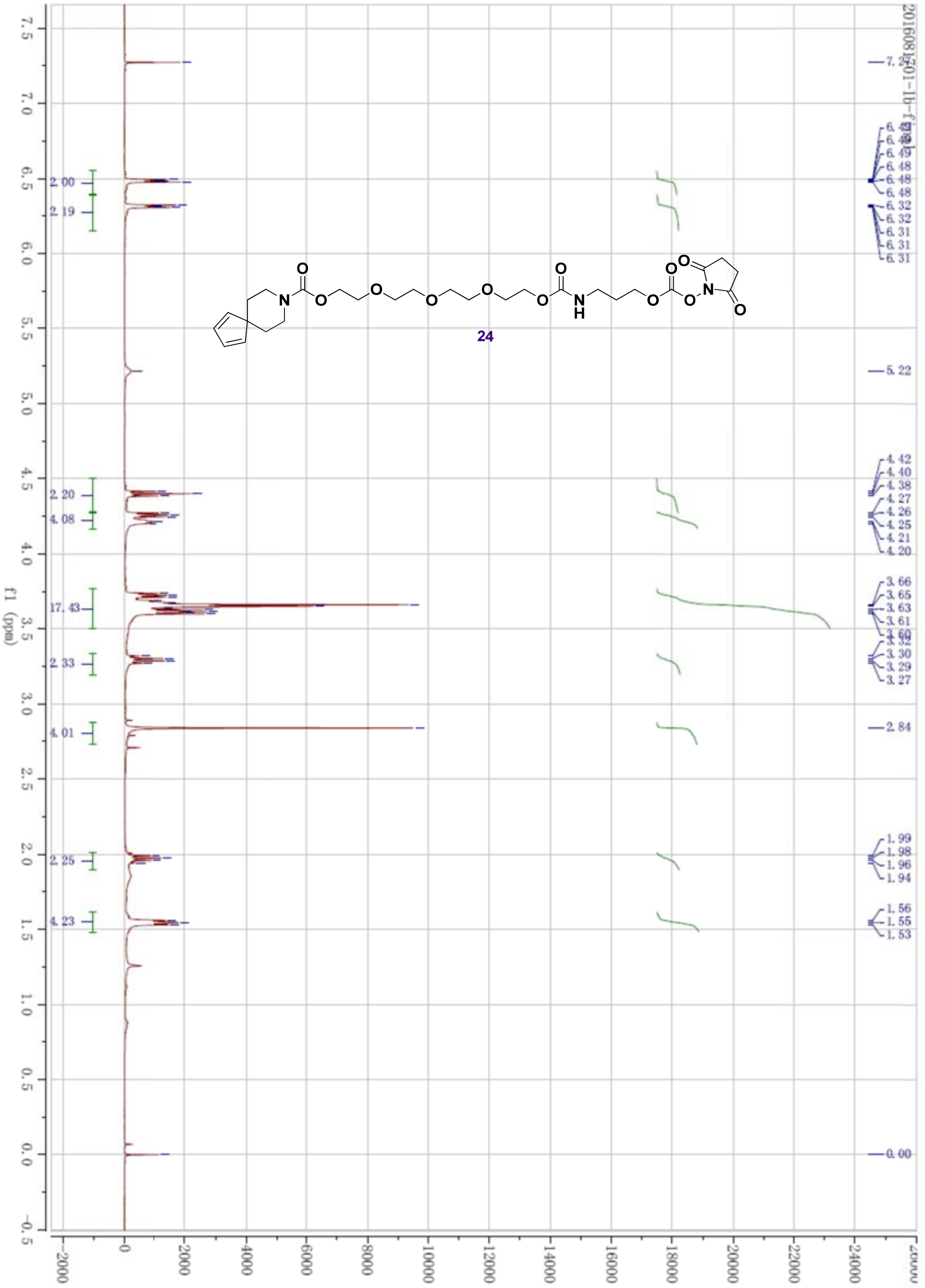

20160801-1b-final
24
f1 (ppm)

#

#

#

#

#

#

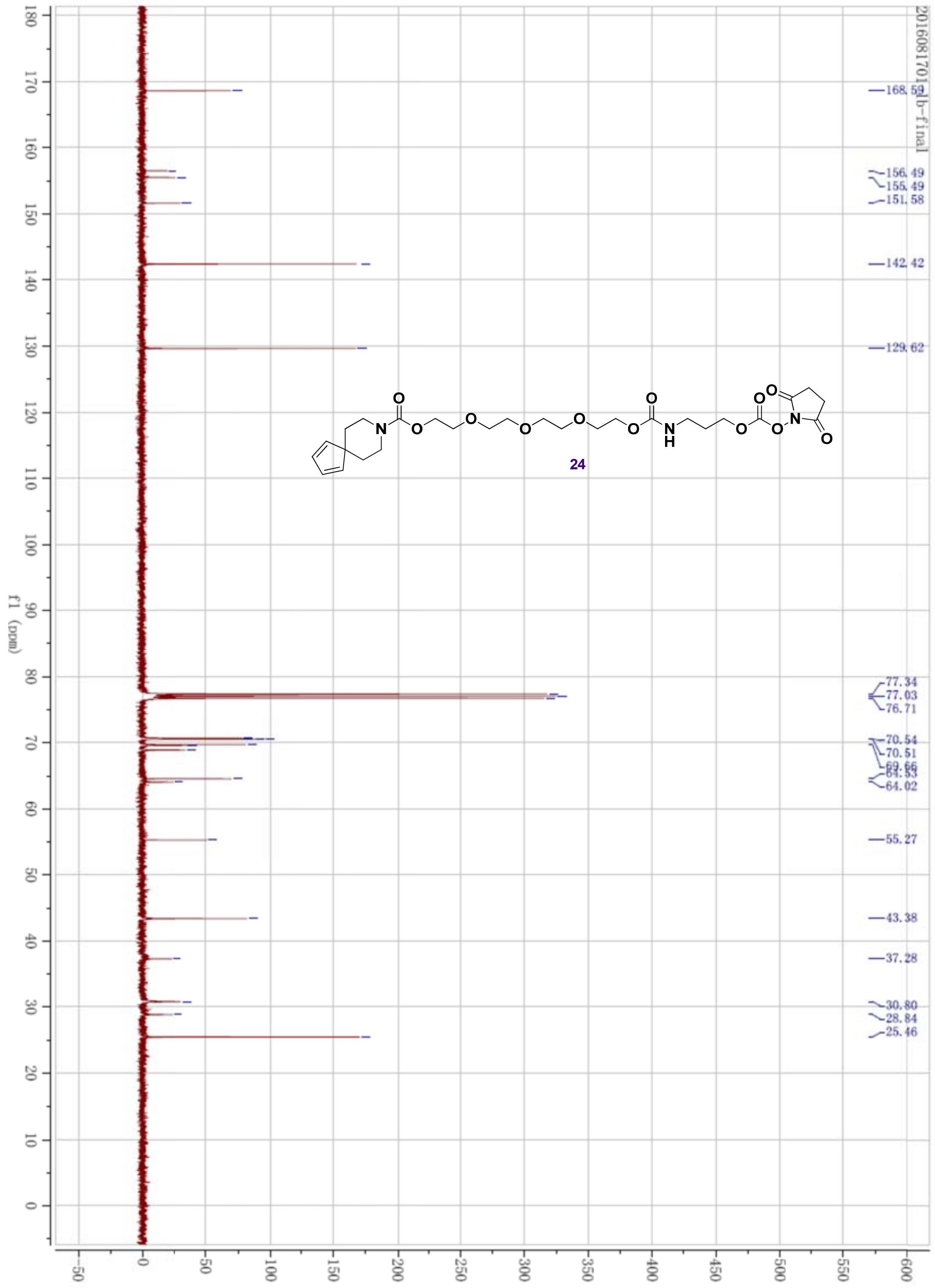
20160817011b-final
168.59
156.49
155.49
151.58
142.42
129.62
77.34
77.03
76.71
70.54
70.51
64.53
64.02
55.27
43.38
37.28
30.80
28.84
25.46
24
f1 (ppm)

#

#

#

#

MS Spectrum

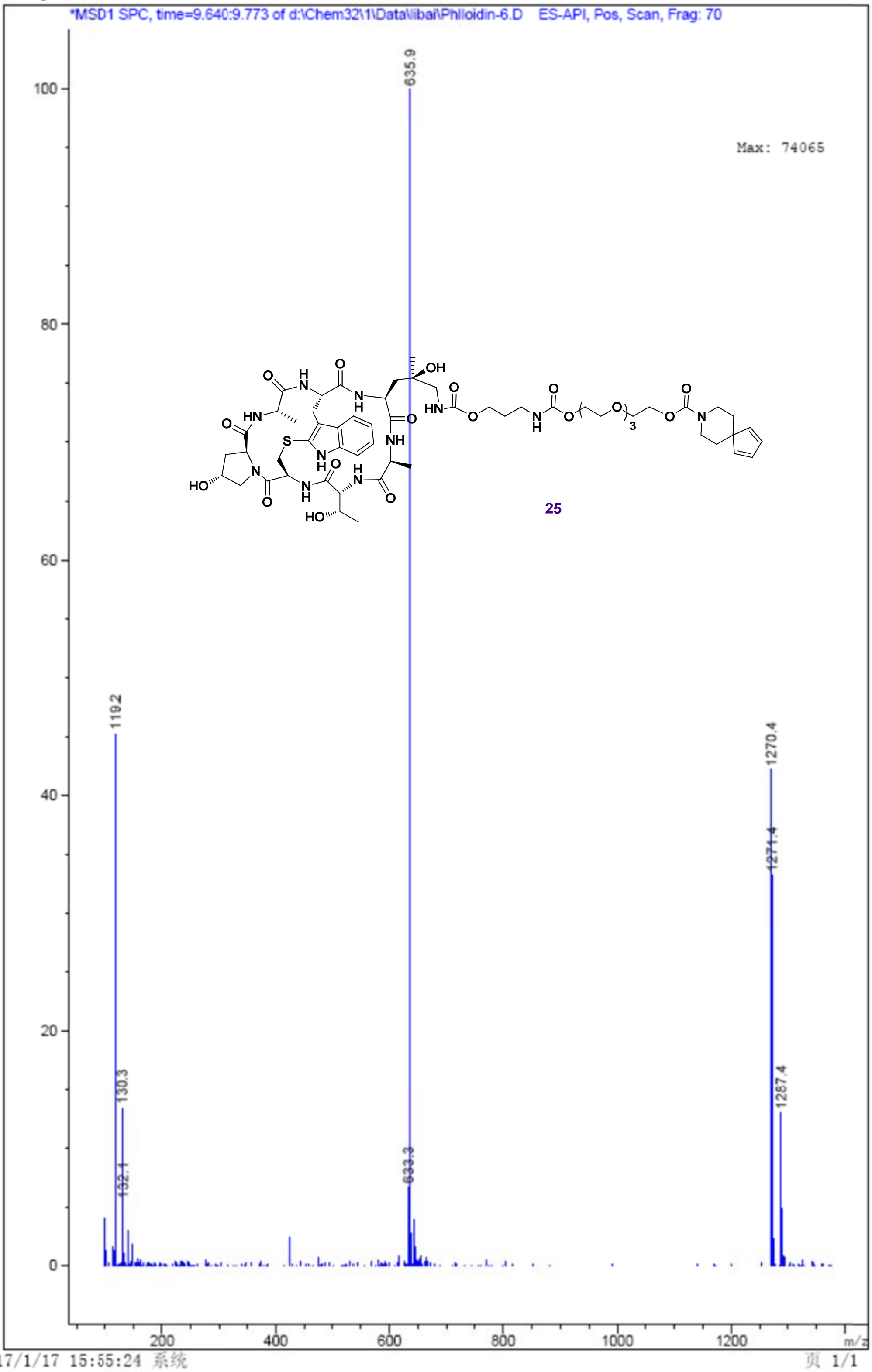

.CMS 2017/1/17 15:55:24 系统 页 1/1

PDF created with pdfFactory Pro trial version www.pdffactory.com

#
